# Deep learning aging marker from retinal images unveils sex-specific clinical and genetic signatures

**DOI:** 10.1038/s41467-026-77102-1

**Published:** 2026-08-26

**Authors:** Olga Trofimova, Leah Böttger, Sacha Bors, Yating Pan, Bart Liefers, Jose D. Vargas-Quiros, Victor A. de Vries, Michael J. Beyeler, David M. Presby, Dennis Bontempi, Janna Hastings, Caroline C. W. Klaver, VascX Consortium, Olga Trofimova, Leah Böttger, Sacha Bors, Bart Liefers, Jose D. Vargas-Quiros, Michael J. Beyeler, David M. Presby, Dennis Bontempi, Janna Hastings, Caroline C. W. Klaver, Ciara Bergin, Bogdan Draganski, Adham Elwakil, Györgyi V. Hamvas, Ilaria Iuliani, Ihor Kuras, Ilenia Meloni, Sofia Ortin Vela, Ian Quintas, Marc Schindewolf, Reinier O. Schlingemann, Mattia Tomasoni, Sven Bergmann, Sven Bergmann

**Affiliations:** 1 Department of Computational Biology, University of Lausanne, Lausanne, Switzerland; 2 Swiss Institute of Bioinformatics, Lausanne, Switzerland; 3 Department of Computational Linguistics, University of Zurich, Zurich, Switzerland; 4 Institute for Implementation Science in Health Care, Faculty of Medicine, University of Zurich, Zurich, Switzerland; 5 Department of Ophthalmology, Erasmus University Medical Center, Rotterdam, the Netherlands; 6 Department of Epidemiology, Erasmus University Medical Center, Rotterdam, the Netherlands; 7 Department of Angiology, Swiss Cardiovascular Center, Inselspital, Bern University Hospital, Bern, Switzerland; 8 Idiap Research Institute, Martigny, Switzerland; 9 Department of Ophthalmology, Radboud University Medical Center, Nijmegen, the Netherlands; 10 Institute of Molecular and Clinical Ophthalmology, University of Basel, Basel, Switzerland; 11 Department of Integrative Biomedical Sciences, University of Cape Town, Cape Town, South Africa; 12 Department of Ophthalmology, University of Lausanne, Fondation Asile des Aveugles, Jules Gonin Eye Hospital, Lausanne, Switzerland; 13 Department of Neurology and University Institute for Diagnostic and Interventional Neuroradiology, Inselspital, University Hospital Bern, University of Bern, Bern, Switzerland; 14 Department of Neurology, Max Planck Institute for Human Cognitive and Brain Sciences, Leipzig, Germany; 15 Department of Clinical Neuroscience, Lausanne University Hospital and University of Lausanne, Lausanne, Switzerland; 16 Platform for Research in Ocular Imaging, Fondation Asile des Aveugles, Jules Gonin Eye Hospital, Lausanne, Switzerland; 17 Department for BioMedical Research, Bern University Hospital, University of Bern, Bern, Switzerland; 18 University Medical Centres, Amsterdam, The Netherlands

**Keywords:** Predictive markers, Genome-wide association studies, Metabolic disorders, Image processing, Machine learning

## Abstract

Retinal fundus images offer a non-invasive window into systemic aging. Here, we fine-tune a foundation model (RETFound) to predict chronological age from color fundus images in 71,343 participants from the UK Biobank, achieving a mean absolute error of 2.85 years. The resulting retinal age gap, i.e. the difference between predicted and chronological age, is associated with cardiometabolic traits, inflammation, cognitive performance, all-cause mortality, dementia, cancer, and incident cardiovascular disease. Genome-wide analyses identify genes related to longevity, metabolism, neurodegeneration, and age-related eye diseases. Sex-stratified models reveal consistent performance but divergent biological signatures: males have stronger links to metabolic syndrome, while in females, both model attention and genetics point to a greater involvement of retinal vasculature. Additional analyses indicate that retinal aging patterns in females vary across the menopausal transition, with postmenopausal females exhibiting higher retinal age gap values and clinical associations that more closely resemble those observed in males. Our study positions the retinal age gap as a biologically relevant and sex-specific phenotype associated with multiple aging-related diseases and outcomes beyond conventional risk factors, including chronological age.

## Abstract (web summary)

Retinal images capture biological features of aging, but their links to health and disease are not fully understood. Here, the authors develop a retinal age predictor and show that accelerated retinal aging is associated with age‑related diseases, mortality and distinct sex‑specific genetic and biological signatures.

## Introduction

Aging is strongly linked to disease susceptibility and mortality risk, yet its biological effects manifest at different rates across individuals—some people experience earlier onset of age-related decline, while others remain relatively healthy even at old age. The ability to measure biological age, distinct from chronological age, has therefore become a growing area of interest in biomedical research^1,2^. Estimators of biological age can offer valuable insights into disease susceptibility, predict health outcomes, and help identify individuals who may benefit from early interventions, such as medications or lifestyle changes^1,2^.

Previous research has shown that deep learning models can estimate chronological age from retinal color fundus images (CFIs) with high accuracy^3–13^. Attention maps of such models reveal that the predictive signal draws on a range of anatomical features, including blood vessels^4,7,11,12^, the macula^4,7,12^, and the optic disc^4,11,12^, suggesting that age-relevant information is distributed across multiple retinal regions. Importantly, the difference between predicted age and true age, known as retinal age gap (RAG), has emerged as a promising phenotype for studying aging-related variation beyond chronological age. Several studies have reported associations between RAG and cardiovascular diseases (CVD)^5,12,14^, chronic obstructive pulmonary disease (COPD)^8^, Parkinson’s disease^15^, cancer^12^, and all-cause mortality^7,12,16^, independent of traditional risk factors. Unlike many established aging biomarkers that require more invasive procedures or costly laboratory assays, retinal age can be estimated cheaply and non-invasively from CFIs. This makes retinal imaging a highly accessible and scalable modality for investigating aging-related variation and its potential relevance for future risk stratification research.

As interest in biological age estimation grows, so does the need to understand how these estimates may vary across demographic groups, particularly between sexes. Research in neuroscience offers a relevant example: studies using brain imaging to estimate biological age have often found that female brains appear younger than male brains on average^17–19^. However, the findings are not fully consistent, as one study reports no sex differences^20^, another finds female brains to appear older^21^, and yet another shows region-specific variations in age estimates^22^. Despite such inconsistencies, the overall evidence supports the importance of stratifying analyses by sex, especially given that the brain age gap is differentially associated with disease patterns and risk factors in females and males^19–22^. In contrast, retinal age studies to date have largely relied on models trained on combined male and female samples, leaving it unclear whether, and how, RAG and its clinical correlates differ between sexes.

Traditionally, deep learning in biomedical imaging has relied on convolutional neural networks (CNNs), but recent advances have introduced Vision Transformers as viable alternatives. While there is no consensus on which architecture performs best overall, evidence suggests performance is task- and data-dependent. For instance, Vision Transformers often outperform CNNs in classification tasks when large datasets and pretraining are available, whereas CNN-based models like nnU-Net continue to excel in 3D medical image segmentation, particularly when rigorously validated and carefully configured^23,24^. RETFound, a foundation model for retinal images pretrained using a masked autoencoder with a large Vision Transformer backbone, has shown strong performance when fine-tuned for disease classification tasks, outperforming other contrastive learning-based approaches^25^. However, RETFound has not yet been applied to age prediction or explored in the context of retinal aging.

**Fig. 1:**
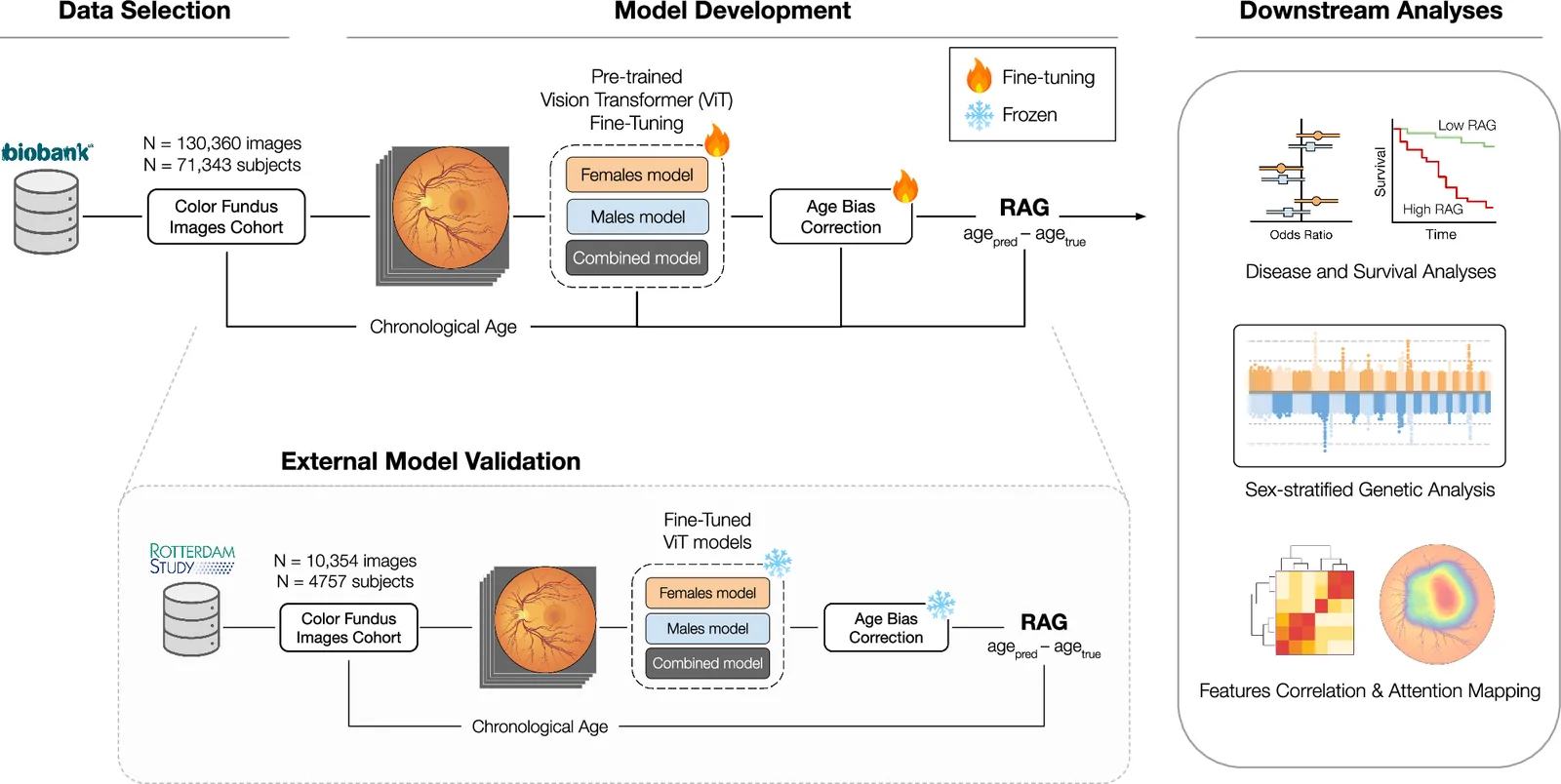
Study overview. We selected a subset of UK Biobank participants with retinal color fundus images that passed quality control. Using 5-fold cross-validation, we fine-tuned RETFound to predict age in a combined sample as well as in male- and female-specific samples. After correcting for regression-to-the-mean bias, we computed the retinal age gap (RAG). We conducted downstream analyses including disease associations, survival analysis, genome-wide association study, correlations between latent and explicit features, and model attention mapping, in both the combined and sex-specific samples. We then tested model performance and replication of genetic associations in an external dataset from the Rotterdam Study. This figure was partially created in BioRender. Trofimova, O. (2026) https://BioRender.com/byhrvsi. Fire icons created by Bahu Icons – Flaticon.com. Snow icons created by Magnific – Flaticon.com.

In this study, we aimed to extend and deepen the current understanding of retinal aging through several complementary analyses (Fig. 1). First, we benchmarked the performance of RETFound in age prediction from CFIs against conventional CNNs. Second, we trained sex-specific models—using separate cohorts of females and males—to investigate whether modeling each sex independently enhanced performance or revealed differences in aging patterns. We then explored the sex-specific value of RAG, particularly with respect to disease associations and genetic correlates. Third, we sought to interpret the internal representations of the model by analyzing attention maps and comparing the model’s latent space to explicit image features. Through these analyses, we aim to better characterize the retinal age gap phenotype, evaluate its associations with aging-related traits and outcomes, and investigate how these associations differ between females and males.

## Results

### RETFound predicts age with high accuracy and generalizes to an external dataset

Fine-tuning RETFound for age prediction yielded consistent performance across the five models trained via five-fold cross-validation (see Supplementary Data 1 for details). After pooling out-of-sample predictions across all folds (*n* = 130,360 fundus images from the UK Biobank (UKB)), the mean absolute error (MAE) was 2.99 years, Pearson’s correlation (*r*) between predictions and chronological ages was 0.88, and the coefficient of determination (R²) was 0.78 (Table 1). When averaging predictions across all available images from each participant at a given time point, the MAE improved to 2.85 years, with an *r* of 0.90 and an R² of 0.80. The model trained on a female-specific cohort achieved an MAE of 3.05 years, an *r* of 0.88, and an R² of 0.78 before averaging across images, which improved to an MAE of 2.92, an *r* of 0.89, and an R² of 0.80 after averaging. Similarly, the male-specific model showed an MAE of 3.09, an *r* of 0.88, and an R² of 0.77 before averaging and improved to an MAE of 2.96, an *r* of 0.89, and an R² of 0.79 after averaging. The MAE difference between female and male models was not significant (*z* = −0.007, *p* = 0.99 for both image- and subject-level MAE).

**Table 1:** Model performance in the UK Biobank out-of-sample test sets and in the Rotterdam Study (external validation)

		UK Biobank					Rotterdam Study				
Sex	Predictions	N	r	MAE	R^2^	RAG mean (SD)	N	r	MAE	R^2^	RAG mean (SD)
Combined	Image-level	130,360	0.88	2.99	0.78	0.19 (3.86)	10,354	0.83	3.58	0.61	−2.07 (4.02)
	Subject-level	71,343	0.90	2.85	0.80	0.18 (3.66)	4757	0.85	3.35	0.66	−1.94 (3.73)
Female	Image-level	58,646	0.88	3.05	0.78	0.01 (3.94)	5849	0.83	3.30	0.66	−1.03 (4.08)
	Subject-level	32,918	0.89	2.92	0.80	0.002 (3.76)	2688	0.85	3.07	0.70	−0.88 (3.80)
	Male-trained model, subject-level	32,918	0.88	3.07	0.77	0.64 (3.89)	2688	0.84	3.47	0.62	−2.01 (3.88)
	Male-trained model, subject-level, corrected	32,918	0.88	3.36	0.73	0.73 (4.27)	2688	0.84	3.50	0.61	0.67 (4.38)
Male	Image-level	58,512	0.88	3.09	0.77	0.09 (4.00)	4505	0.83	3.86	0.56	−2.68 (4.08)
	Subject-level	32,918	0.89	2.96	0.79	0.07 (3.81)	2069	0.85	3.61	0.61	−2.51 (3.76)
	Female-trained model, subject-level	32,918	0.88	3.08	0.77	−0.66 (3.89)	2069	0.86	3.19	0.70	−1.51 (3.69)
	Female-trained model, subject-level, corrected	32,918	0.88	3.54	0.71	−0.83 (4.41)	2069	0.86	3.53	0.62	−0.81 (4.36)

Performance for female retinas evaluated on male-trained models, and vice versa, is also shown after applying age bias correction, to account for potential age distribution differences between females and males. MAE: mean absolute error, r: Pearson’s correlation coefficient, R^2^: coefficient of determination, RAG: retinal age gap, SD: standard deviation.

To assess the generalizability of our models beyond the UKB, we tested their performance on independent sets of CFIs from the Rotterdam Study (RS). The image-level MAE was 3.58 years in the combined sample, 3.30 in females, and 3.86 in males (see Table 1). To quantify variability across models, we calculated the mean per-image standard deviation (SD) of the five model predictions, which was 0.99 years in the combined sample (28% of the MAE), 1.19 years in females (36% of the MAE), and 1.12 years in males (29% of the MAE). The subject-level MAE was 3.35 years in the combined sample, 3.07 years in females, and 3.61 years in males. The MAE was not significantly different between sexes, neither at the image level (*z* = −0.097, *p* = 0.92), nor at the subject level (*z* = −0.101, *p* = 0.92). While the squared correlation between true and predicted ages was 0.72 for the three models, the R² values were notably lower (0.61–0.70), reflecting a systematic bias in age prediction. The mean RAG values were −2.51 years in males, −0.88 years in females, and −1.94 years in the combined sample, indicating that the model consistently underestimated age in the RS, especially in males.

In both the UKB and the RS, predicted age tended to be overestimated in younger individuals and underestimated in older individuals (Supplementary Fig. 1a), a regression-to-the-mean pattern commonly observed in aging biomarker studies^26^. Therefore, we applied the bias correction method proposed by Cole and colleagues^18^, which rendered predicted age error orthogonal to chronological age (see Supplementary Fig. 1b).

### Retinal age gap is consistently higher in postmenopausal females

In the UKB combined cohort, males had a lower RAG than females on average (∆ = 0.28, *t* = 8.9, *p* = 7e-19, Cohen’s *d* = 0.069; see Supplementary Fig. 2a), indicating that the model estimated male retinas as appearing younger, though the effect was small. Additionally, cross-application of sex-specific models revealed that male retinas evaluated using the female-trained model had a mean RAG of −0.83, whereas female retinas evaluated using the male-trained model had a mean RAG of 0.73 after applying age bias correction (see Table 1), consistent with the observation that the models tended to estimate male retinas as appearing younger than female retinas. Given this somewhat unexpected sex difference, opposite to what might be anticipated based on lifespan trends, we explored whether it could be explained by anatomical differences, such as overall ocular size, or by life-stage transitions associated with menopause. Since body height is known to correlate with ocular dimensions^27^, we regressed standing height out of the RAG. This adjustment attenuated the sex difference in RAG, which nonetheless remained significant (∆ = 0.16, *t* = 5.0, *p* = 6e-07, Cohen’s *d* = 0.040; see Supplementary Fig. 2b). Height and height-squared were therefore included as covariates in all subsequent analyses to account for potential confounding. Stratified analyses in females revealed that RAG was higher in postmenopausal than premenopausal females (∆ = 0.60, *t* = 10.7, *p* = 1e-26, *d* = 0.153), with male RAG positioned in between these groups (see Supplementary Fig. 3a). Similar patterns were observed when stratifying females by age (<55 vs ≥55 years; Supplementary Fig. 3b), with older females exhibiting higher RAG than younger females despite the orthogonalization of RAG to chronological age.

In the RS, we observed a similar pattern: males had lower RAG than females overall (∆ = 0.37, *t* = 2.9, *p* = 0.004, Cohen’s *d* = 0.085). When height was regressed out of the RAG, the sex difference was again reduced but not removed (∆ = 0.30, *t* = 2.4, *p* = 0.015, Cohen’s *d* = 0.071). Cross-application of sex-specific models yielded consistent results, with male retinas evaluated using the female-trained model showing a mean RAG of −0.81, and female retinas evaluated using the male-trained model showing a mean RAG of 0.67. When stratifying females by age (<55 vs ≥55 years), RAG was higher in older females than in younger females (∆ = 0.40, *t* = 2.29, *p* = 0.02, *d* = 0.09) or males (∆ = 0.51, *t* = 3.59, *p* < 0.001, *d* = 0.12), while there was no significant difference between younger females and males (∆ = 0.11, *t* = 0.63, *p* = 0.53, *d* = 0.02). These findings replicate the UKB results and further support the observation that retinal age predictions tended to estimate postmenopausal/older female retinas as appearing older than retinas of males and premenopausal/younger females.

### Retinal age gap is associated with metabolic, inflammatory, and ocular health indicators

**Fig. 2:**
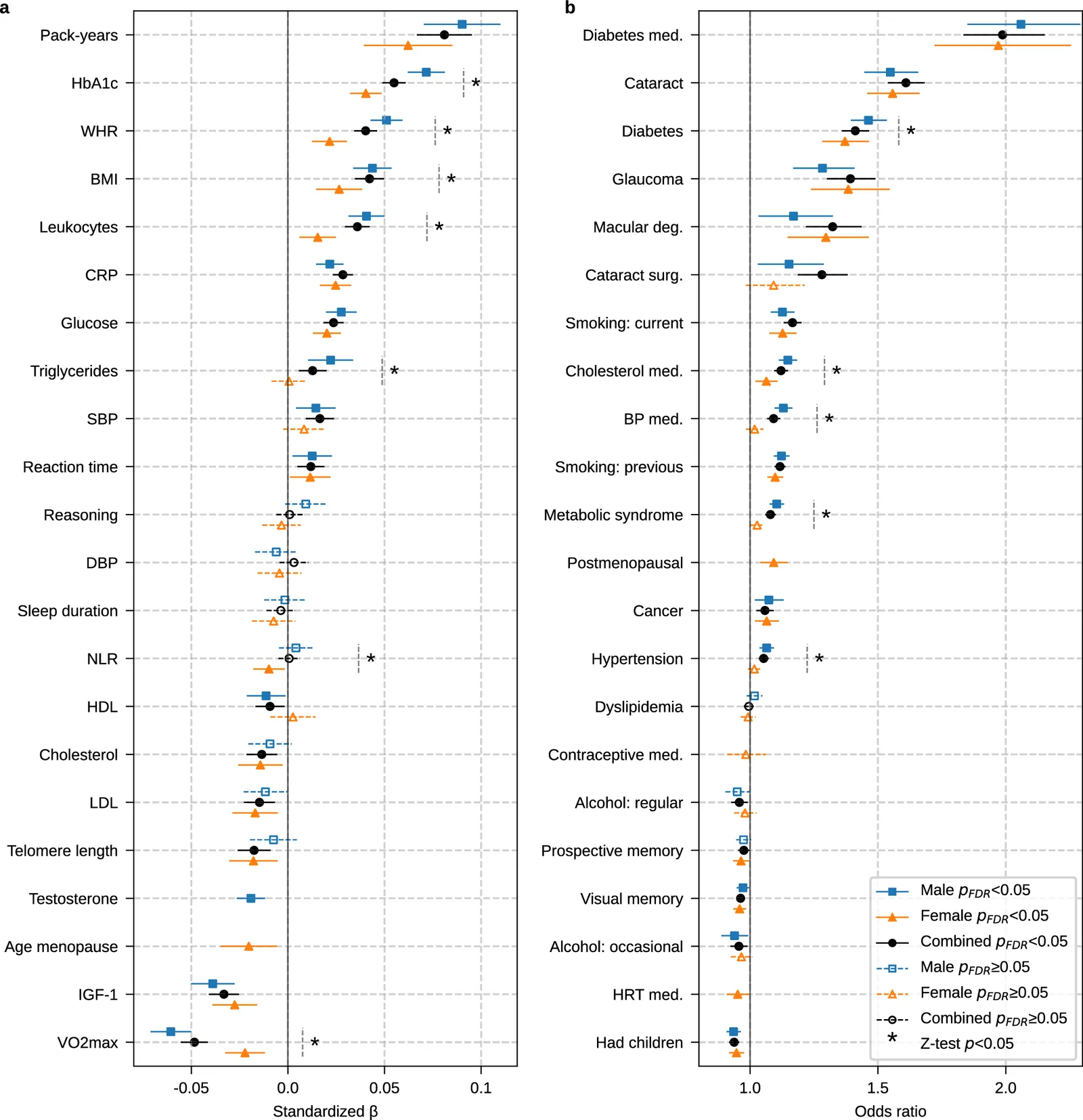
Standardized beta coefficients and odds ratios from models linking retinal age gap to risk factors and diseases. **a**, **b** Ordinary least square linear regression **a** and logistic regression **b** models were adjusted for socio-demographic factors. Outcomes are ordered by average effect size between the three sex categories. Error bars represent the 95% confidence interval. Female- and male-specific effects were compared using a two-sided *z*-test. Sample sizes and detailed results are shown in Supplementary Data 2–3. BMI: body mass index, BP: blood pressure, CRP: C-reactive protein, DBP: diastolic blood pressure, deg.: degeneration, FDR: false discovery rate, HDL: high-density lipoprotein, HRT: hormone replacement therapy, IGF-1: insulin-like growth factor 1, LDL: low-density lipoprotein, med.: medication, NLR: neutrophil-to-lymphocyte ratio, SBP: systolic blood pressure, surg.: surgery, WHR: waist-to-hip ratio. Source data are provided as a Source Data file.

After adjusting for socio-demographic variables, higher RAG was significantly associated with increased pack-years of smoking, glycated hemoglobin (HbA1c), waist-to-hip ratio, body mass index (BMI), leukocyte count, C-reactive protein (CRP), glucose, triglycerides, systolic blood pressure (SBP), and slower reaction time in the combined sample (Fig. 2a and Supplementary Data 2 for detailed results). Conversely, RAG was negatively associated with VO₂max, insulin-like growth factor 1 (IGF-1), telomere length, low-density lipoprotein (LDL), high-density lipoprotein (HDL), and total cholesterol. Sex-specific associations generally mirrored the combined results, although males exhibited larger effect sizes across most biomarkers except CRP. Associations found in females but not males comprised neutrophil-to-lymphocyte ratio, LDL, total cholesterol, telomere length, and age at menopause, while male-specific associations included testosterone, SBP, triglycerides, and HDL. Absolute standardized effect sizes ranged from 0.01 to 0.09, reflecting the change in SD units of biomarker levels per SD increase in RAG. Likelihood ratio tests (LRTs) comparing nested models with and without RAG confirmed that inclusion of RAG significantly improved model fit for all reported associations (Supplementary Data 2).

For binary health outcomes, in the combined sample, higher RAG was associated with diabetes medication use, a history of cataract, diabetes, glaucoma, macular degeneration, cataract surgery, current or previous smoking status, use of cholesterol or blood pressure medications, metabolic syndrome, cancer (ever diagnosed), and hypertension (Fig. 2b and Supplementary Data 3). Negative associations were observed with having children, visual and prospective memory performance, and alcohol consumption. Among females, postmenopausal status was associated with higher RAG, whereas hormone replacement therapy correlated with lower RAG. Males showed stronger associations than females with metabolic traits, including a male-specific association with metabolic syndrome, hypertension, and blood pressure medication. Odds ratios ranged from 0.94 to 2.06 per SD increase in RAG. LRTs confirmed that the inclusion of RAG significantly improved model fit for all reported associations (Supplementary Data 3).

To further explore potential hormonal influences, we repeated association analyses in females stratified by menopausal status and, separately, by age (<55 vs ≥55 years) (Supplementary Figs. 4–5 and Supplementary Data 2–3). Overall, associations between higher RAG and adverse cardiometabolic and inflammatory biomarkers were stronger in postmenopausal/older females than in premenopausal/younger females, rendering the former more similar to males in effect size patterns. Notable exceptions included sleep duration, which was associated with lower RAG only in premenopausal/younger females, and cataract, macular degeneration, cancer, and hypertension, for which associations were stronger in premenopausal/younger females. Interestingly, triglycerides, LDL, and total cholesterol were associated with lower RAG specifically in postmenopausal/older females, a pattern not observed in premenopausal/younger females or in males.

### Retinal age gap is prospectively associated with mortality and incident disease with sex-specific patterns

**Fig. 3:**
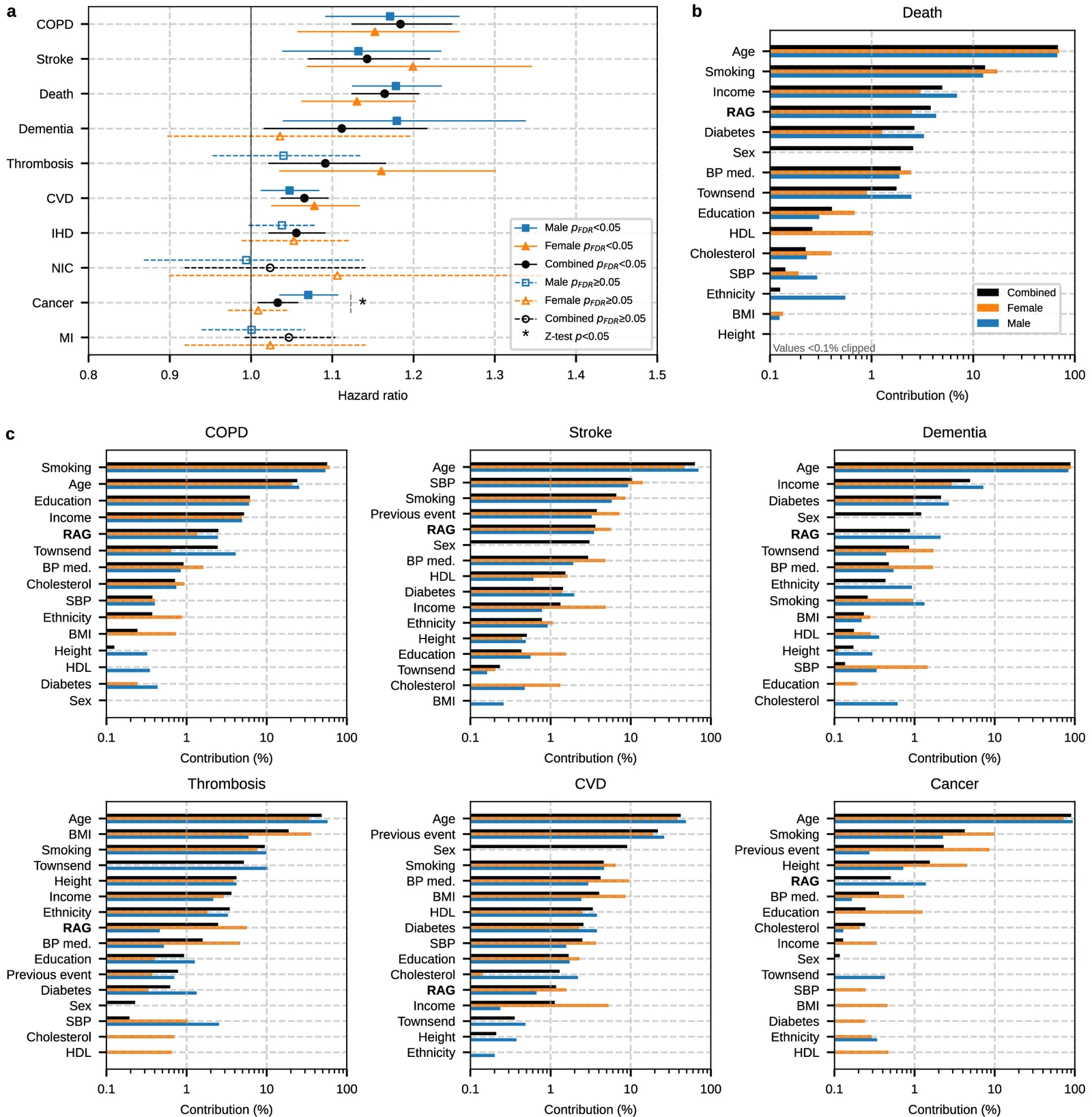
Retinal age gap associations with mortality and disease outcomes. **a** Hazard ratios (HR) from Cox proportional hazards regression analyses linking retinal age gap to mortality and incident diseases in the combined, female, and male samples. Models were adjusted for socio-demographic factors, common cardiovascular risk factors, and previous events from the same category. Outcomes are ordered by average HR between the three sex categories. Error bars represent the 95% confidence interval. Female- and male-specific effects were compared using a two-sided *z*-test. Sample sizes, number of events, and detailed results are shown in Supplementary Data 4. **b** Relative contribution of predictors to mortality risk in the multivariable Cox model, quantified as the percent change in model χ² when each variable is removed from the model. **c** Relative contribution of predictors to disease risk for selected outcomes using the same leave-one-out approach as in **b**. Additional cardiovascular outcomes (IHD, NIC, and MI) are shown in Supplementary Fig. 6. Detailed results are presented in Supplementary Data 5. BMI: body mass index, BP med.: blood pressure medication, COPD: chronic obstructive pulmonary disease, CVD: cardiovascular disease, FDR: false discovery rate, HDL: high-density lipoprotein, IHD: ischemic heart disease, MI: myocardial infarction, NIC: non-ischemic cardiomyopathy, RAG: retinal age gap, SBP: systolic blood pressure. Source data are provided as a Source Data file.

In survival analyses adjusted for socio-demographics, common cardiovascular risk factors, and previous events of the same category, RAG was significantly associated with all-cause mortality as well as incidence of CVD, COPD, stroke, dementia, thrombosis, ischemic heart disease, and cancer in the combined sample (see Fig. 3a and Supplementary Data 4). When stratified by sex, thrombosis was associated with RAG in females but not males, and hazard ratios (HRs) for CVD and stroke were larger in females, although not significantly. In contrast, RAG predicted dementia and cancer incidence uniquely in males and was more strongly associated with all-cause mortality in males, though confidence intervals were large and the sex difference was only significant for cancer. HRs ranged from 1.03 to 1.20 per SD increase in RAG. LRTs on nested models confirmed that inclusion of RAG significantly improved model fit for all reported survival associations (Supplementary Data 4). Comparisons of model discrimination showed statistically significant improvements in C-index for all-cause mortality in all samples, as well as for stroke and COPD in the combined and male samples. For other outcomes, improvements in C-index were modest and not statistically significant despite significant improvements in model fit according to LRTs, consistent with findings that strong baseline predictors such as age and sex can limit changes in C-index for otherwise meaningful clinical predictors^28^.

To further evaluate the importance of RAG relative to other predictors, we performed a leave-one-out analysis of the Cox models in which each covariate was removed individually and the resulting reduction in model fit was quantified. As expected, chronological age was the strongest contributor for most outcomes, while smoking was the dominant predictor for COPD. Notably, RAG ranked among the five largest contributors for five outcomes—mortality, COPD, stroke, dementia, and cancer (Fig. 3b–c and Supplementary Data 5). In contrast, RAG contributed less to prediction of overall CVD, where classical cardiovascular risk factors accounted for a larger share of the model’s predictive performance (Fig. 3c, Supplementary Fig. 6, and Supplementary Data 5).

When stratifying females by menopausal status or age group (Supplementary Fig. 7), most incident disease associations were driven by postmenopausal/older females, whereas associations in premenopausal/younger females were generally weaker and often not statistically significant, possibly reflecting both biological differences and lower event counts in the younger stratum. An exception was all-cause mortality, which showed significant effect in both younger and older females.

### Sex-stratified GWAS reveals shared and distinct genetic associations for retinal age gap

**Fig. 4:**
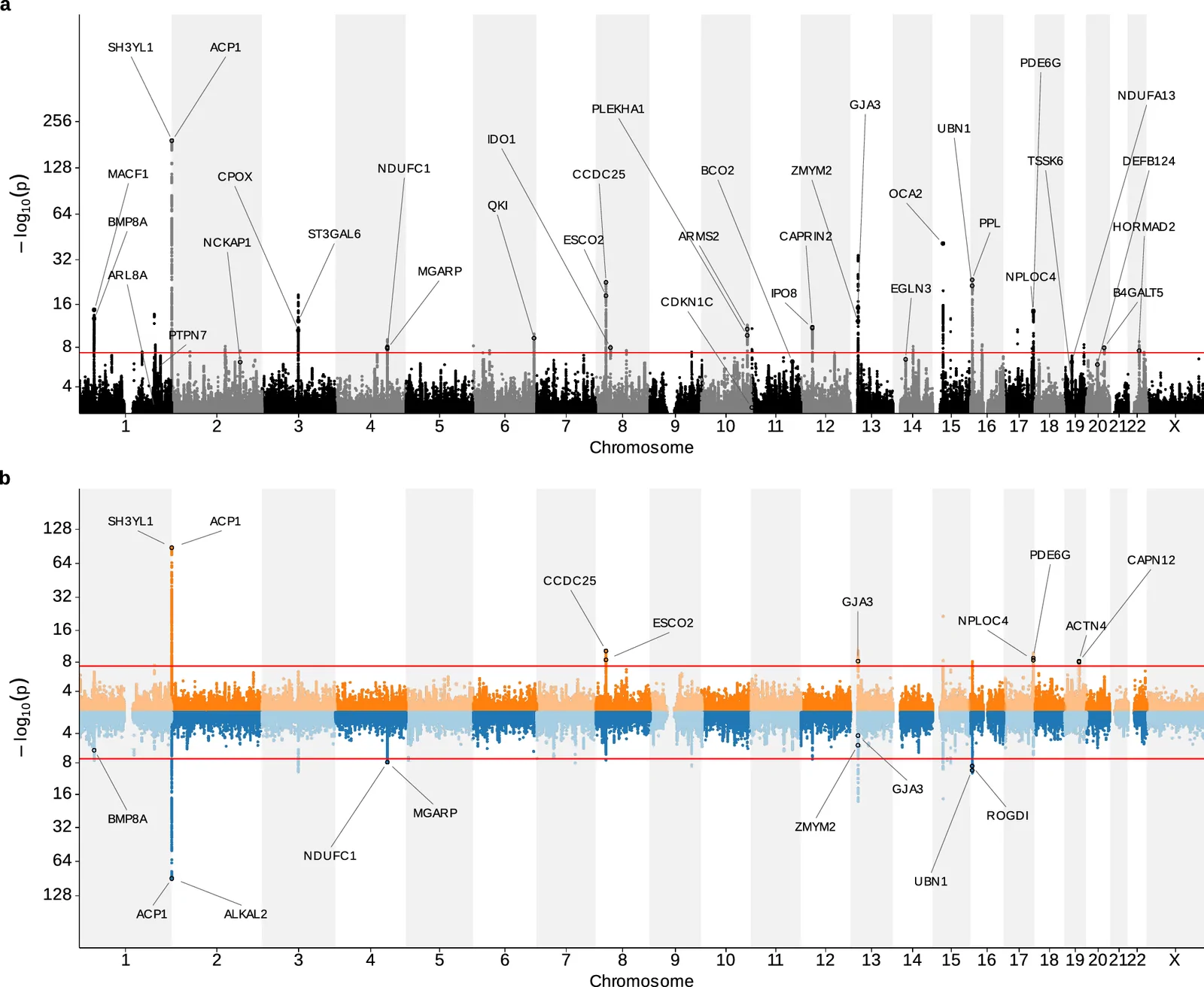
Manhattan plots from the genome-wide association study (GWAS) of retinal age gap, annotated with top genes. **a** Manhattan plot of the combined-sample GWAS (*n* = 68,137). **b** Miami plot showing the female-specific GWAS (top, orange; *n* = 30,698) and male-specific GWAS (bottom, blue; *n* = 30,958). -log_10_(*p*) values are plotted on a log_2_ scale. The red lines indicate genome-wide significance (5×10^−8^). Each dot corresponds to a single nucleotide polymorphism. The annotated genes correspond to those identified as significant by the PascalX gene-based analysis after Bonferroni correction for multiple testing (*p* < 2.61×10^−6^ in **a** and *p* < 2.51×10^−6^ in **b**). When more than two genes were identified at the same cytogenetic location, only the top two signals are labeled for clarity (the full gene lists are shown in Supplementary Data 9–11). Source data are provided as a Source Data file.

The genome-wide association study (GWAS) of RAG in the UKB identified 40 genome-wide significant loci in the combined sample (Fig. 4a and Supplementary Data 6), 10 loci in females (Fig. 4b, top and Supplementary Data 7), and 14 loci in males (Fig. 4b, bottom and Supplementary Data 8). Gene-wise analysis using PascalX^29^ revealed 75 genes across 23 independent linkage disequilibrium (LD) blocks in the combined sample, 18 genes in 5 LD blocks in females, and 16 genes in 5 LD blocks in males, with an overlap of 5 genes between females and males (see Supplementary Data 9–11).

In the combined sample, the strongest SNP association was observed for rs2290911, mapping within *SH3YL1* on chromosome (chr) 2. Gene-level analysis further revealed prominent signals for *ACP1*, *ALKAL2*/*FAM150B*, and *FAM110C*, which are located in close proximity to *SH3YL1* within the 2p25.3 region. The next strongest signal was found for rs1800407 on chr 15, located within *OCA2*. This was followed by rs11840593 on chr 13, in proximity to *GJA3*. Other notable signals included rs75804753 on chr 16 near *UBN1* and *PPL*, rs13267051 on chr 8 near *CCDC25* and *ESCO2*, rs10935473 on chr 3 within *ST3GAL6*, rs61779328 on chr 1 near *BMP8A* and *MACF1*, and rs3432 near *PDE6G* and *NPLOC4*. The sex-specific analyses revealed distinct genetic associations not observed in the combined sample. In females, unique signals included variants rs12714397 (chr2) and rs8182571 (chr19), and genes *CAPN12* and *ACTN4* (both on chr 19), the latter featuring the variant rs8182571. In contrast, rs79665333 (chr5), rs145132834 (chr7), and rs28844699 (chr15) were identified exclusively in males. Complete results are reported in Supplementary Data 6–11, and quality control plots for the GWAS analyses are presented in Supplementary Fig. 8. The genomic control factor (λGC) was 1.14, 1.06, and 1.07 for the combined, female, and male GWAS, respectively, indicating modest inflation in the combined analysis and well-calibrated test statistics in the sex-stratified analyses.

The SNP-based heritability (h^2^_SNP_) of RAG, estimated using GREML, was 0.277 ( ± 0.009) for the combined sample, 0.278 ( ± 0.019) for females, and 0.247 ( ± 0.018) for males. The sex difference in h^2^_SNP_ was not significant (*z* = 1.15, *p* = 0.25).

In the RS, 7 of 29 SNPs (11 unavailable) and 25 of 71 genes (4 unavailable) identified in the UKB combined model analysis also reached significance in the replication analysis (Supplementary Fig. 9 and Supplementary Data 12–13). The top SNPs in the RS, rs2290911 (chr2) and rs1800407 (chr15), were likewise the strongest signals in the UKB. Similarly, the top three genes, *ACP1*, *ALKAL2*/*FAM150B*, and *SH3YL1*, were identical across both cohorts and remained genome-wide significant in the RS. The remaining signals showed consistent directions of effect (see Supplementary Fig. 9c), with enrichment beyond chance even when not significant, supporting robust genetic replication of RAG in the independent RS cohort.

### Saliency and attention maps highlight the optic disc, macular area, and blood vessels

**Fig. 5:**
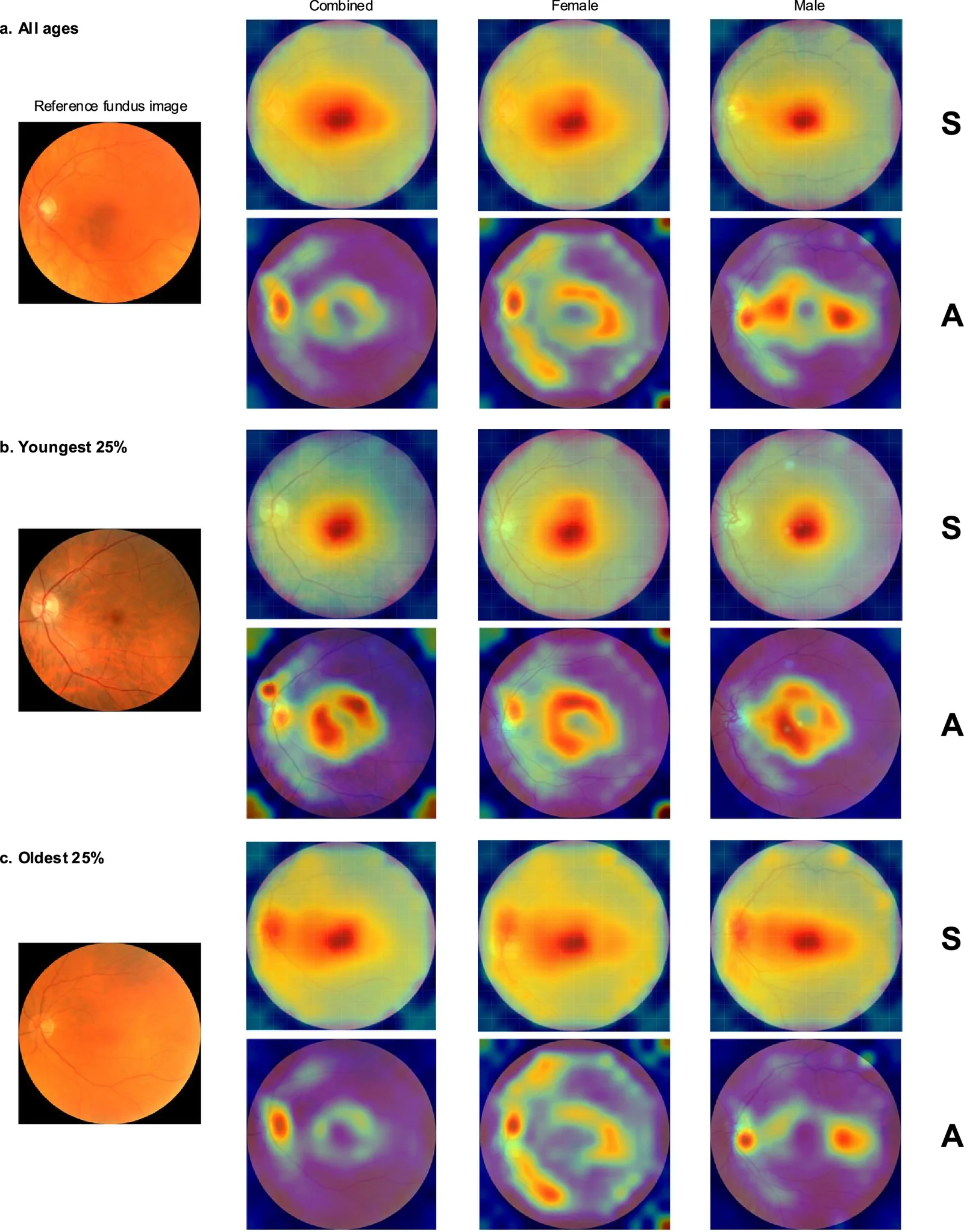
Group-average saliency and attention maps of the fine-tuned models on left eye images. **a–c** Smoothed pixel-level saliency (S, upper rows) and attention (A, lower rows) maps of fold 1 models, obtained by bicubic interpolation from patch-level maps, are projected on a reference fundus image for **a** all ages (*n* = 13,078 images combined sample, 5897 females and 5838 males), **b** the youngest quartile (*n* = 3270 combined, 1475 females and 1460 males), and **c** the oldest quartile (*n* = 3269 combined, 1474 females and 1460 males). Reference fundus images reproduced by kind permission of UK Biobank ©.

To better understand what the models rely on to predict age, we first computed saliency maps from the fine-tuned models using RELPROP^30^, as in the original RETFound publication^25^. Saliency maps showed the strongest activation in the macula and the temporal half of the optic disc, with sex-specific models displaying patterns highly similar to those of the combined-sample models (Fig. 5a, top row). A comparison between the youngest and oldest age quartiles revealed distinct patterns: in younger retinas, saliency was concentrated primarily in the macula (Fig. 5b, top row), whereas in older retinas, both the macula and the temporal optic disc contributed to the model’s predictions (Fig. 5c, top row). Saliency maps were highly consistent across model folds and showed strong symmetry between left and right eyes (Supplementary Fig. 10).

Attention maps extracted from attention blocks of the Vision Transformer were partially consistent with the saliency maps, highlighting the temporal optic disc across all groups, especially in the oldest quartile. However, unlike the saliency maps, attention maps emphasized the area surrounding the macula rather than the macula itself (Fig. 5, bottom rows). Additionally, the main vascular arches emerged as prominent regions of attention, particularly in female participants in the oldest quartile (Fig. 5, middle column, bottom row), suggesting that the model may rely more heavily on vascular features when predicting age in older female retinas. As observed with saliency maps, attention maps exhibited strong left–right eye symmetry, although they showed greater variability across model folds (see Supplementary Fig. 11). Detailed maps from the 16 individual attention heads of the last block are shown in Supplementary Figs. 12–14.

### Model latent variables reflect vascular density, diameter, and bifurcations

**Fig. 6:**
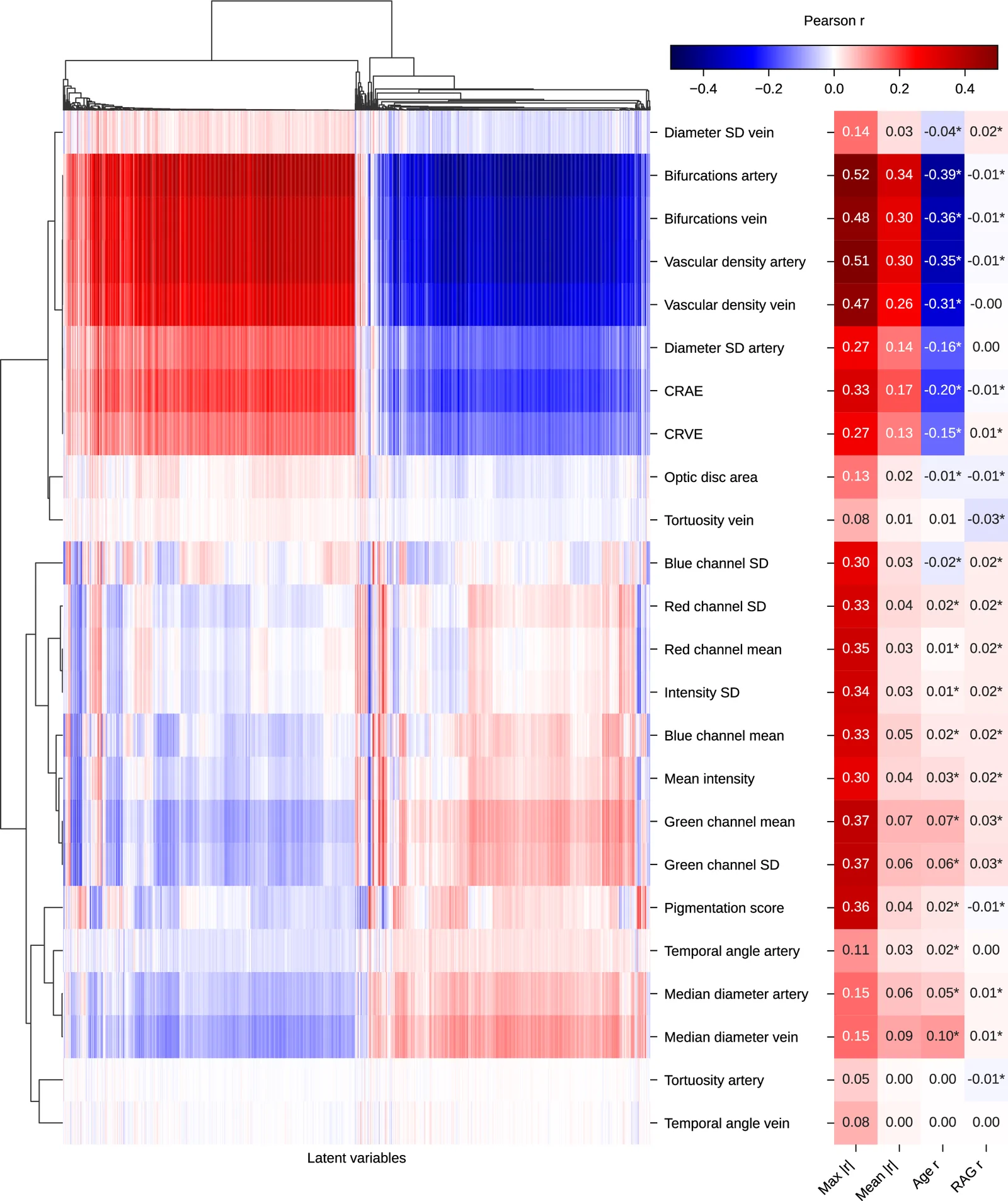
Correlation map between the model’s 1024 latent variables and interpretable image features, in the combined sample for fold 1 (*n* = 24,066 images). For every image feature, the maximum and mean absolute correlation with latent variables, the correlation with chronological age and retinal age gap are shown on the right side of the figure. CRAE: central retinal arterial equivalent, CRVE: central retinal venous equivalent, RAG: retinal age gap, SD: standard deviation, *: uncorrected *p* < 0.05.

We then examined the correlations between the model’s latent variables (LVs) and interpretable image features, including vascular morphology, optic disc area, and RGB statistics. Most LVs were correlated with vascular density and bifurcation count (mean |*r* | ∈ [0.26, 0.34]). Notable correlations were also observed with central retinal arterial and venous equivalents (mean |*r* | = 0.17 and 0.13), artery diameter SD (mean |*r* | = 0.14), and to a lesser extent, median vascular diameters, and green channel mean and SD (mean |*r* | ∈ [0.06, 0.14]) (Fig. 6). The correlation pattern was highly consistent across all 15 models (5 folds × 3 groups), with correlation matrices showing strong agreement across folds and sex groups (Supplementary Figs. 15–28).

Interestingly, the correlation between image features and chronological age (shown for each feature in the penultimate column of Fig. 6) closely mirrored their correlation with the LVs. Vascular features such as density and bifurcations showed stronger correlations with age than with the LVs, while the opposite pattern was seen for RGB-based features, which correlated more with LVs than with age. This suggests that although the model’s internal representations reflect vascular aging to some extent, they also encode additional non-age-related variation, particularly in color distribution and intensity, that may contribute to its age estimates.

By contrast, correlations between these features and RAG were weak (rightmost column of Fig. 6), indicating that the residual retinal age signal captured by RAG is not directly explained by the explicit vascular and color features examined here. This does not exclude the possibility that more complex, nonlinear, or spatially localized vascular patterns contribute to RAG but are not adequately captured by the predefined features analyzed in this study.

## Discussion

In this study, we fine-tuned RETFound to predict chronological age from retinal images, demonstrating robust generalization to an independent dataset with improved accuracy through averaging predictions from both eyes. The resulting retinal age gap (RAG)—the difference between predicted retinal age and chronological age—represents individual deviation from the average relationship between retinal appearance and chronological age in the study population. We also trained sex-specific models and found differences in RAG and its associations with clinical outcomes and genetic variants between females and males, with additional analyses suggesting that retinal aging patterns in females vary across the menopausal transition. RAG was associated with key health outcomes including CVD, cancer, and death, as well as eye disease, metabolic and inflammatory biomarkers. Genetic analyses identified novel and previously reported associations involved in aging-relevant pathways. Attention maps and latent space analyses pointed to the temporal optic disc, macular area, and blood vessels as important regions, and revealed correlations with vascular features.

With an MAE of 2.85 years and an R^2^ of 0.80, RETFound fine-tuned to predict age on UKB data outperformed previous CNN-based models, which have reported MAE between 3.08 and 4.5 years and R^2^ between 0.51 and 0.77^3,6,7,9,10^. Although foundation models require considerable computational resources to pretrain, our results suggest they offer a measurable benefit in downstream regression tasks such as age prediction. When applied to the independent RS dataset, the model maintained a high correlation between predicted and true age but exhibited a consistent downward bias, especially in males. This systematic underestimation may be partially explained by the slightly older age distribution in the RS cohort (on average 0.3 years older than the UKB), combined with regression-to-the-mean effects that tend to produce larger underestimations at the upper end of the age range prior to bias correction. Furthermore, the lower age predictions in the RS could be due to technical differences between datasets, such as the camera model or image acquisition settings, or it may reflect genuine differences in participants’ health, whereby RS retinas may appear biologically younger than UKB retinas.

Consistent with previous findings^5,9^, we show that averaging predictions across images from both eyes improves accuracy, likely by reducing noise arising from variations in image quality or other image parameters. While this strategy may not be ideal in clinical datasets, as it may obscure pathologies affecting only one eye, or with asymmetrical disease progression, it is well-suited to address image quality variability in population-level datasets like the UKB. Therefore, the choice to average or not the predictions coming from both eyes should be guided by the specific research question and context of a given study.

We initially observed a modest but consistent difference in predicted retinal age between sexes across cohorts, with male retinas estimated as appearing younger on average, even after adjusting for potential body size confounding. However, the stratified analyses suggested a more nuanced, life-stage-dependent pattern. Specifically, RAG was lower in younger or premenopausal females but higher in older or postmenopausal females, placing males between these two groups. This pattern parallels epidemiological observations in which females experience lower cardiometabolic risk prior to menopause, followed by a marked increase afterward, including a rise in CVD incidence^31^. Such transitions may influence retinal vascular and metabolic features that the model captures as aging-related signals. More broadly, these findings highlight the importance of considering the menopausal transition alongside sex in aging research.

Associations between RAG and cardiometabolic risk factors were stronger in males, consistent with the higher prevalence of metabolic syndrome and related conditions in men during midlife^32^. However, when females were stratified by menopausal status or age, the pattern of associations in older or postmenopausal females became more similar to that observed in males, suggesting a convergence in risk profiles after the menopausal transition. In contrast, several traits, including short sleep duration, cancer, hypertension, cataract, and macular degeneration, showed stronger associations with RAG in younger or premenopausal females. One possible explanation is that elevated RAG at younger ages may reflect early manifestation of conditions that typically emerge later in life, making them particularly informative markers of accelerated biological aging in this group. By comparison, in older or postmenopausal females, RAG may more strongly capture cumulative cardiometabolic and inflammatory processes that become more pronounced after the loss of estrogen’s protective effects^33^.

Interestingly, while cross-sectional associations between RAG and health indicators were generally stronger in males, RAG predicted incident CVD similarly in males and females, after adjusting for conventional risk factors such as BMI, smoking, SBP, cholesterol, and diabetes. In fact, HRs were higher in females for stroke, thrombosis, and overall CVD, though not significantly so, likely due to limited statistical power (Supplementary Data 4). Consistent with this pattern, the relative contribution of RAG to model prediction for these outcomes was also greater in females compared with males and the combined sample. While males have a higher overall risk of CVD [29], our findings suggest that this difference may be largely captured by traditional risk factors included in the models. In contrast, the additional variance captured by RAG may be more informative in females, potentially reflecting sex-specific biological aging processes not well represented by traditional risk metrics. For example, women exhibit higher baseline inflammation, including elevated CRP and interleukin-6, which has been linked to greater CVD risk compared to men, even after adjusting for risk factors like smoking and BMI^34^. Hormonal dynamics and sex-specific metabolic changes in females may contribute to patterns of aging that are not well captured by conventional risk metrics, but are more readily reflected in RAG.

Conversely, RAG predicted future cancer and dementia risk in males but not in females. The association with cancer may reflect known biological differences: male cells experience greater oxidative stress and accumulate more somatic mutations than female cells^35^, both of which increase susceptibility to malignant transformation and represent hallmarks of aging that may be captured by RAG. For dementia, we pooled all dementia subtypes due to limited case numbers, but we hypothesize the observed association may have been driven primarily by vascular dementia, given its shared link with metabolic syndrome and cardiovascular risk factors^36^.

Genetic analyses of RAG revealed both novel loci and replication of previously reported associations^9,10,37^. Novel loci had been previously associated with corneal resistance factor (rs1200105)^38^, educational attainment (rs79073127 and rs6477724)^39^, and skin and eye cancer (rs12203592 and rs2413887)^40,41^. At the gene level, unlike traditional approaches that link only the most significant SNP to the nearest gene, our method aggregates signals across all SNPs within each gene region while accounting for LD, thereby improving sensitivity to detect biologically meaningful associations^29^. The genes we identified have been implicated in metabolism, lifespan, and retinal vessel density (*ACP1*^42,43^, *SH3YL1*^43–45^, *ALKAL2*^43,46^, *MACF1-BMP8A*^47^), in age-related eye diseases and retinal vessel diameter variability (*GJA3*^48^, *ARMS2*^49^, *NPLOC4*-*PDE6G*^43,50^), cellular senescence (*UBN1*)^51^, Alzheimer’s disease (*CCDC25-ESCO2*)^52^, and immune function (*PTPN7*)^53^ among others. This is consistent with the clinical traits linked to RAG, supporting its biological relevance. Like previous retinal age studies^9,10,37^, we also identified *OCA2*, a gene involved in eye pigmentation, suggesting that image features that are unlikely to be age-relevant (e.g. fundus pigmentation, color distribution, or image brightness) may influence the model prediction. Among the female-specific associations, we identified two genes previously linked to retinal vessel tortuosity (*CAPN12* and *ACTN4*)^43,54,55^, in line with our observation that model attention maps highlighted blood vessels more prominently in females. Notably, despite prior reports of X-linked genes underlying retinal diseases and OCT-derived retinal thickness traits^56,57^, we did not observe genome-wide significant associations on the X chromosome for RAG. This suggests that RAG primarily reflects common, age-related features visible on color fundus imaging, rather than retinal thickness variation or structural changes characteristic of rare, early-onset retinopathies.

Saliency maps of age-prediction models consistently showed prominent activation in the macula and temporal half of the optic disc, while attention maps partially aligned by highlighting the optic disc and surrounding macular regions. Crucially, attention maps also emphasized the main vascular arches in females—particularly in older retinas—mirroring our latent-variable analysis, which showed strong correlations with vessel density, bifurcation count, and vessel diameter. We hypothesize that the number of visible small vessels, which typically decreases with age^58^, plays a role in model predictions. Interestingly, a recent study reported higher macular vascular density in males compared to females, with sex differences varying between foveal and parafoveal areas and across age groups^59^. This anatomical variation could help explain our observation that saliency maps focused on the fovea while attention maps emphasized the surrounding macular region. These findings suggest that the parafoveal vasculature may carry both age-relevant and sex-discriminative information, contributing to the model’s ability to capture distinct aging patterns between sexes.

Finally, our findings must be interpreted within the context of the study’s limitations. Although RETFound was pretrained on data with some ethnic diversity, both the UKB and RS cohorts analyzed here were predominantly composed of individuals of White European ancestry. As such, the generalizability of our results to other populations remains to be evaluated. Furthermore, both cohorts represent relatively healthy, aging populations. The applied image quality filter may also introduce a modest bias toward excluding individuals with disease, as certain ocular conditions can increase the likelihood of producing lower-quality images (see^43^ for a detailed quantification of this effect). Although individuals with diagnosed diseases remained represented in our study sample, the findings may not generalize to clinical populations with higher disease burden or atypical ocular presentations. Conversely, because the model is trained to predict chronological age, disease-related retinal features present in the training data may be incorporated into the learned age representation, creating an inherent tension in the interpretation of the RAG phenotype, which likely reflects a mixture of normative aging processes and disease-related changes. The limited number of incident dementia cases, particularly within sex-specific strata, restricted our ability to examine subtype-specific associations with sufficient statistical power. Part of the retinal age signal may reflect non-aging-related image characteristics, as suggested by associations with OCA2 and intensity-related image features, and therefore genetic loci associated with RAG should not be interpreted exclusively as reflecting aging-related biological pathways. Finally, although our interpretations are supported by converging evidence from genetic analyses, saliency and attention maps, correlations with interpretable image features, and clinical associations, the precise biological mechanisms underlying the age-related signals captured by the models remain incompletely understood and will require further investigation in longitudinal and mechanistic studies.

Overall, our study demonstrates that RAG represents a non-invasive retinal imaging-derived aging-associated phenotype linked to ocular, cardiovascular, metabolic, inflammatory, and cognitive dimensions of aging. Our findings highlight the potential of retinal imaging as an accessible tool for capturing systemic aging signals. Importantly, RAG is constructed to be orthogonal to chronological age, thereby capturing variation in retinal appearance beyond that explained by age and providing complementary information to conventional clinical risk factors. While training sex-specific models did not improve predictive accuracy, the distinct genetic and clinical correlates observed between females and males underscore the value of analyses stratified by sex and female menopausal status in aging research. Incorporating sex-specific perspectives will therefore be essential for refining aging biomarkers and guiding future studies of sex-specific aging processes.

## Methods

### Imaging data

This research was conducted using the UK Biobank (UKB) Resource under Application Number 90947. The UKB study was approved by the National Information Governance Board for Health and Social Care and the NHS North West Multi-centre Research Ethics Committee (MREC) as a Research Tissue Bank (REC reference: 16/NW/0274). We used color fundus images (CFIs) from UKB instances 0 and 1. These single-field, 45° color photographs, centered to capture the optic disc and macula, were acquired using a Topcon 3D OCT-1000 Mark II digital ophthalmic camera. To ensure image quality, we applied a quality control (QC) procedure based on the method described by Zekavat et al.^45^, excluding the lowest-quality 25% of images. Briefly, a quality score was assigned to each image based on a deep learning model trained to identify poor-quality scans, and images below the 25th percentile of the score distribution were removed. Given the overall distribution of image quality in the UKB dataset, this threshold primarily excluded images that were severely degraded (e.g. very dark or highly blurred), while retaining images of moderate quality, including those from participants with ocular or systemic diseases. This resulted in a final dataset of 130,360 images from 71,343 participants. We cropped black borders from all retained images^60^. Participants were between 39.2 and 79.1 years old (mean age = 57.7 ± 8.2 years). For the sex-specific analyses, we created age-matched cohorts of males and females, each comprising 32,918 individuals with a mean age of 57.9 ± 8.3 years and an age range of 40.1 to 75.8 years.

For the external validation of our models, we used data from the Rotterdam Study (RS), a prospective, population-based cohort study conducted in Ommoord, a district of the city of Rotterdam, The Netherlands^61^. It comprises four cohorts, initiated in 1989, 2000, 2006, and 2015, with a total of 17,931 participants. The RS has been approved by the Medical Ethics Committee of the Erasmus MC (registration number MEC 02.1015) and by the Dutch Ministry of Health, Welfare and Sport (Population Screening Act WBO, license number 1071272-159521-PG). The RS has been entered into the Netherlands National Trial Register (NTR; www.trialregister.nl) and into the WHO International Clinical Trials Registry Platform (ICTRP; https://apps.who.int/trialsearch/) under shared catalogue number NTR6831. The data used in this study were accessed under a data transfer agreement. All participants provided written informed consent to participate in the study and to have their information obtained from treating physicians. Participants undergo examinations approximately every five years, which include retinal imaging using various imaging modalities and camera systems across study rounds. Because discrepancies in results may arise from differences in imaging protocols and participant demographic characteristics, we aimed to select the subset of RS images most comparable to those used in the UKB analysis. For the model replication, CFIs acquired with the Topcon 3D OCT-2000 FA plus camera with a macula-centered field-of-view were used, as this modality was deemed most comparable to the UKB imaging while offering broad availability within the RS. In total, 37,771 fundus images were available from 7480 participants (14,912 eyes across 8813 visits). After filtering by age (40 <age ≤ 70 years, a range encompassing 97% of the UKB sample) and image quality (excluding the lowest 25%), 10,354 images from 4757 participants were retained for analysis (mean age = 58.0 ± 7.2 years). For the genetic analysis replication, we aimed to maximize sample size by selecting one assessment per participant according to the following rules: preferentially retaining Topcon 3D OCT-2000 FA macula-centered images when available, and otherwise using the closest available modality. When multiple assessments were present, we chose the visit with the highest average image quality. We applied the same age and quality filters. This resulted in a dataset of 11,368 participants (mean age = 60.6 ± 6.4 years) with 88,789 fundus images.

### RETFound fine-tuning

RETFound is a Vision Transformer-based foundation model pretrained on a large collection of unlabeled retinal images using self-supervised learning^25^. Originally designed for classification tasks, we adapted the architecture for regression to predict chronological age from CFIs. We employed a five-fold cross-validation framework, where participants were randomly divided into five non-overlapping groups. For each fold, we trained a separate model using 60% of participants for training, 20% for validation, and 20% for testing, with training, validation, and test sets rotating across folds. We used all available images, ensuring that, for a given fold, images from the same participant were assigned to the same set to avoid leakage between sets.

We resized images to 224 × 224 pixels. For data augmentation during training, we applied random crops and rescaling with a scale range of (0.8, 1.0) and aspect ratio range of (0.9, 1.1), along with random rotations up to 20°. We normalized all images using the ImageNet RGB mean and SD.

We trained models for 50 epochs, including 10 warm-up epochs. The learning rate followed a half-cycle cosine decay schedule: it increased linearly during the warm-up phase, then decreased following a cosine curve toward a minimum value. Specifically, the learning rate was initialized as 0.005×batch size/256, and decayed to a minimum of 1×10^−6^. We used mean absolute error (MAE) as the loss function. The batch size for training was 16. Additional training hyperparameters included a drop path rate of 0.2, weight decay of 0.05, and layer-wise learning rate decay of 0.65. With the exception of the adjustments required for regression, we maintained the original RETFound training configuration as closely as possible.

We repeated the same five-fold cross-validation procedure separately for female and male participants using sex-specific, age-matched samples. This resulted in a total of 15 models: five for the combined sample, five for females, and five for males. This cross-validation setup ensured that each image had an out-of-sample prediction, allowing for robust performance assessment across the entire dataset. We evaluated the model performance using the MAE, Pearson’s correlation (*r*), and coefficient of determination (R^2^) between the predictions and the chronological ages. For each participant at a given imaging visit, we averaged predicted ages across both eyes when available; otherwise we used the prediction from the single available eye.

For external validation in the RS, we applied the five combined-model weights to the entire RS dataset and averaged the predicted ages to produce a single ensemble prediction per image. To evaluate overall performance, we computed the MAE, *r*, and R^2^ between the ensemble predictions and the true ages. To assess the variability across individual models, we calculated the SD of the five model predictions for each image and reported the mean of these per-image SDs across the dataset. We then averaged predictions across eyes for each participant. We repeated this procedure using the sex-specific models on the corresponding female and male RS samples.

### Retinal age gap

Age-prediction models commonly exhibit regression-to-the-mean bias, whereby younger individuals tend to have their age overestimated and older individuals underestimated. This phenomenon induces a dependence between the resulting age gap and chronological age, which can introduce confounding in downstream analyses. To mitigate this bias, post hoc corrections are frequently applied in brain age studies but have been used less consistently in retinal age studies^26^. To correct for regression-to-the-mean bias in predicted age, we applied a post hoc adjustment to the eye-averaged predicted ages following the procedure described by Cole et al.^18^. Specifically, we fit a linear regression:

1 $$\hat{{{{\rm{Y}}}}}=\alpha Y+\beta+\varepsilon$$

where Ŷ is predicted age and Y chronological age, then corrected the predicted age as follows:

2 $${\hat{{{{\rm{Y}}}}}}_{{corr}}=\frac{\hat{{{{\rm{Y}}}}}-\beta }{\alpha }$$

We then defined the retinal age gap (RAG) as:

3 $${RAG}={\hat{{{{\rm{Y}}}}}}_{{corr}}-Y$$

All subsequent analyses were performed using this corrected RAG measure. To assess sex differences, we compared mean RAG values derived from the combined-sample model between females and males using independent-sample *t*-tests and quantified the effect size with Cohen’s *d*. To explore potential hormonal influences, we also compared RAG across female subgroups defined by menopausal status (UKB only, not available for RS) and by age (<55 vs ≥55 years) using the same approach. We performed this analysis in both the UKB and RS datasets, while subsequent disease associations were conducted using UKB data only.

### Association with health and lifestyle factors

To investigate the relationship between RAG and various health outcomes, we conducted a cross-sectional analysis using data from the UKB. For participants with imaging data available at multiple time points, we used the first available instance.

Continuous outcomes included body mass index (BMI; data-field (DF) 21001), waist-to-hip ratio (WHR) defined as waist circumference (DF 48) divided by hip circumference (DF 49), systolic blood pressure (SBP; DF 4080 averaged across measurements), diastolic blood pressure (DBP; DF 4079 averaged across measurements), log-transformed pack-years of smoking (DF 20161), glycated hemoglobin (HbA1c; DF 30750), glucose (DF 30740), white blood cell (leukocyte) count (DF 30000), C-reactive protein (CRP; 30710), neutrophil (DF 30140) to lymphocyte (DF 30120) ratio (NLR), insulin-like growth factor 1 (IGF-1; DF 30770), low-density lipoprotein (LDL) cholesterol (DF 30780), high-density lipoprotein (HDL) cholesterol (DF 30760), triglycerides (DF 30870), total cholesterol (DF 30690), VO₂max cardiorespiratory fitness estimate (DF 30038), sleep duration (DF 1160), reasoning score (also called fluid intelligence score; DF 20016), reaction time (DF 20023), and log-transformed telomere length (DF 22192). For participants who took antihypertensive medication, we added 10 mmHg to SBP and 5 mmHg to DBP values. Similarly, for participants who took cholesterol-lowering medication, we added 1.4 mmol/L to LDL, 0.4 mmol/L to triglycerides, 1.6 mmol/L to total cholesterol, and subtracted 0.1 mmol/L from HDL. Sex-specific associations additionally included testosterone (DF 30850) for males, and age at menopause (DF 3581) for females.

Binary outcomes included the use of diabetes, cholesterol, or BP medication (DFs 6153 and 6177), diabetes—defined as glucose >= 11.1 and/or HbA1c >= 48 and/or self-reported diagnosis by a doctor (based on DFs 2443 and 2976) and/or diabetes medication use—, hypertension—SBP > 140 and/or DBP > 90 and/or BP medication use—, dyslipidemia—cholesterol >= 5 and/or HDL <= 1 and/or LDL >= 4.1 and/or triglycerides >= 2.2 and/or cholesterol medication use—, eye diseases (DF 6148) including cataract, glaucoma, and macular degeneration, cataract surgery (DF 5324), cancer history (DF 2453), metabolic syndrome—defined according to the International Diabetes Federation consensus^62^ as having three or more of the following: large waist circumference (>= 80 cm for females or >= 94 cm for males), triglycerides > 1.7 or cholesterol medication, low HDL ( < 1.29 for females or < 1.03 for males), high BP (SBP >= 130 and/or DBP >= 85 and/or BP medication), and high glucose which we substituted with the above definition of diabetes since fasting glucose was not available—, smoking status (current/previous vs. never; DF 20116), alcohol intake (occasional/regular vs. never; DF 1558), having children (binarized from DFs 2734 and 2405), visual memory (>= median in the pairs matching task; DF 399), and prospective memory (correct recall in first attempt; DF 20018). Additionally for females, we included the menopausal status (DF 2724), contraceptive pill (DF 6153), and hormone replacement therapy (HRT; DF 6153).

All models controlled for age (derived from DFs 34, 52, and 53), age-squared, sex (DF 31), height (DF 50), height-squared, self-reported ethnic background (DF 21000, binarized as White/British vs. other given the low number of non-White participants), the first 10 genetic principal components (PCs; DF 22009), educational attainment (DF 6138)—categorized as low (“CSEs or equivalent”, “NVQ or HND or HNC or equivalent”, “Other professional qualifications eg: nursing, teaching”, or “None of the above”), medium (“A levels/AS levels or equivalent”, “O levels/GCSEs or equivalent”), or high (“College or University degree”)^63^—, household income (DF 738)—categorized as low (< 18,000 £), medium (between 18,000 and 51,999 £), or high (>= 52,000 £)—, and Townsend Deprivation Index (DF 22189). We imputed missing covariate data using the group mean (for continuous covariates) or mode (for categorical covariates).

We standardized (*z*-scored) continuous outcomes and covariates, and excluded values that lay beyond 4 SD from the group mean. We applied linear regression models for standardized continuous outcomes and logistic regression for binary traits, with RAG entered as a standardized predictor. Additionally, using RAG derived from the sex-specific models, we conducted sex-stratified analyses to explore differential associations, adjusting for the same covariates as in the combined-sample analyses except sex. We compared female- and male-specific effects using a *z*-test. In females, we further repeated analyses stratified by menopausal status and by age group (<55 vs ≥55 years) to examine potential differences across life stages.

To evaluate whether RAG contributed additional explanatory power beyond other covariates, we performed likelihood ratio tests comparing nested models with and without RAG. We corrected *p*-values for multiple testing (122 tests) using false discovery rate (FDR)^64^ with an alpha threshold of 0.05.

### Survival analysis

To assess the prospective associations between RAG and major health outcomes, we conducted Cox proportional hazards regression analyses using follow-up data spanning approximately 15 years from the UKB. We performed time-to-event analyses for all-cause mortality (DF 40000) and incident cases of chronic obstructive pulmonary disease (DFs 42016, 131486, 131488, 131490, and 131492), cancer (DF 40005), dementia (DFs 42018, 130836, 130838, 130840, and 130842), and cardiovascular disease (CVD), which encompassed stroke (DFs 42006, 131366, 131362, and 131364), thrombosis (DFs 131308, 131388, and 131400), myocardial infarction (DFs 131298, 131300, 131302, and 42000), ischemic heart disease (DFs 131296, 131304, and 131306), and non-ischemic cardiomyopathy (DFs 131338, 131340, 131288, and 131292). We adjusted models for age, sex (combined analyses only), height, ethnicity, education, income, Townsend, SBP, HDL, total cholesterol, smoking status, BMI, diabetes, BP medication, and previous events from the same category (for CVDs and cancer only). Thus, compared with the cross-sectional analyses, the Cox models additionally included established clinical risk factors and prior disease history to assess the predictive value of RAG beyond conventional risk factors. Because several outcomes, particularly in sex-stratified analyses, had relatively limited numbers of incident events, we used a more parsimonious covariate specification in the Cox models to limit model complexity and potential overfitting^65^, and therefore did not include squared terms for age and height or genetic PCs. We conducted analyses in the full sample using RAG derived from the combined model, and sex-stratified analyses using RAG derived from the corresponding sex-specific models. We additionally stratified females by menopausal status and age group (<55 vs ≥55 years) to examine potential life-stage-specific associations. To assess the incremental contribution of RAG, we performed likelihood ratio tests comparing nested Cox models with and without RAG. We corrected *p*-values for multiple testing (30 tests) using FDR^64^ with an alpha threshold of 0.05. We also evaluated changes in model discrimination by comparing Harrell’s C-index between the nested models using bootstrap resampling (200 iterations).

To quantify the relative importance of each predictor (including covariates), we conducted a leave-one-out analysis in which each variable was removed from the multivariable model one at a time and the resulting reduction in model fit was calculated from the change in log-likelihood. The corresponding chi-square statistic was computed for each variable and expressed as a percentage of the total chi-square across predictors, providing an estimate of each variable’s relative contribution to the model.

### Genome-wide association study

We performed a genome-wide association study (GWAS) of RAG using Regenie^66^, which accounts for relatedness and population structure via a two-step ridge regression approach. Before conducting the analyses, we applied filters for minor allele frequency (MAF ≥ 0.01), minor allele count (MAC ≥ 100), genotype missingness (≤ 0.1), and Hardy–Weinberg equilibrium (HWE, p ≥ 1×10⁻¹⁵). Because quality control of the X chromosome remains debated given its biological specificities, we implemented additional steps^67^. We handled the X chromosome separately in each sex, using a relaxed missingness threshold for males (≤ 0.2) and excluding all ampliconic regions. We did not apply HWE filtering to the X chromosome, as the HWE test does not accommodate hemizygous males and may otherwise eliminate valid variants. Prior to the analysis, we applied a rank-inverse normal transformation to the RAG phenotype. The covariates included in the GWAS were age, sex (DF 31)—excluding cases of aneuploidy (DF 22019) or when genetic sex (DF 22001) did not match recorded or self-reported sex (DF 31)—, age-squared, age-by-sex, age-squared-by-sex, height, height-squared, instance, assessment centre (DF 21003), genotype measurement batch (DF 22000), spherical power (DFs 5084 and 5085), spherical power-squared, cylindrical power (DFs 5086 and 5087), cylindrical power-squared, and the first 20 genetic PCs. For sex-specific GWAS analyses, we used RAG derived from the corresponding sex-specific models and applied the same covariates excluding sex. For all SNP-level analyses, we applied the genome-wide significance threshold, i.e. 5×10^−8^.

We computed gene-level association scores using PascalX^29^, which aggregates single nucleotide polymorphism (SNP)-level statistics into gene scores using the approximate “saddle” method. We scored protein-coding genes, including all SNPs with MAF ≥ 0.05, located within a 50 kb window around each gene. We used the UK10K reference panel^68^ for gene scoring on the autosomes while we built the X chromosomal panel independently for females and males using 5000 unrelated white individuals per sex from the UKB^67^. For the gene-level analyses, we corrected for multiple testing by setting the alpha threshold to 0.05 divided by the number of tested genes, i.e. 2.61×10^−6^ in the combined sample and 2.51×10^−6^ in the sex-specific samples.

We estimated SNP-based heritability (h^2^_SNP_) of RAG using the genomic-relatedness-based restricted maximum-likelihood (GREML) method implemented in the GCTA (Genome-wide complex trait analysis) software package^69^. We fitted a genetic relationship matrix (GRM) constructed from common (MAF > 0.01) and directly genotyped variants to estimate the proportion of variance explained in RAG by all measured SNPs.

For the replication analysis, because imputation and QC were conducted independently in the four RS sub-cohorts, we first ran GWAS within each cohort and subsequently combined the results in a meta-analysis. Genotypes were imputed using the Haplotype Reference Consortium reference panel version 1.1^70^. For the individual GWAS, we applied linear regression models in PLINK 2.0^71^, adjusting for age, sex, age-squared, age-by-sex, age-squared-by-sex, height, height-squared, spherical power, spherical power-squared, cylindrical power, cylindrical power-squared, and the first 10 genetic PCs. We then meta-analyzed results across cohorts using an inverse-variance weighted fixed-effects approach implemented in METAL^72^. We derived association *p*-values from the corresponding *z*-statistics. We computed gene scores using PascalX^29^ with the parameters described above. To assess replication of genetic findings, we adopted a candidate approach, applying FDR correction with a threshold 0.05 to *p*-values from SNPs, respectively genes, that were genome-wide significant in the UKB. We also computed Pearson’s correlation between SNPs effect sizes (*β*) in the UKB and in the RS to assess consistency of results regardless of significance.

### Saliency and attention maps

To interpret the internal representations learned by RETFound and visualize the model’s implicit focus during prediction, we applied two distinct methods.

First, we generated saliency maps using RELPROP^30^, a method that applies layer-wise relevance propagation (LRP) tailored for Transformer-based architectures, as used in the original RETFound publication^25^. RELPROP reveals model focus by integrating relevance scores with gradient information and propagating relevance backward through the whole network while preserving the total relevance across layers, creating more accurate attribution than conventional attention visualization alone. To accurately capture RETFound’s prediction process, we adapted the RELPROP method to handle the global pooling architecture by distributing relevance equally across all non-CLS tokens, ensuring proper attribution for the regression pathways. Furthermore, we generated group-average saliency visualizations that highlight image regions consistently attended to by the model across a large set of input images, revealing which anatomical structures drive the model’s decision-making process at the population level. This approach is meaningful in our context, as we restricted the analysis to left eye images (right eye saliency maps are shown in Supplementary Fig. 10), ensuring that key anatomical features such as the optic disc and macula were consistently located across individuals.

Second, we extracted attention maps from the 24 attention blocks of the Vision Transformer, and computed attention rollout^73^. Specifically, we captured the spatial attention scores of the class token directed towards the patch tokens (CLS→patch attention), a commonly used proxy for identifying important image regions in Transformer-based models^74^. We implemented a forward hook to extract the softmax-normalized attention weights from each block. These weights represent the flow of information within the Transformer, indicating how much the CLS token relies on other patches, and form 14×14 spatial attention maps for each of the 16 attention heads. We then averaged the attention across all heads and aggregated across all blocks to obtain a single attention map per image^73^. Finally, similarly to saliency maps, we averaged attention maps across the cohorts to produce population-level visualizations of the regions most consistently attended to by the model.

Saliency maps and attention maps represent different but complementary approaches to interpreting Transformer-based models. Saliency maps operate in a backward direction, tracing the model’s decision back through the network using relevance scores combined with gradients. This yields causal attributions by accounting for non-linear interactions across all layers and attention heads. In contrast, attention maps are obtained in the forward pass, using the softmax-normalized weights from the attention layers. These reflect the model’s internal information routing (i.e., how much each input token is attended to by the CLS token), but do not indicate actual influence on the output, making them associative rather than causal. Because saliency maps trace decision-making and attention maps reveal focus patterns, differences between them can be informative. For example, the model might consistently attend to certain patches for context, while relying on different regions to make the final prediction.

### Linking latent representations to interpretable image features

To investigate which interpretable image features the models may rely on to predict age, we examined Pearson’s correlations between the 1024 latent variables (LVs), derived from the encoder portion of the models, and a comprehensive set of handcrafted retinal features. Specifically, we computed correlations between each LV and 24 image-derived variables: mean image intensity defined as the mean of the red, green, and blue channels, SD of image intensity, mean and SD of the red, green, and blue channels separately, optic disc area, central retinal arterial equivalent, central retinal venous equivalent, median and SD of artery and vein diameters, artery and vein tortuosity, artery and vein bifurcation counts, artery and vein temporal angles, artery and vein vascular density, and pigmentation score. All vascular features were extracted using the VascX software^75^. For the optic disc segmentation, we annotated a random set of 100 CFIs and used them to re-train a publicly available U-Net model^76^. We computed the pigmentation score using the pipeline published in^77^, which was developed to decouple traditional demographic variables from clinical imaging characteristics and has been previously validated on the UKB data. We computed correlations separately for each of the 15 trained models (across five cross-validation folds and three sex groups). Additionally, for the 24 image features, we computed correlations with the chronological age and RAG.

### Reporting summary

Further information on research design is available in the Nature Portfolio Reporting Summary linked to this article.

## Supplementary information

Supplementary Information

Description of Additional Supplementary Files

Supplementary Data 1-13

Reporting Summary

Transparent Peer Review file

## Source data

Source Data

## Data Availability

The GWAS summary statistics generated in this study are available in the GWAS Catalog (https://www.ebi.ac.uk/gwas/) under accession codes GCST90992810, GCST90992811, GCST90992812, and GCST90992813. UK Biobank data are available upon successful application (https://www.ukbiobank.ac.uk/enable-your-research/apply-for-access). Rotterdam Study data can be obtained upon request. Requests should be directed towards the management team of the Rotterdam Study (datamanagement.ergo@erasmusmc.nl), which has a protocol for approving data requests. Because of restrictions based on privacy regulations and informed consent of the participants, data cannot be made freely available in a public repository. Source data are provided with this paper.

## Code availability

RETFound code is publicly available at https://github.com/rmaphoh/RETFound. The code used to generate the presented results is available at https://github.com/ot710/retinal-age and is archived at Zenodo^78^.

## References

[1] Mathur, A., Taurin, S. & Alshammary, S. New insights into methods to measure biological age: a literature review. *Front. Aging* **5**, 1395649 (2024). 10.3389/fragi.2024.1395649 PMC11688636 39743988

[2] Haugg, F. et al. Imaging biomarkers of ageing: a review of artificial intelligence-based approaches for age estimation. *Lancet Healthy Longev*. 100728 (2025) 10.1016/j.lanhl.2025.100728. 40690918

[3] Poplin, R. et al. Prediction of cardiovascular risk factors from retinal fundus photographs via deep learning. *Nat. Biomed. Eng.* **2**, 158–164 (2018). 10.1038/s41551-018-0195-0 31015713

[4] Kim, Y. D. et al. Effects of hypertension, diabetes, and smoking on age and sex prediction from retinal fundus images. *Sci. Rep.* **10**, 4623 (2020). 10.1038/s41598-020-61519-9 PMC7067849 32165702

[5] Gerrits, N. et al. Age and sex affect deep learning prediction of cardiometabolic risk factors from retinal images. *Sci. Rep.* **10**, 9432 (2020). 10.1038/s41598-020-65794-4 PMC7287116 32523046

[6] Rim, T. H. et al. Prediction of systemic biomarkers from retinal photographs: development and validation of deep-learning algorithms. *Lancet Digit. Health* **2**, e526–e536 (2020). 10.1016/S2589-7500(20)30216-8 33328047

[7] Zhu, Z. et al. Retinal age gap as a predictive biomarker for mortality risk. *Br. J. Ophthalmol.* **107**, 547–554 (2023). 10.1136/bjophthalmol-2021-319807 35042683

[8] Peng, Q. et al. Predictive potential of retina-based biological age in assessing chronic obstructive pulmonary disease risk. *Clin. Exp. Ophthalmol.* 10.1111/ceo.14501 (2025). 39916391

[9] Ahadi, S. et al. Longitudinal fundus imaging and its genome-wide association analysis provide evidence for a human retinal aging clock. *Elife* **12**, (2023). 10.7554/eLife.82364 PMC10110236 36975205

[10] Le Goallec, A., Diai, S., Collin, S., Vincent, T. & Patel, C. J. Deep learning of fundus and optical coherence tomography images enables identification of diverse genetic and environmental factors associated with eye aging. *medRxiv* 10.1101/2021.06.24.21259471 (2021).

[11] Wang, J. et al. Accurate estimation of biological age and its application in disease prediction using a multimodal image Transformer system. *Proc. Natl. Acad. Sci. Usa.* **121**, e2308812120 (2024). 10.1073/pnas.2308812120 PMC10801873 38190540

[12] Nusinovici, S. et al. Retinal photograph-based deep learning predicts biological age, and stratifies morbidity and mortality risk. *Age Ageing* **51**, (2022). 10.1093/ageing/afac065 PMC8973000 35363255

[13] Grimbly, M. J. et al. Estimating biological age from retinal imaging: a scoping review. *BMJ Open Ophthalmol.* **9**, e001794 (2024). 10.1136/bmjophth-2024-001794 PMC11344507 39181547

[14] Zhu, Z. et al. Association of retinal age gap with arterial stiffness and incident cardiovascular disease. *Stroke* **53**, 3320–3328 (2022). 10.1161/STROKEAHA.122.038809 35880520

[15] Hu, W. et al. Retinal age gap as a predictive biomarker of future risk of Parkinson’s disease. *Age Ageing* **51**, (2022). 10.1093/ageing/afac062 PMC8966015 35352798

[16] Nusinovici, S. et al. Application of a deep-learning marker for morbidity and mortality prediction derived from retinal photographs: a cohort development and validation study. *Lancet Healthy Longev.* **5**, 100593 (2024). 10.1016/S2666-7568(24)00089-8 PMC11788570 39362226

[17] Goyal, M. S. et al. Persistent metabolic youth in the aging female brain. *Proc. Natl. Acad. Sci. Usa.* **116**, 3251–3255 (2019). 10.1073/pnas.1815917116 PMC6386682 30718410

[18] Cole, J. H. et al. Brain age predicts mortality. *Mol. Psychiatry* **23**, 1385–1392 (2018). 10.1038/mp.2017.62 PMC5984097 28439103

[19] Subramaniapillai, S. et al. Sex-dependent effects of cardiometabolic health and APOE4 on brain age: A longitudinal cohort study. *Neurology* **103**, e209744 (2024). 10.1212/WNL.0000000000209744 PMC11379441 39173100

[20] Phyo, A. Z. Z. et al. Sex differences in biological aging and the association with clinical measures in older adults. *GeroScience* **46**, 1775–1788 (2024). 10.1007/s11357-023-00941-z PMC10828143 37747619

[21] Smith, S. M., Vidaurre, D., Alfaro-Almagro, F., Nichols, T. E. & Miller, K. L. Estimation of brain age delta from brain imaging. *Neuroimage* **200**, 528–539 (2019). 10.1016/j.neuroimage.2019.06.017 PMC6711452 31201988

[22] Sanford, N. et al. Sex differences in predictors and regional patterns of brain age gap estimates. *Hum. Brain Mapp.* **43**, 4689–4698 (2022). 10.1002/hbm.25983 PMC9491279 35790053

[23] Takahashi, S. et al. Comparison of vision transformers and convolutional neural networks in medical image analysis: A systematic review. *J. Med. Syst.* **48**, 84 (2024). 10.1007/s10916-024-02105-8 PMC11393140 39264388

[24] Isensee, F. et al. NnU-net revisited: A call for rigorous validation in 3D medical image segmentation. in *Lecture Notes in Computer Science* 488–498 (Springer Nature Switzerland, Cham, 2024). 10.1007/978-3-031-72114-4_47.

[25] Zhou, Y. et al. A foundation model for generalizable disease detection from retinal images. *Nature* **622**, 156–163 (2023). 10.1038/s41586-023-06555-x PMC10550819 37704728

[26] de Lange, A.-M. G. & Cole, J. H. Commentary: Correction procedures in brain-age prediction. *NeuroImage Clin.* **26**, 102229 (2020). 10.1016/j.nicl.2020.102229 PMC7049655 32120292

[27] Nangia, V. et al. Body height and ocular dimensions in the adult population in rural Central India. The Central India Eye and Medical Study. *Arbeitsphysiologie* **248**, 1657–1666 (2010). 10.1007/s00417-010-1448-0 20652306

[28] Zoccali, C. & Tripepi, G. The conservativeness of standard C statistics in the prediction of clinical events. *Eur. J. Clin. Invest.* **56**, e70150 (2026). 10.1111/eci.70150 41243705

[29] Krefl, D., Brandulas Cammarata, A. & Bergmann, S. PascalX: a Python library for GWAS gene and pathway enrichment tests. *Bioinformatics* **39**, btad296 (2023). 10.1093/bioinformatics/btad296 PMC10185402 37137228

[30] Chefer, H., Gur, S. & Wolf, L. Transformer Interpretability Beyond Attention Visualization. in *2021 IEEE/CVF Conference on Computer Vision and Pattern Recognition (CVPR)* (IEEE, 2021). 10.1109/cvpr46437.2021.00084.

[31] Fasero, M. & Coronado, P. J. Cardiovascular disease risk in women with menopause. *J. Clin. Med.* **14**, 3663 (2025). 10.3390/jcm14113663 PMC12156203 40507425

[32] Noubiap, J. J. et al. Worldwide trends in metabolic syndrome from 2000 to 2023: a systematic review and modelling analysis. *Nat. Commun.* **17**, 573 (2025). 10.1038/s41467-025-67268-5 PMC12808104 41350289

[33] Davis, S. R., Pinkerton, J., Santoro, N. & Simoncini, T. Menopause—Biology, consequences, supportive care, and therapeutic options. *Cell* **186**, 4038–4058 (2023). 10.1016/j.cell.2023.08.016 37678251

[34] Kottilil, S. & Mathur, P. The influence of inflammation on cardiovascular disease in women. *Front. Glob. Women’s. Health* **3**, 979708 (2022). 10.3389/fgwh.2022.979708 PMC9592850 36304737

[35] Rubin, J. B. et al. Sex differences in cancer mechanisms. *Biol. Sex. Differ.* **11**, 17 (2020). 10.1186/s13293-020-00291-x PMC7161126 32295632

[36] Rasmussen, I. J., Rasmussen, K., Nordestgaard, B., Tybjærg-Hansen, A. & Frikke-Schmidt, R. Cardiovascular risk factors and risk of vascular-related dementia in high-risk and low-risk prospective cohort studies. *Atherosclerosis* **407**, 120319 (2024).

[37] Huang, Y. et al. Genomic determinants of biological age estimated by deep learning applied to retinal images. *GeroScience* **47**, 2613–2629 (2025). 10.1007/s11357-024-01481-w PMC11979078 39775603

[38] He, W. et al. Association of novel loci with keratoconus susceptibility in a multitrait genome-wide association study of the UK Biobank database and Canadian Longitudinal Study on Aging. *JAMA Ophthalmol.* **140**, 568–576 (2022). 10.1001/jamaophthalmol.2022.0891 PMC9026225 35446358

[39] Okbay, A. et al. Polygenic prediction of educational attainment within and between families from genome-wide association analyses in 3 million individuals. *Nat. Genet.* **54**, 437–449 (2022). 10.1038/s41588-022-01016-z PMC9005349 35361970

[40] Liyanage, U. E. et al. Combined analysis of keratinocyte cancers identifies novel genome-wide loci. *Hum. Mol. Genet.* **28**, 3148–3160 (2019). 10.1093/hmg/ddz121 PMC6737293 31174203

[41] Verma, A. et al. Diversity and scale: Genetic architecture of 2068 traits in the VA Million Veteran Program. *Science* **385**, eadj1182 (2024). 10.1126/science.adj1182 PMC12857194 39024449

[42] Lucarini, N., Napolioni, V., Magrini, A. & Gloria, F. The Effect of ACP(1)-ADA(1) Genetic Interaction on Human Life Span. *Hum. Biol.* **84**, 725–733 (2012). 10.3378/027.084.0606 23959645

[43] Ortín Vela, S. et al. Phenotypic and genetic characteristics of retinal vascular parameters and their association with diseases. *Nat. Commun.* **15**, 9593 (2024). 10.1038/s41467-024-52334-1 PMC11542103 39505872

[44] Choi, G. S., Min, H. S. & Cha, J. J. SH3YL1 protein as a novel biomarker for diabetic nephropathy in type 2 diabetes mellitus. Nutrition, metabolism, and cardiovascular diseases. *NMCD* **31**, 498–505 (2021). 10.1016/j.numecd.2020.09.024 33223406

[45] Zekavat, S. M. et al. Deep learning of the retina enables phenome- and genome-wide analyses of the microvasculature. *Circulation* **145**, 134–150 (2022). 10.1161/CIRCULATIONAHA.121.057709 PMC8746912 34743558

[46] Woodling, N. S. et al. The neuronal receptor tyrosine kinase Alk is a target for longevity. *Aging Cell* **19**, e13137 (2020). 10.1111/acel.13137 PMC7253064 32291952

[47] Islam, M. R. The International Headache Genetics Consortium Ihgc & Nyholt, D. R. Genetic overlap analysis identifies a shared etiology between migraine and headache with type 2 diabetes. *Genes (Basel)* **13**, 1845 (2022). 10.3390/genes13101845 PMC9601333 36292730

[48] Tang, X.-J., Shentu, X.-C., Tang, Y.-L., Ping, X.-Y. & Yu, X.-N. The impact of GJA3 SNPs on susceptibility to age-related cataract. *Int. J. Ophthalmol.* **12**, 1008–1011 (2019). 10.18240/ijo.2019.06.21 PMC6580199 31236361

[49] Fritsche, L. G. et al. Age-related macular degeneration is associated with an unstable ARMS2 (LOC387715) mRNA. *Nat. Genet.* **40**, 892–896 (2008). 10.1038/ng.170 18511946

[50] Choquet, H. et al. A large multiethnic GWAS meta-analysis of cataract identifies new risk loci and sex-specific effects. *Nat. Commun.* **12**, 3595 (2021). 10.1038/s41467-021-23873-8 PMC8203611 34127677

[51] Banumathy, G. et al. Human UBN1 is an ortholog of yeast Hpc2p and has an essential role in the HIRA/ASF1a chromatin-remodeling pathway in senescent cells. *Mol. Cell. Biol.* **29**, 758–770 (2009). 10.1128/MCB.01047-08 PMC2630694 19029251

[52] Blue, E. E. et al. Non-coding variants in MYH11, FZD3, and SORCS3 are associated with dementia in women. *Alzheimers Dement.* **17**, 215–225 (2021). 10.1002/alz.12181 PMC7920533 32966694

[53] Wang, F. et al. Comprehensive analysis of PTPN gene family revealing PTPN7 as a novel biomarker for immuno-hot tumors in breast cancer. *Front. Genet.* **13**, 981603 (2022). 10.3389/fgene.2022.981603 PMC9548886 36226189

[54] Tomasoni, M. et al. Genome-wide Association Studies of retinal vessel tortuosity identify numerous novel loci revealing genes and pathways associated with ocular and cardiometabolic diseases. *Ophthalmol. Sci.* **3**, 100288 (2023). 10.1016/j.xops.2023.100288 PMC10149284 37131961

[55] Jiang, X. et al. GWAS on retinal vasculometry phenotypes. *PLoS Genet.* **19**, e1010583 (2023). 10.1371/journal.pgen.1010583 PMC9910644 36757925

[56] De Silva, S. R. et al. The X-linked retinopathies: Physiological insights, pathogenic mechanisms, phenotypic features and novel therapies. *Prog. Retin. Eye Res.* **82**, 100898 (2021). 10.1016/j.preteyeres.2020.100898 32860923

[57] Jackson, V. E. et al. Multi-omic spatial effects on high-resolution AI-derived retinal thickness. *Nat. Commun.* **16**, 1317 (2025). 10.1038/s41467-024-55635-7 PMC11794613 39904976

[58] Wei, Y. et al. Age-related alterations in the retinal microvasculature, microcirculation, and microstructure. *Invest. Ophthalmol. Vis. Sci.* **58**, 3804–3817 (2017). 10.1167/iovs.17-21460 PMC5527847 28744554

[59] Ghassemi, F., Karimi, M., Salari, F. & Bayat, K. Exploring the impact of age and gender on retinal and choroidal thickness and vascular densities: a comprehensive analysis. *Int. J. Retin. Vitreous* **11**, 38 (2025). 10.1186/s40942-024-00619-4 PMC11959737 40165292

[60] Zhou, Y. et al. AutoMorph: Automated retinal vascular morphology quantification via a deep learning pipeline. *Transl. Vis. Sci. Technol.* **11**, 12 (2022). 10.1167/tvst.11.7.12 PMC9290317 35833885

[61] Ikram, M. A. et al. Objectives, design and main findings until 2020 from the Rotterdam Study. *Eur. J. Epidemiol.* **35**, 483–517 (2020). 10.1007/s10654-020-00640-5 PMC7250962 32367290

[62] Alberti, K. G. M. M., Zimmet, P. & Shaw, J. Metabolic syndrome—a new world-wide definition. A Consensus Statement from the International Diabetes Federation. *Diabet. Med.* **23**, 469–480 (2006). 10.1111/j.1464-5491.2006.01858.x 16681555

[63] Chadeau-Hyam, M. et al. Education, biological ageing, all-cause and cause-specific mortality and morbidity: UK biobank cohort study. *EClinicalMedicine* **29-30**, 100658 (2020). 10.1016/j.eclinm.2020.100658 PMC7788440 33437953

[64] Benjamini, Y. & Hochberg, Y. Controlling the False Discovery Rate: A Practical and Powerful Approach to Multiple Testing. *J. R. Stat. Soc. Ser. B Stat. Methodol.* **57**, 289–300 (1995).

[65] Concato, J., Peduzzi, P., Holford, T. R. & Feinstein, A. R. Importance of events per independent variable in proportional hazards analysis. I. Background, goals, and general strategy. *J. Clin. Epidemiol.* **48**, 1495–1501 (1995). 10.1016/0895-4356(95)00510-2 8543963

[66] Mbatchou, J. et al. Computationally efficient whole-genome regression for quantitative and binary traits. *Nat. Genet.* **53**, 1097–1103 (2021). 10.1038/s41588-021-00870-7 34017140

[67] Böttger, L. et al. Sex-specific disease association and genetic architecture of retinal vascular traits. *bioRxiv* 10.1101/2025.07.16.665150 (2025).

[68] Huang, J. et al. Improved imputation of low-frequency and rare variants using the UK10K haplotype reference panel. *Nat. Commun.* **6**, 8111 (2015). 10.1038/ncomms9111 PMC4579394 26368830

[69] Yang, J., Lee, S. H., Goddard, M. E. & Visscher, P. M. GCTA: a tool for genome-wide complex trait analysis. *Am. J. Hum. Genet* **88**, 76–82 (2011). 10.1016/j.ajhg.2010.11.011 PMC3014363 21167468

[70] the Haplotype Reference Consortium. A reference panel of 64,976 haplotypes for genotype imputation. *Nat. Genet*. **48**, 1279–1283 (2016). 10.1038/ng.3643 PMC5388176 27548312

[71] Chang, C. C. et al. Second-generation PLINK: rising to the challenge of larger and richer datasets. *Gigascience* **4**, 7 (2015). 10.1186/s13742-015-0047-8 PMC4342193 25722852

[72] Willer, C. J., Li, Y. & Abecasis, G. R. METAL: fast and efficient meta-analysis of genomewide association scans. *Bioinformatics* **26**, 2190–2191 (2010). 10.1093/bioinformatics/btq340 PMC2922887 20616382

[73] Kashefi, R., Barekatain, L., Sabokrou, M. & Aghaeipoor, F. Explainability of vision transformers: a comprehensive review and new perspectives. *Multimed. Tools Appl*. **85**, (2026).

[74] Caron, M. et al. Emerging properties in self-supervised vision transformers. in *2021 IEEE/CVF International Conference on Computer Vision (ICCV)* (IEEE, 2021). 10.1109/iccv48922.2021.00951.

[75] Vargas Quiros, J., Liefers, B., van Garderen, K. A., Vermeulen, J. P. & Klaver, C. VascX models: Deep ensembles for retinal vascular analysis from color fundus images. *Transl. Vis. Sci. Technol.* **14**, 19 (2025). 10.1167/tvst.14.7.19 PMC12306690 40699175

[76] Sevastopolsky, A. Optic disc and cup segmentation methods for glaucoma detection with modification of U-Net convolutional neural network. *Pattern Recognit. Image Anal.* **27**, 618–624 (2017).

[77] Rajesh, A. E. et al. Machine learning derived retinal pigment score from ophthalmic imaging shows ethnicity is not biology. *Nat. Commun.* **16**, 60 (2025). 10.1038/s41467-024-55198-7 PMC11696055 39746957

[78] Trofimova, O. & Liefers, B. *Deep learning aging marker from retinal images unveils sex-specific clinical and genetic signatures*. (Zenodo, 2026). 10.5281/ZENODO.21877477.42786167

