# Supplementary material for: Deep learning aging marker from retinal images unveils sex-specific clinical and genetic signatures
Source: Nat Commun. 2026 Aug 26;17:10185. doi: 10.1038/s41467-026-77102-1 (PMC13612558; doi:10.1038/s41467-026-77102-1)
Supplement: Supplementary file 1 [file 41467_2026_77102_MOESM1_ESM.pdf]

Deep learning aging marker from retinal images unveils sex-specific clinical and genetic signatures – Supplementary Figures

a

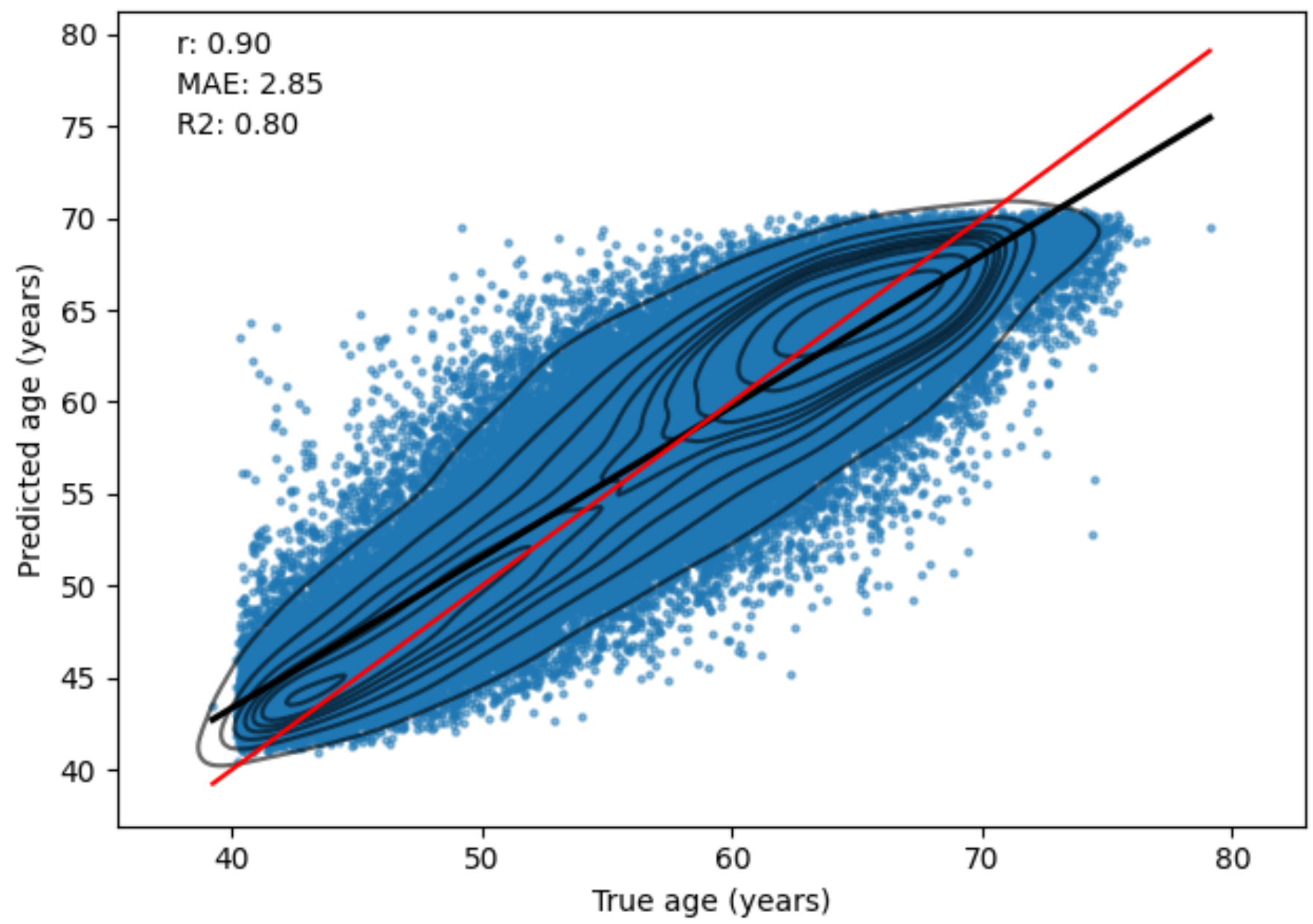

b

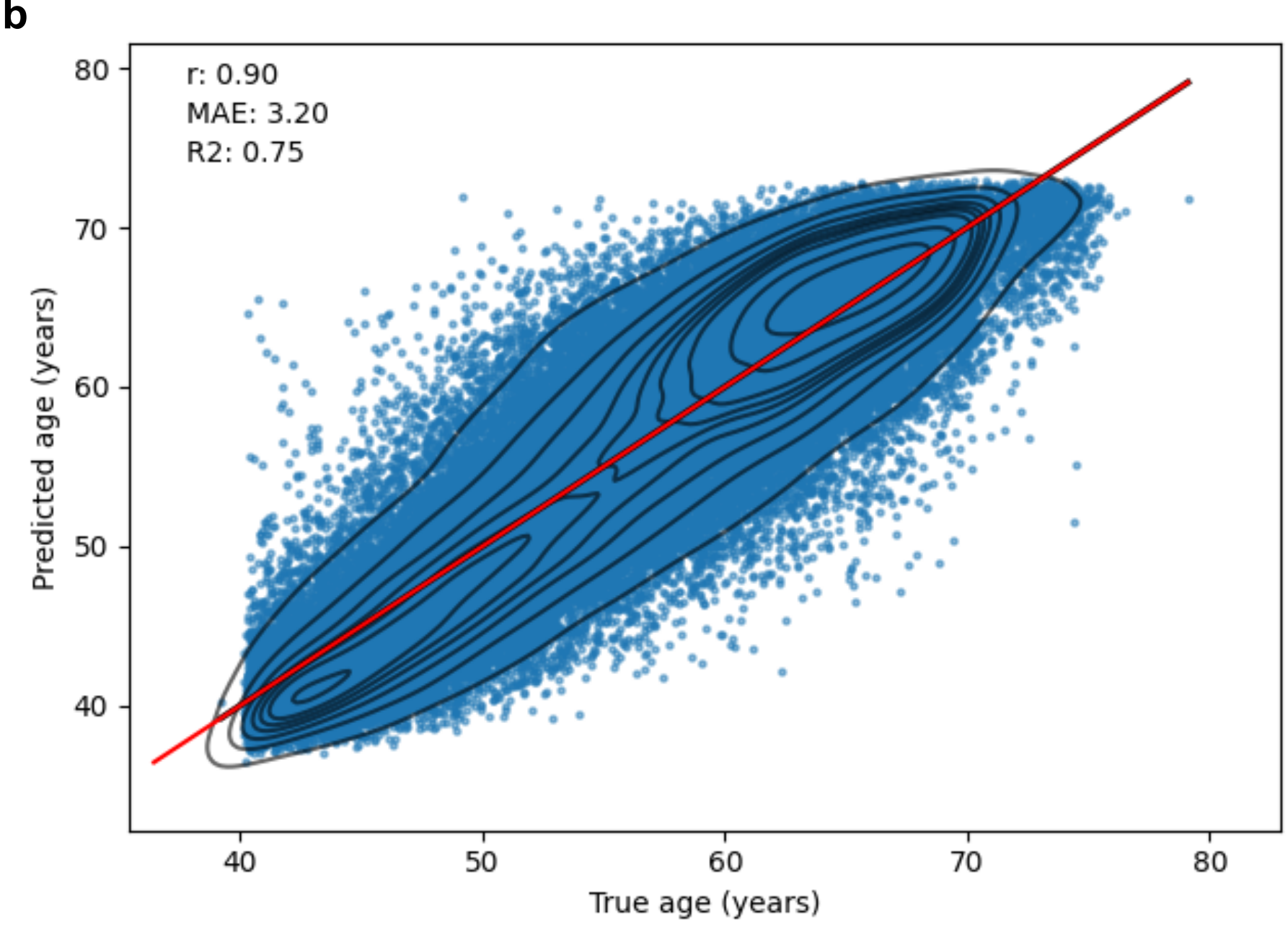

Supplementary Figure 1. Predicted age against chronological (true) age (n=71,343). Scatterplots with density lines are shown (**a**) before and (**b**) after correction for the regression-to-the-mean bias, in the combined sexes model in the UK Biobank. The red line is the diagonal, while the black line is the linear regression fit line (both lines are superimposed in **b**). r: Pearson's correlation, MAE: mean absolute error, R2: coefficient of determination.

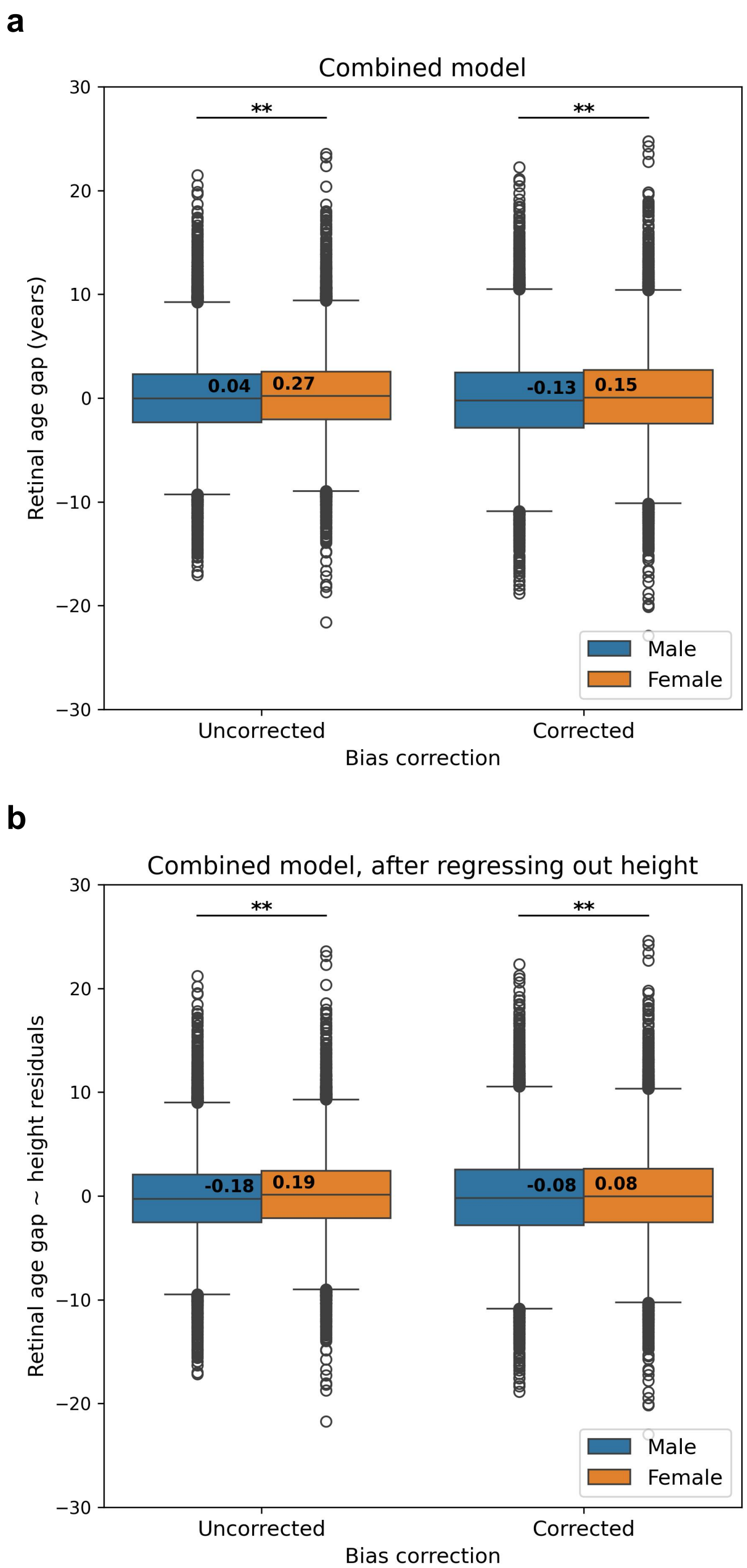

Supplementary Figure 2. Comparison of retinal age gap between age-matched females (n=32,918) and males (n=32,918). Retinal age gap was estimated from the combined-sample model, with and without correction for the regression-to-the-mean bias, (**a**) before and (**b**) after regressing out height from the retinal age gap. Boxes represent the interquartile range (IQR; 25th–75th percentiles), with the central line indicating the median. Whiskers extend to the most extreme data points within 1.5 x IQR, and points beyond the whiskers are shown as outliers. The mean is indicated by the annotated value. **: $p<0.001$ from two-sided t-test comparing sexes. Source data are provided as a Source Data file.

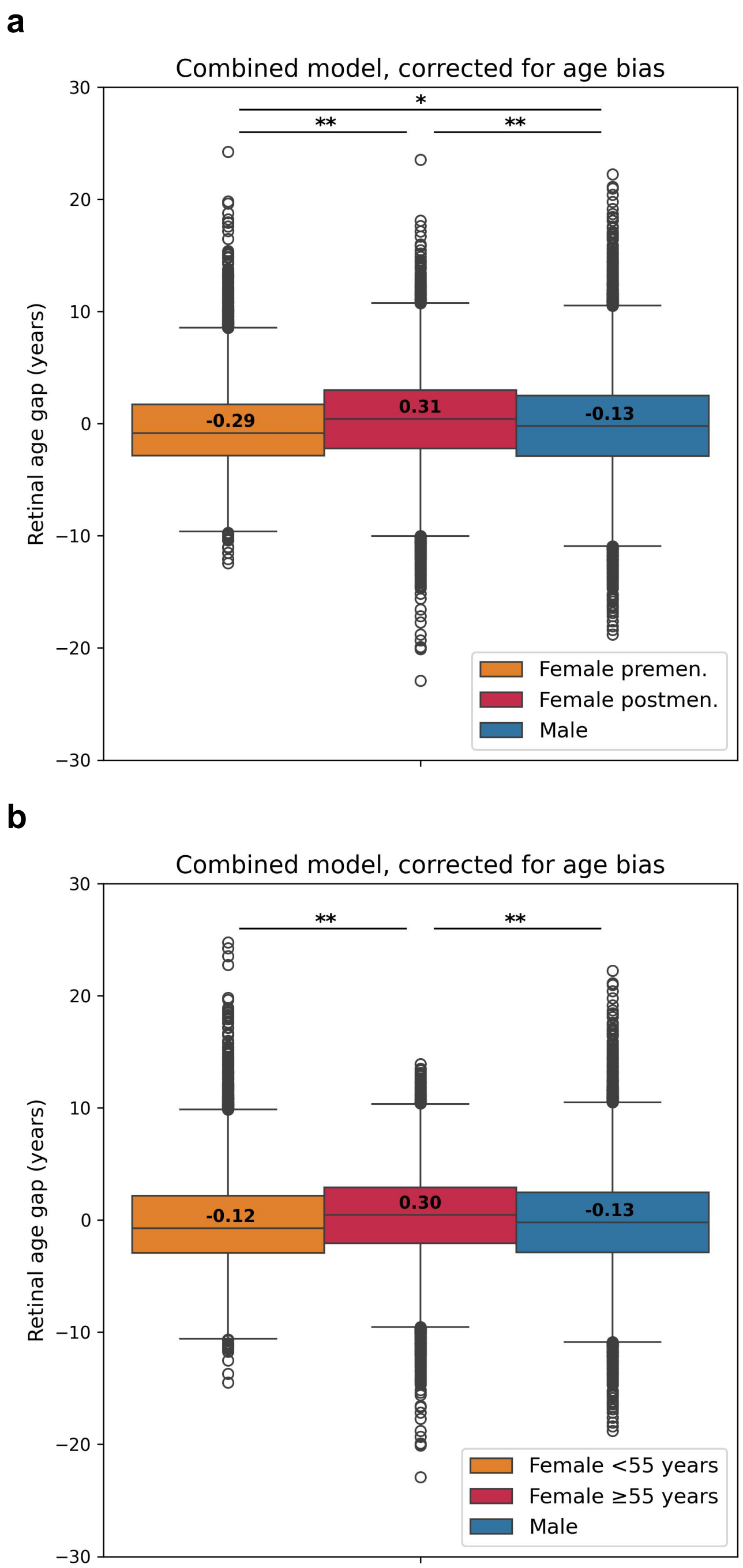

Supplementary Figure 3. Comparison of retinal age gap between female strata and males. Females were stratified by (**a**) menopause status—premenopausal (n=6757) vs postmenopausal (n=19,929)—and (**b**) age—<55 (n=11,662) vs ≥55 years (n=21,256). Retinal age gap was estimated from the combined-sample model, after correction for the regression-to-the-mean bias. Boxes represent the interquartile range (IQR; 25th–75th percentiles), with the central line indicating the median. Whiskers extend to the most extreme data points within 1.5 x IQR, and points beyond the whiskers are shown as outliers. The mean is indicated by the annotated value. *: p<0.05, **: p<0.001 from two-sided t-test comparing groups. premen.: premenopausal, postmen.: postmenopausal. Source data are provided as a Source Data file.

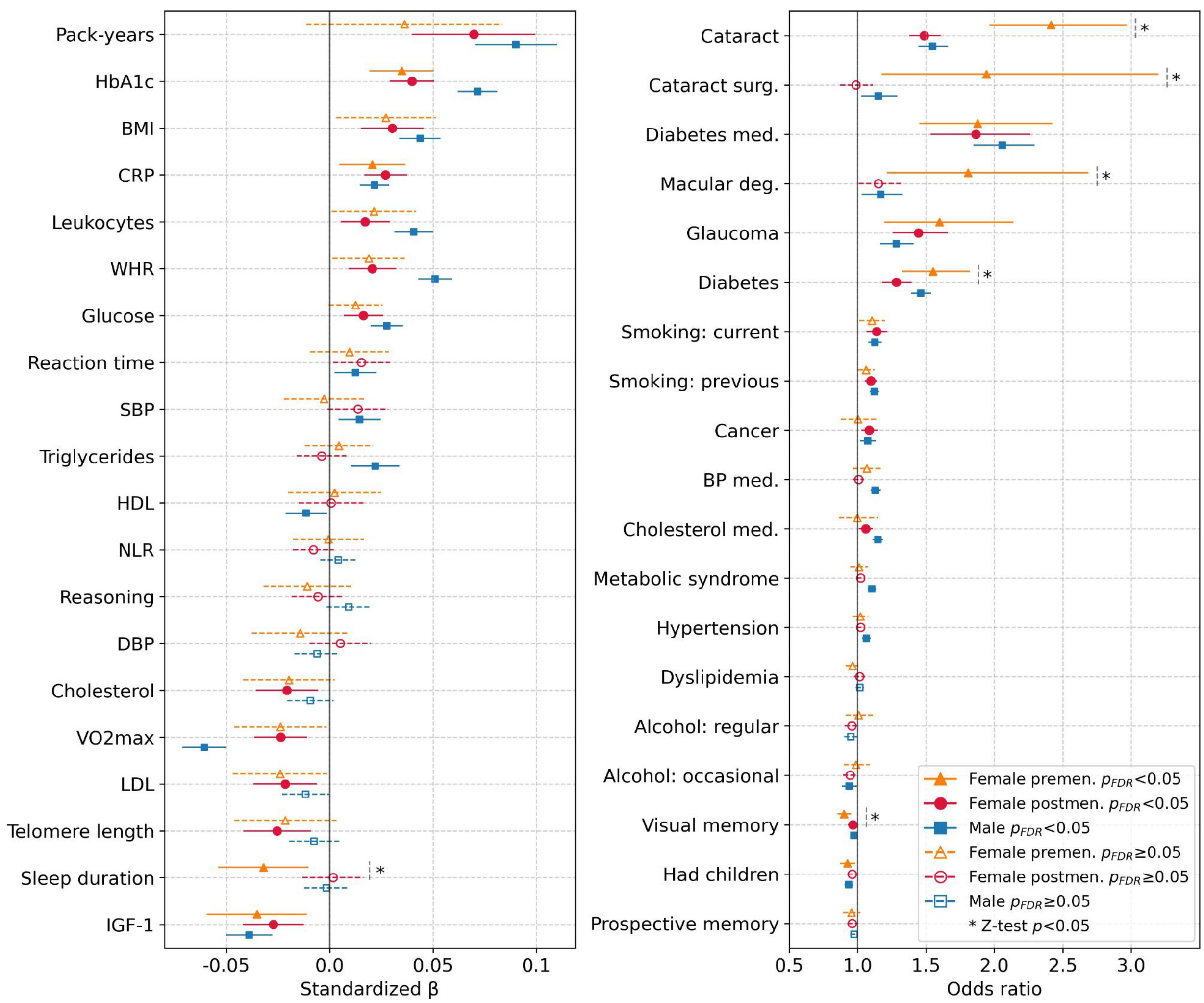

Supplementary Figure 4. Models linking retinal age gap to risk factors and diseases in females stratified by menopause status and males (for visual comparison). Ordinary least square linear regression (left panel) and logistic regression (right panel) models were adjusted for socio-demographic factors. Outcomes are ordered by average effect size between the two female strata. Error bars represent the 95% confidence interval. Effects from premenopausal and postmenopausal females were compared using a two-sided *z*-test. Sample sizes and detailed results are shown in Supplementary Tables 2–3. BMI: body mass index, BP: blood pressure, CRP: C-reactive protein, DBP: diastolic blood pressure, deg.: degeneration, FDR: false discovery rate, HDL: high-density lipoprotein, IGF-1: insulin-like growth factor 1, LDL: low-density lipoprotein, med.: medication, NLR: neutrophil-to-lymphocyte ratio, premen.: premenopausal, postmen.: postmenopausal, SBP: systolic blood pressure, surg.: surgery, WHR: waist-to-hip ratio. Source data are provided as a Source Data file.

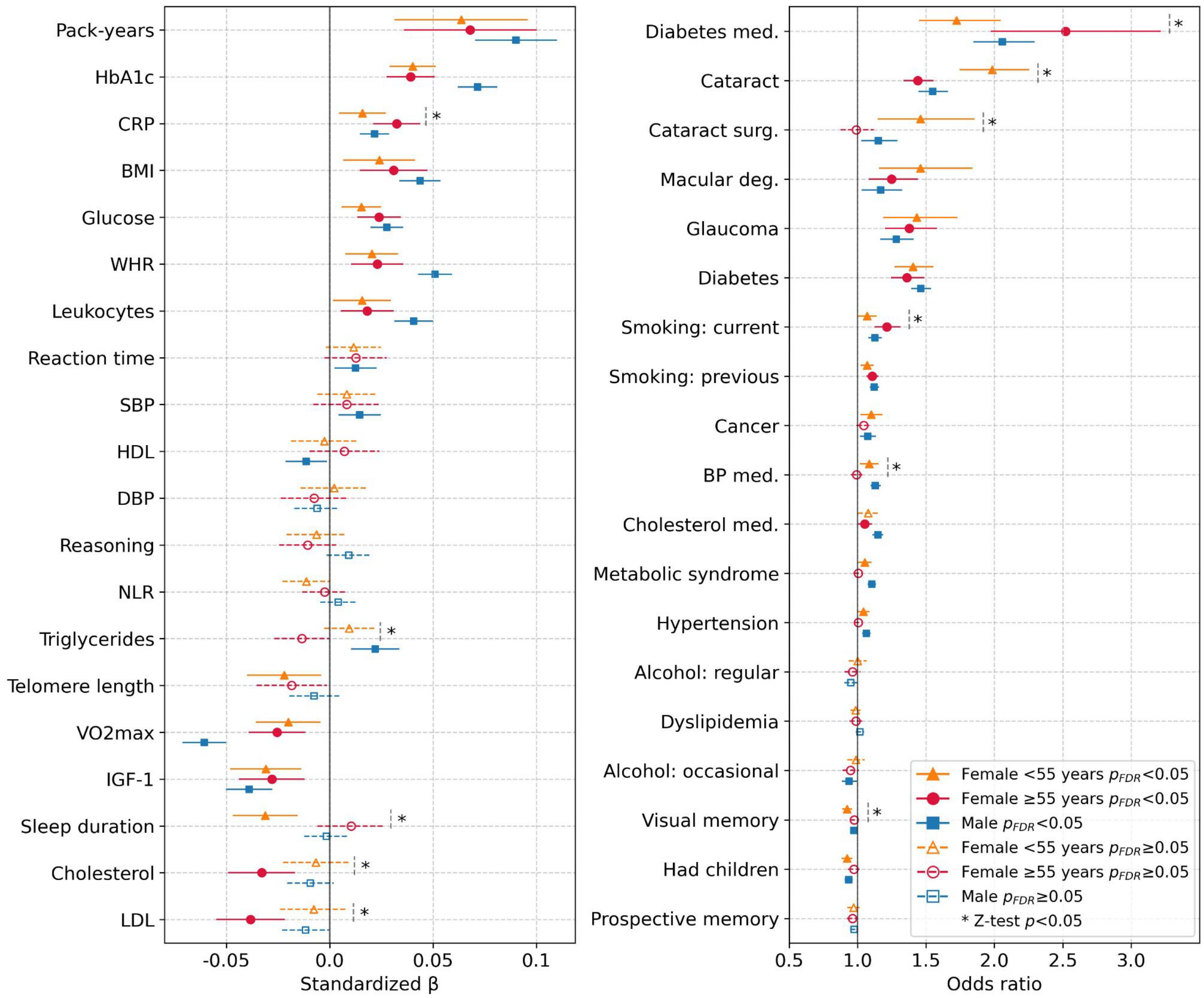

Supplementary Figure 5. Models linking retinal age gap to risk factors and diseases in females stratified by age (<55 vs ≥55 years) and males (for visual comparison). Ordinary least square linear regression (left panel) and logistic regression (right panel) models were adjusted for socio-demographic factors. Outcomes are ordered by average effect size between the two female strata. Error bars represent the 95% confidence interval. Effects from younger and older females were compared using a two-sided z-test. Sample sizes and detailed results are shown in Supplementary Tables 2–3. BMI: body mass index, BP: blood pressure, CRP: C-reactive protein, DBP: diastolic blood pressure, deg.: degeneration, FDR: false discovery rate, HDL: high-density lipoprotein, IGF-1: insulin-like growth factor 1, LDL: low-density lipoprotein, med.: medication, NLR: neutrophil-to-lymphocyte ratio, SBP: systolic blood pressure, surg.: surgery, WHR: waist-to-hip ratio. Source data are provided as a Source Data file.

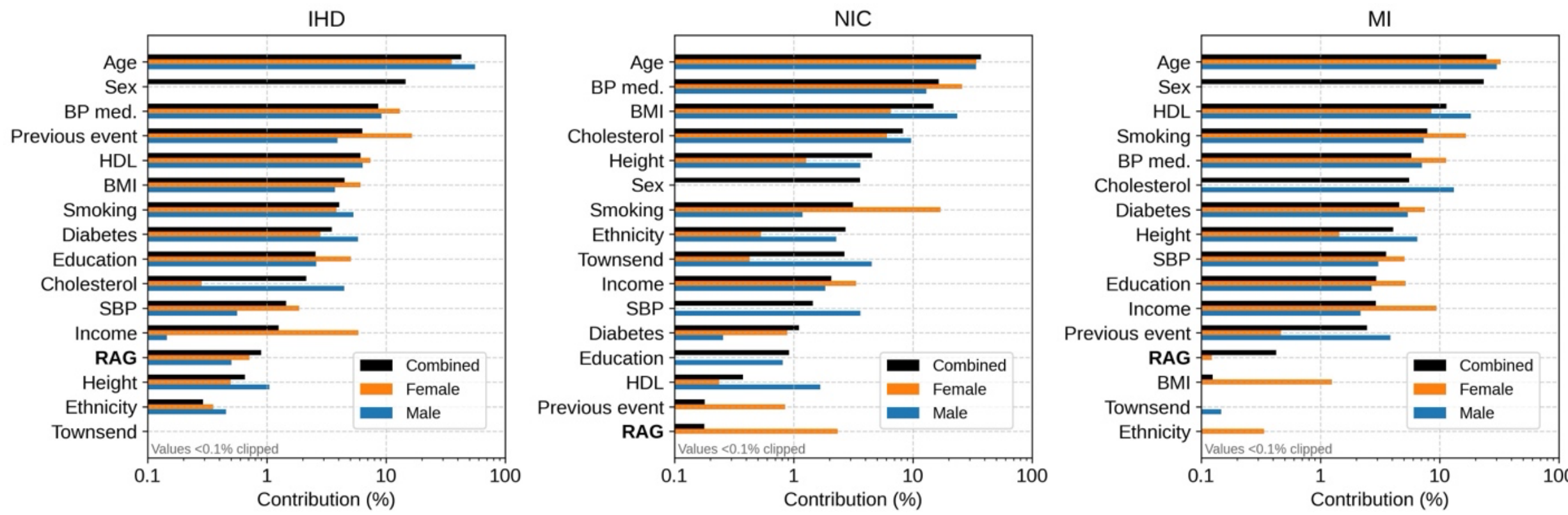

Supplementary Figure 6. Relative contribution of predictors in Cox proportional hazards models for additional cardiovascular outcomes. Relative contribution of predictors to incident ischemic heart disease, myocardial infarction, and non-ischemic cardiomyopathy was quantified as the percent change in model χ^2 when each variable is removed from the multivariable Cox model. The analysis uses the same leave-one-out procedure as in Figure 3b–c. Detailed results are presented in Supplementary Table 5. BMI: body mass index, BP med.: blood pressure medication, HDL: high-density lipoprotein, IHD: ischemic heart disease, MI: myocardial infarction, NIC: non-ischemic cardiomyopathy, RAG: retinal age gap, SBP: systolic blood pressure. Source data are provided as a Source Data file.

a

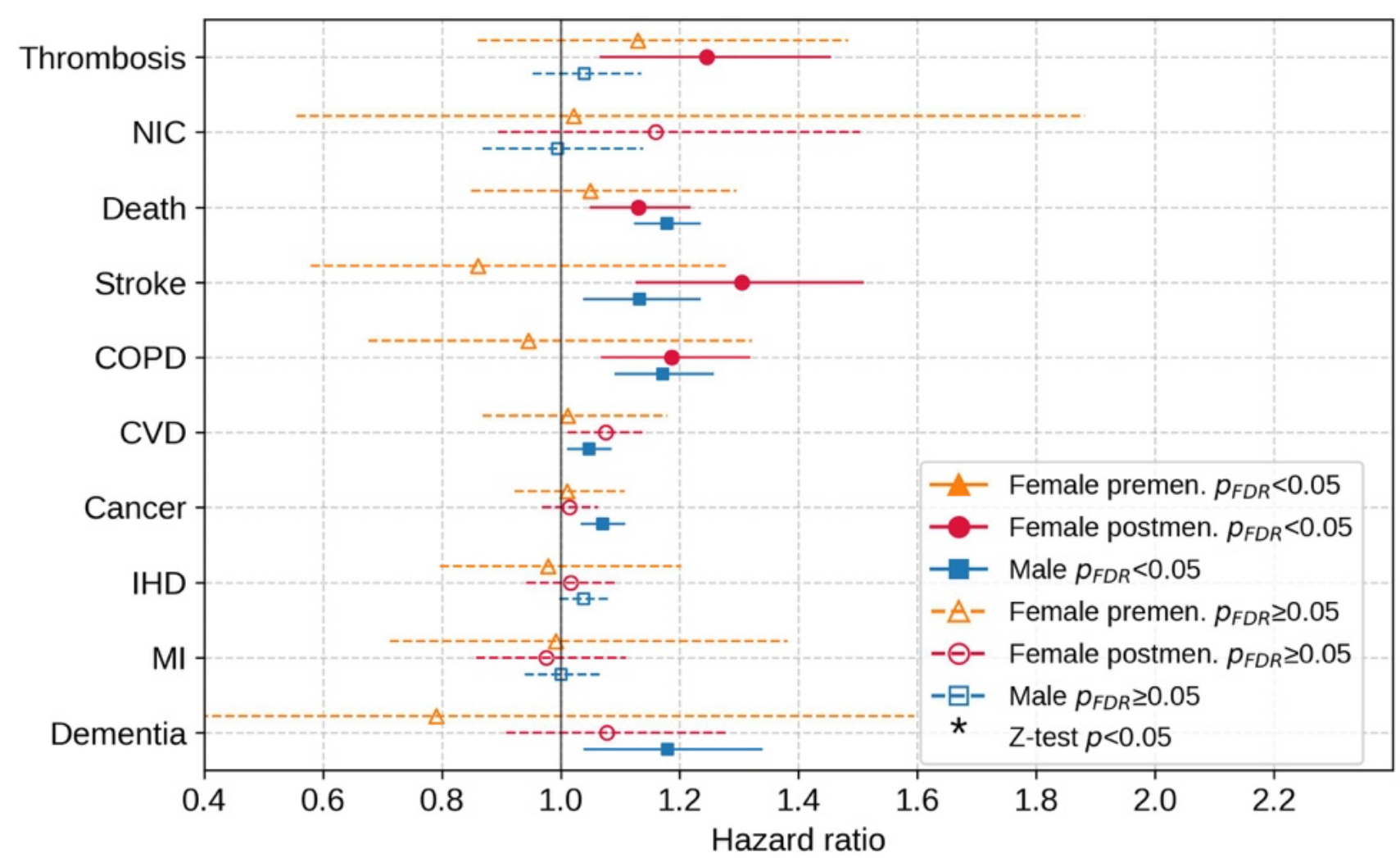

b

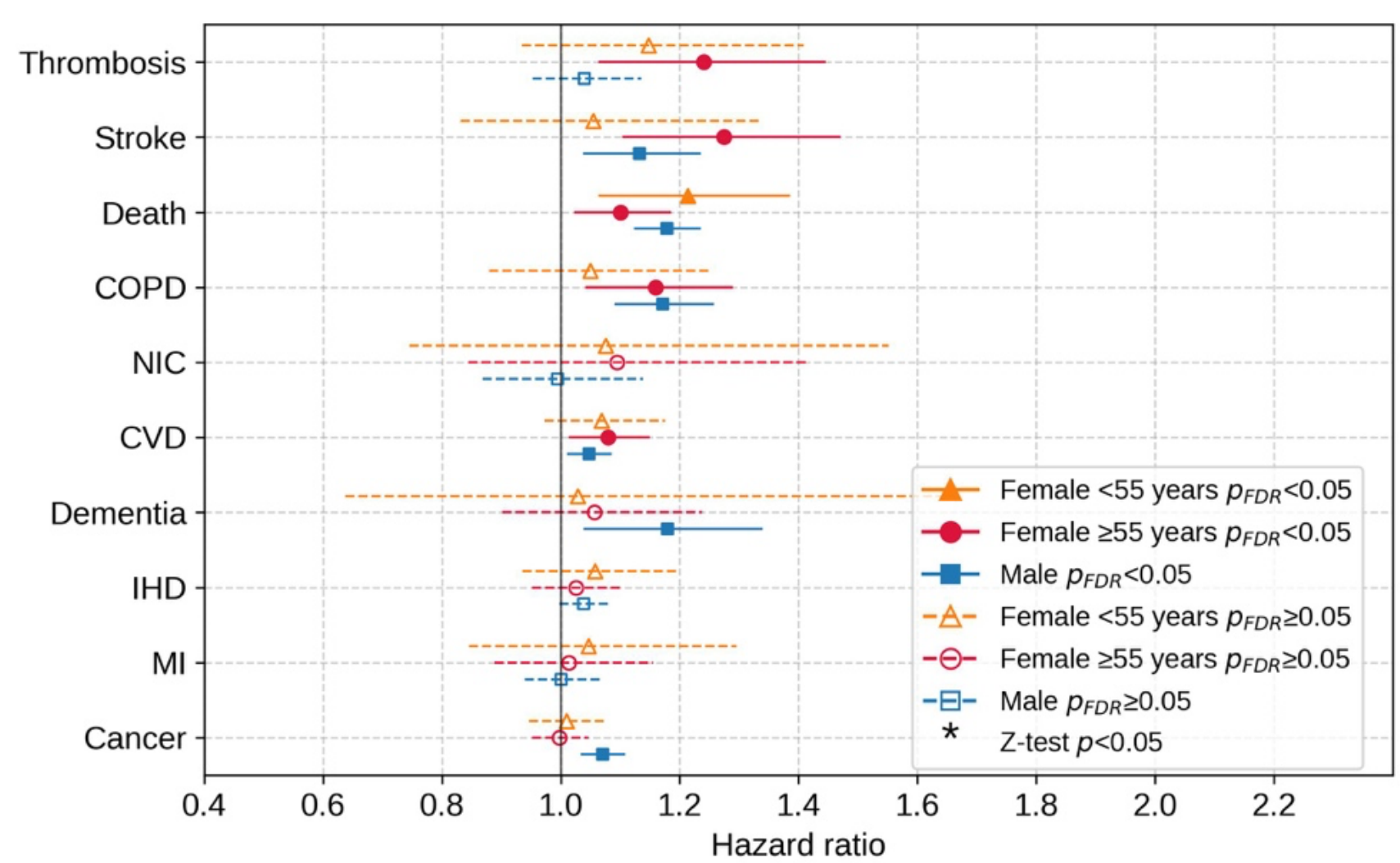

Supplementary Figure 7. Retinal age gap associations with mortality and disease outcomes in female strata. Hazard ratios (HR) from Cox proportional hazards regression analyses linking retinal age gap to mortality and diseases are shown for females stratified by (**a**) menopause status and (**b**) age (<55 vs ≥55 years), and males (for visual comparison). Models were adjusted for socio-demographic factors, common cardiovascular risk factors, and previous events from the same category. Outcomes are ordered by average HR between the two female strata. Error bars represent the 95% confidence interval. Effects from the female strata were compared using a two-sided *z*-test and were not significant. Sample sizes, number of events, and detailed results are shown in Supplementary Table 4. COPD: chronic obstructive pulmonary disease, CVD: cardiovascular disease, FDR: false discovery rate, IHD: ischemic heart disease, MI: myocardial infarction, NIC: non-ischemic cardiomyopathy. Source data are provided as a Source Data file.

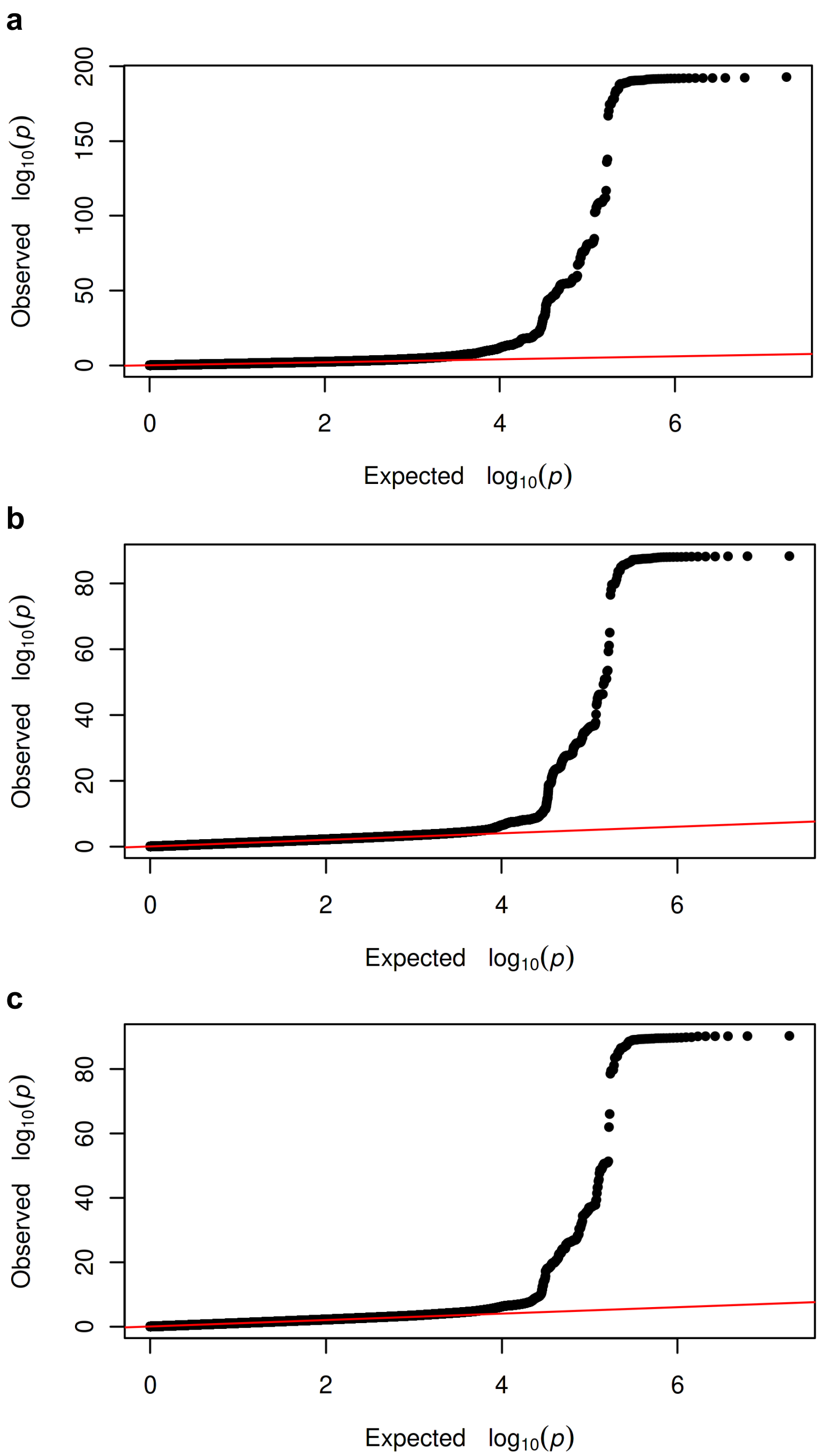

Supplementary Figure 8. QQ plot of retinal age gap GWAS. a Combined sample. **b** Female sample. **c** Male sample.

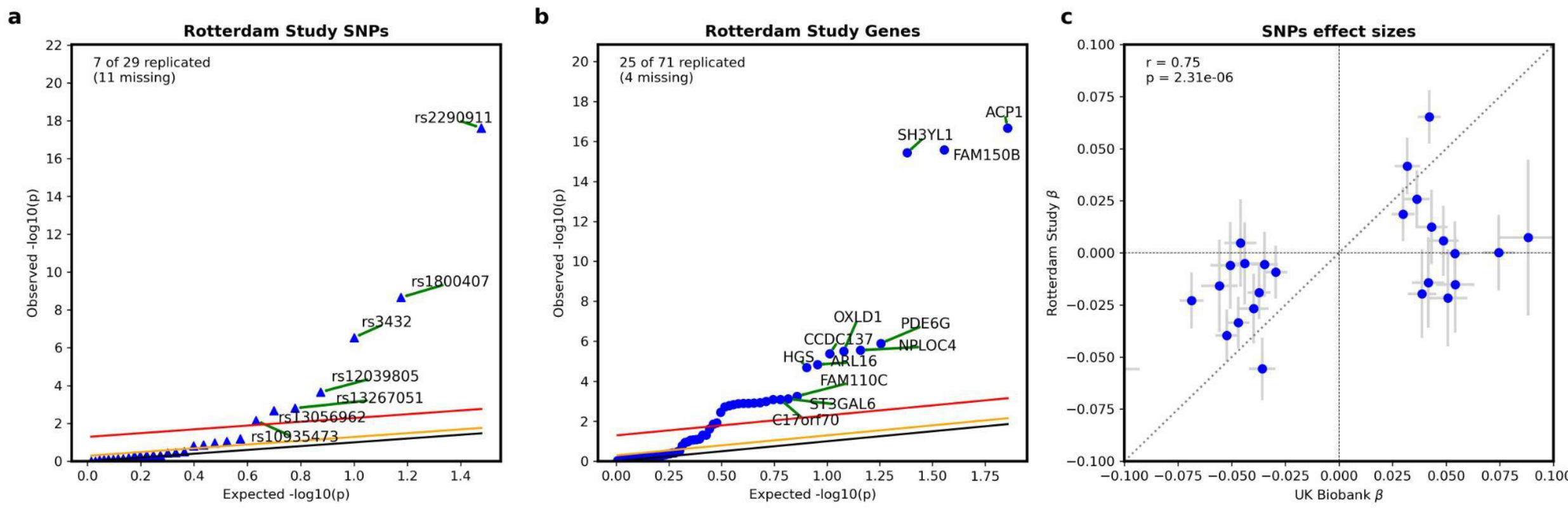

Supplementary Figure 9. Replication of genetic analysis in the Rotterdam Study (n=9547). Replication of (**a**) variants (SNPs) and (**b**) genes that were significant in the UK Biobank. The red line represents a false discovery rate (FDR) of 0.05, the orange line represents an FDR of 0.5, and the black line represents observed = expected. The label "missing" indicates that those SNPs or genes were significant in the UK Biobank, but not available in the Rotterdam Study. **c** Effect sizes (betas) of significant SNPs in the UK Biobank against their effect sizes in the Rotterdam Study. Error bars represent standard errors. Pearson's correlation between the two cohorts was 0.75 (*p*<0.0001). Detailed results are presented in Supplementary Tables 12–13. Source data are provided as a Source Data file.

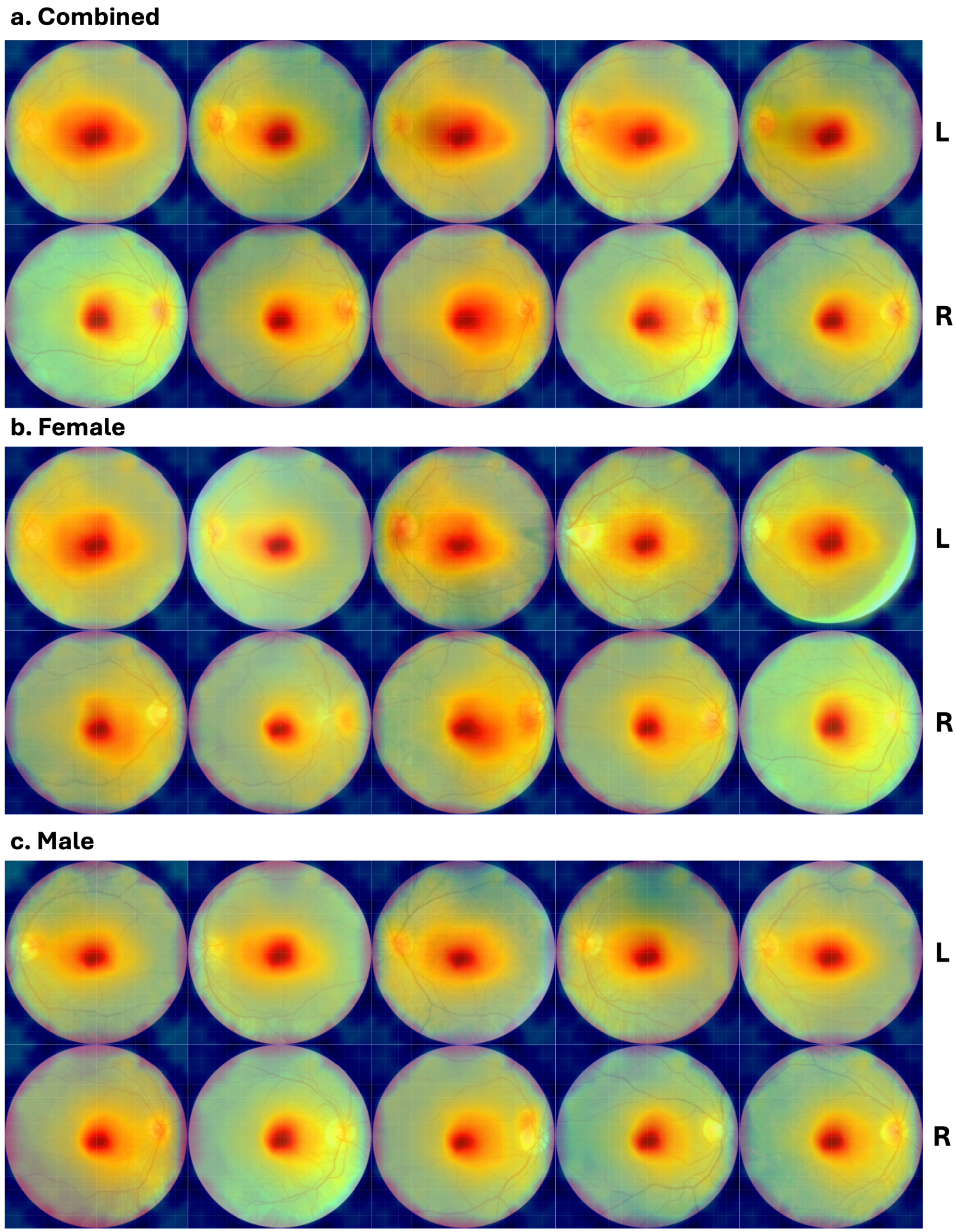

Supplementary Figure 10. Group-average saliency maps in the 5 folds of the fine-tuned models. Smoothed pixel-level saliency maps, obtained by bicubic interpolation from patch-level maps, are projected on a reference fundus image for (**a**) the combined, (**b**) female, and (**c**) male models. Each column represents a model fold. Left eye averages are shown on upper rows (L), right eye averages on lower rows (R).

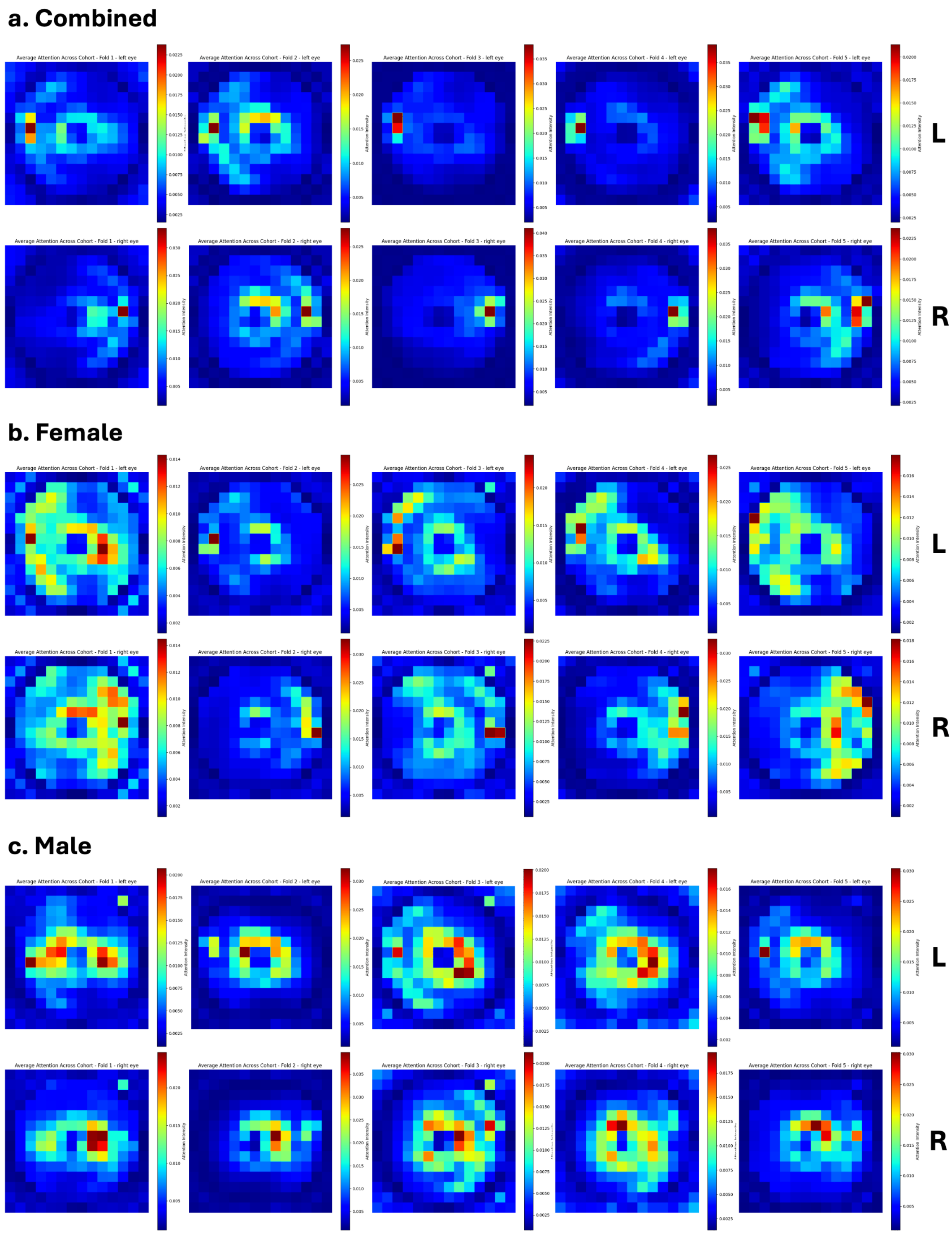

Supplementary Figure 11. Group-average patch-level attention maps in the 5 folds of the fine-tuned models. Attention maps, averaged across heads and subjects are shown for (**a**) the combined, (**b**) female, and (**c**) male models for left (L, upper rows) and right (R, lower rows). Each column represents the last attention block of a model fold.

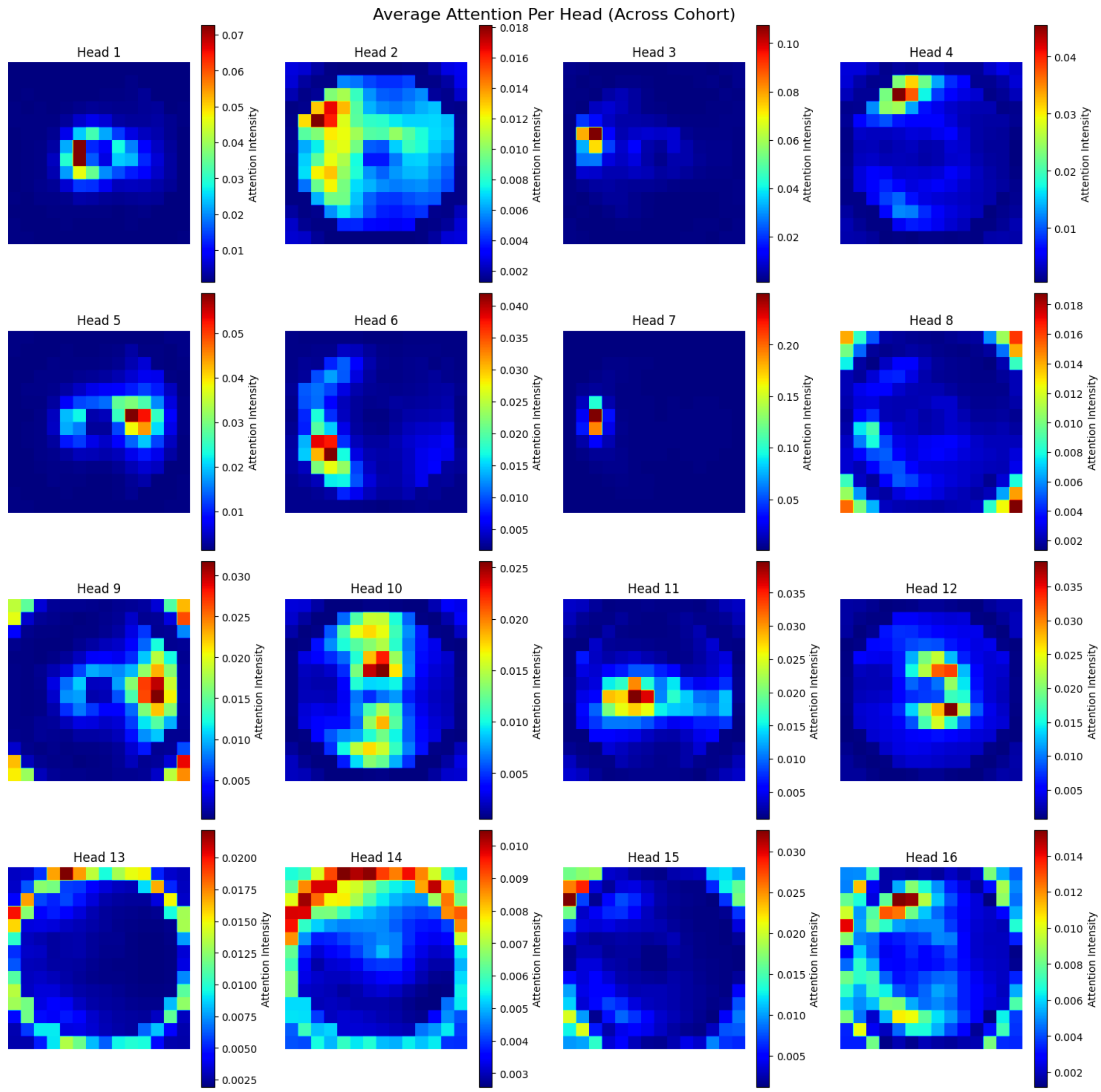

Supplementary Figure 12. Group-average left eye attention in separate heads of the combined model's fold 1, last attention block.

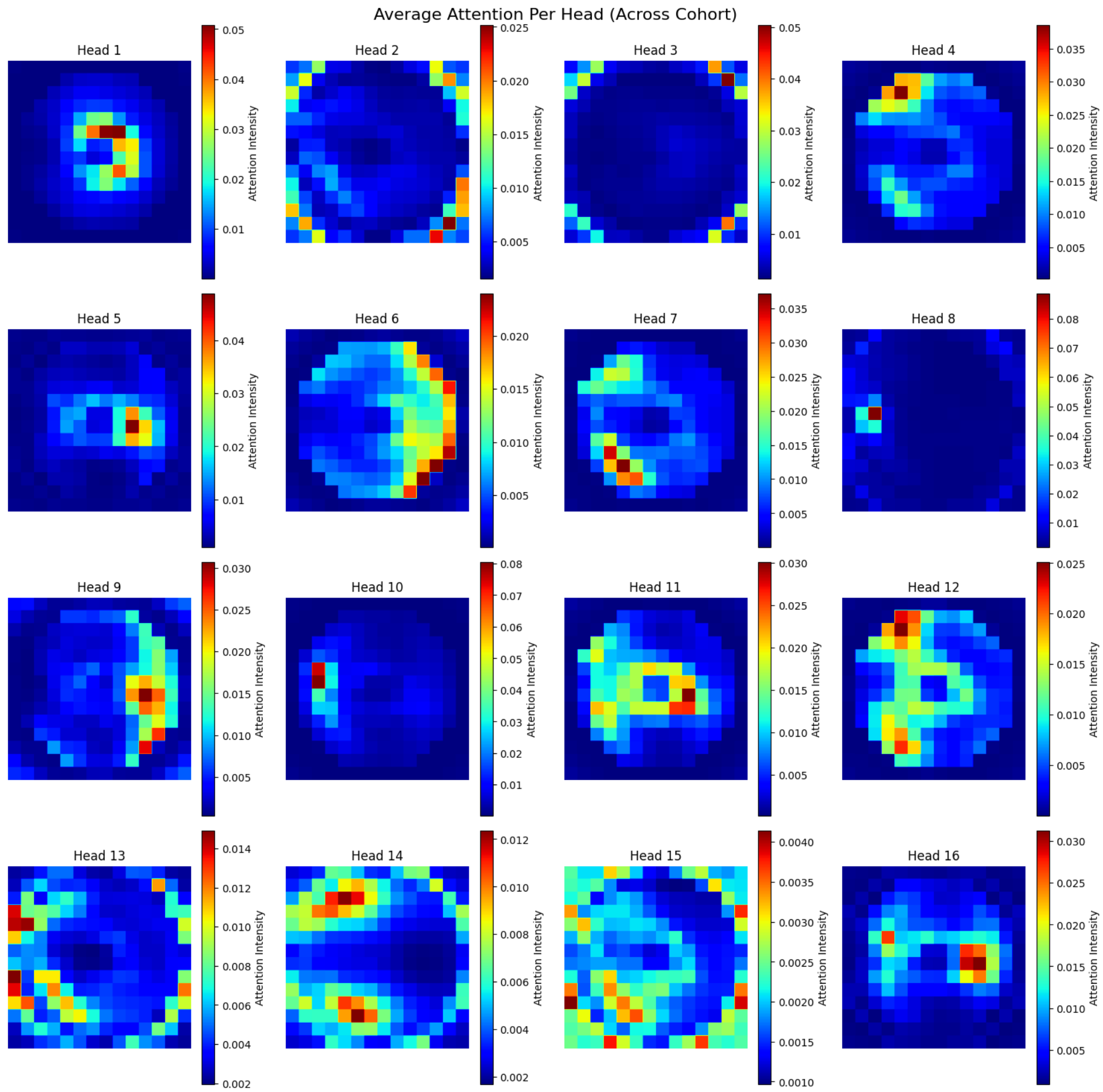

Supplementary Figure 13. Group-average left eye attention in separate heads of the female model's fold 1, last attention block.

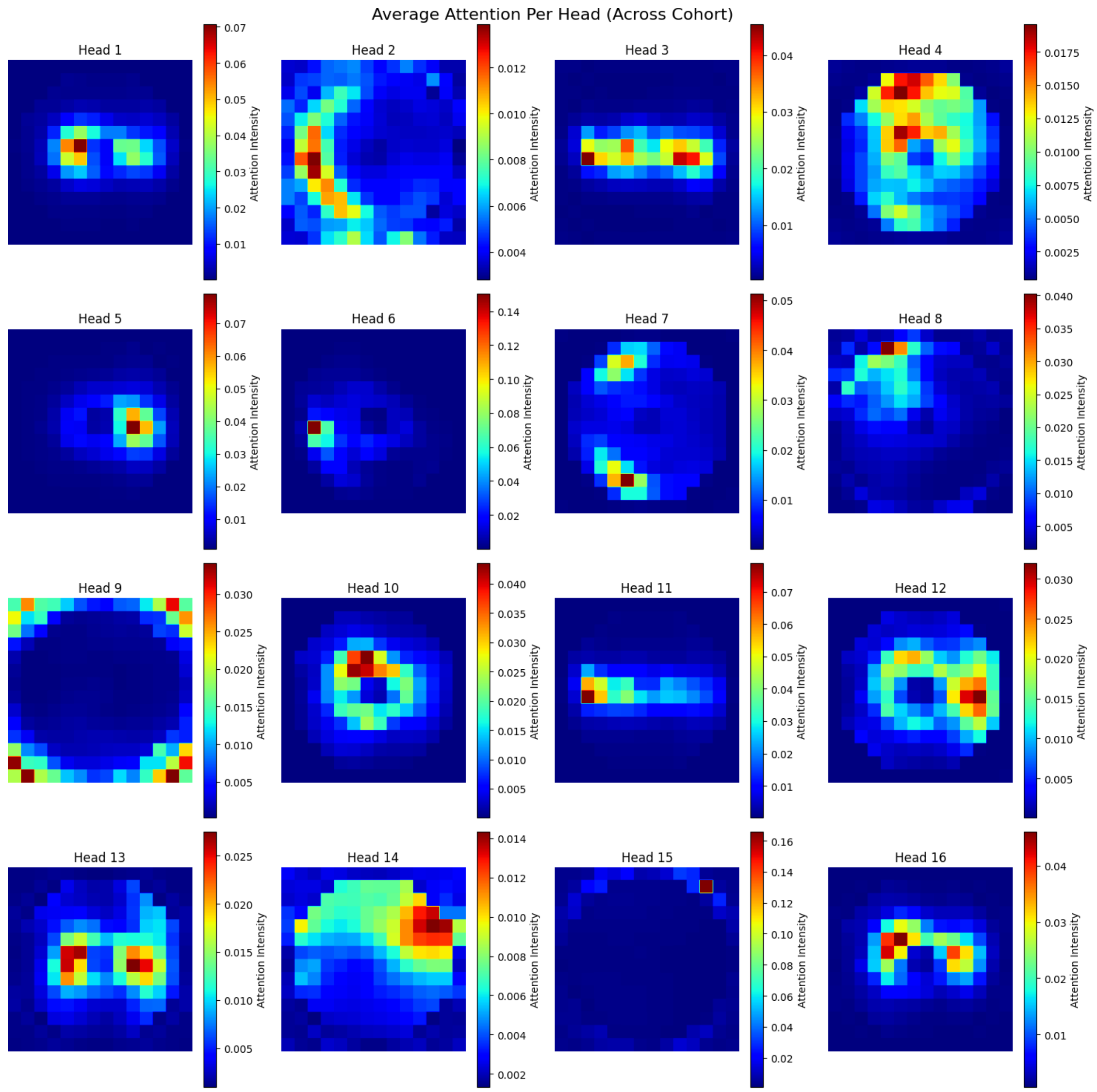

Supplementary Figure 14. Group-average left eye attention in separate heads of the male model's fold 1, last attention block.

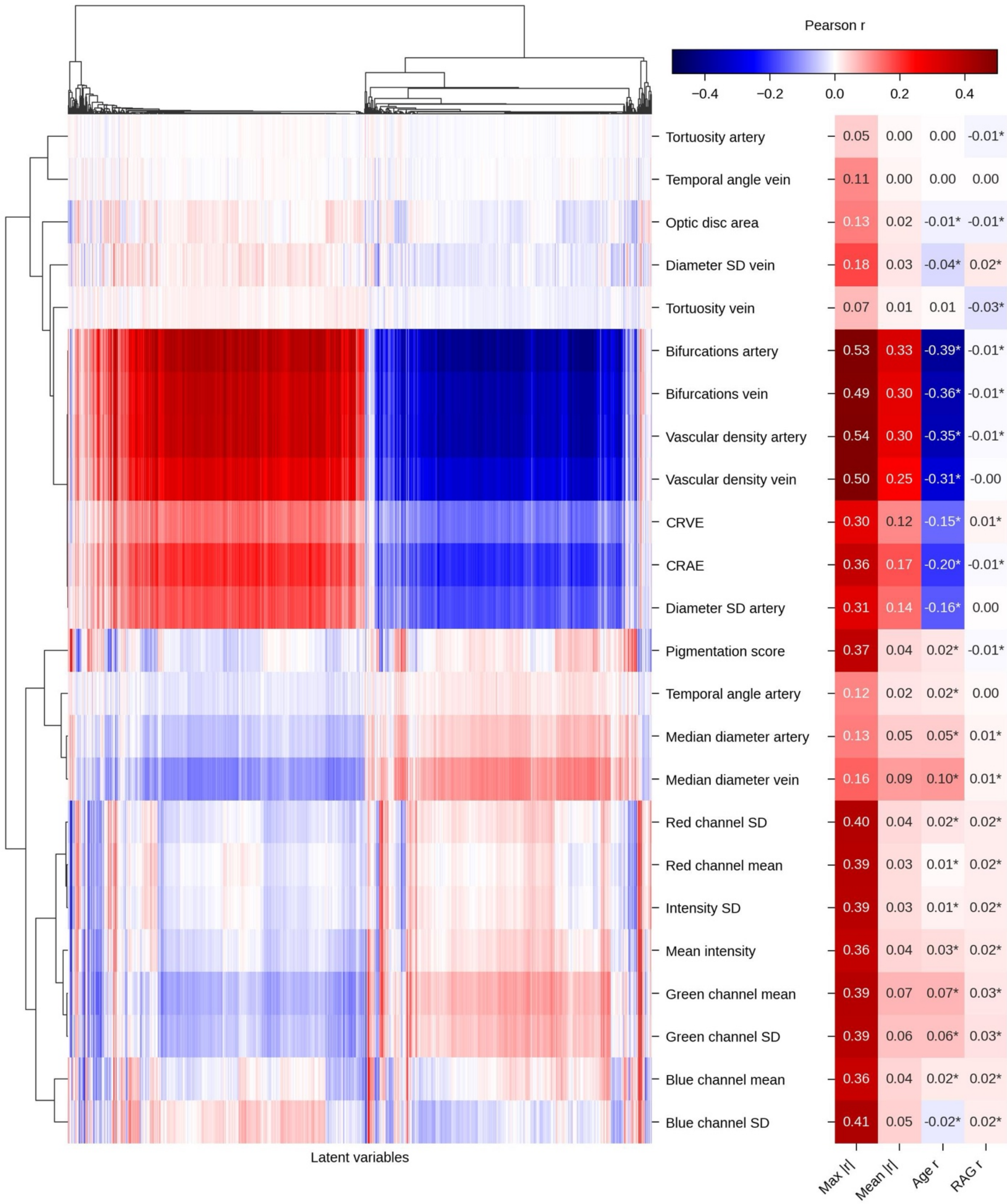

Supplementary Figure 15. **Fold 2 correlation map between the combined model's 1024 latent variables and interpretable image features (n=24,084 images).** For every image feature, the maximum and mean absolute correlation with latent variables, the correlation with chronological age and retinal age gap are shown on the right side of the figure. CRAE: central retinal arterial equivalent, CRVE: central retinal venous equivalent, RAG: retinal age gap, SD: standard deviation, *: $p<0.05$.

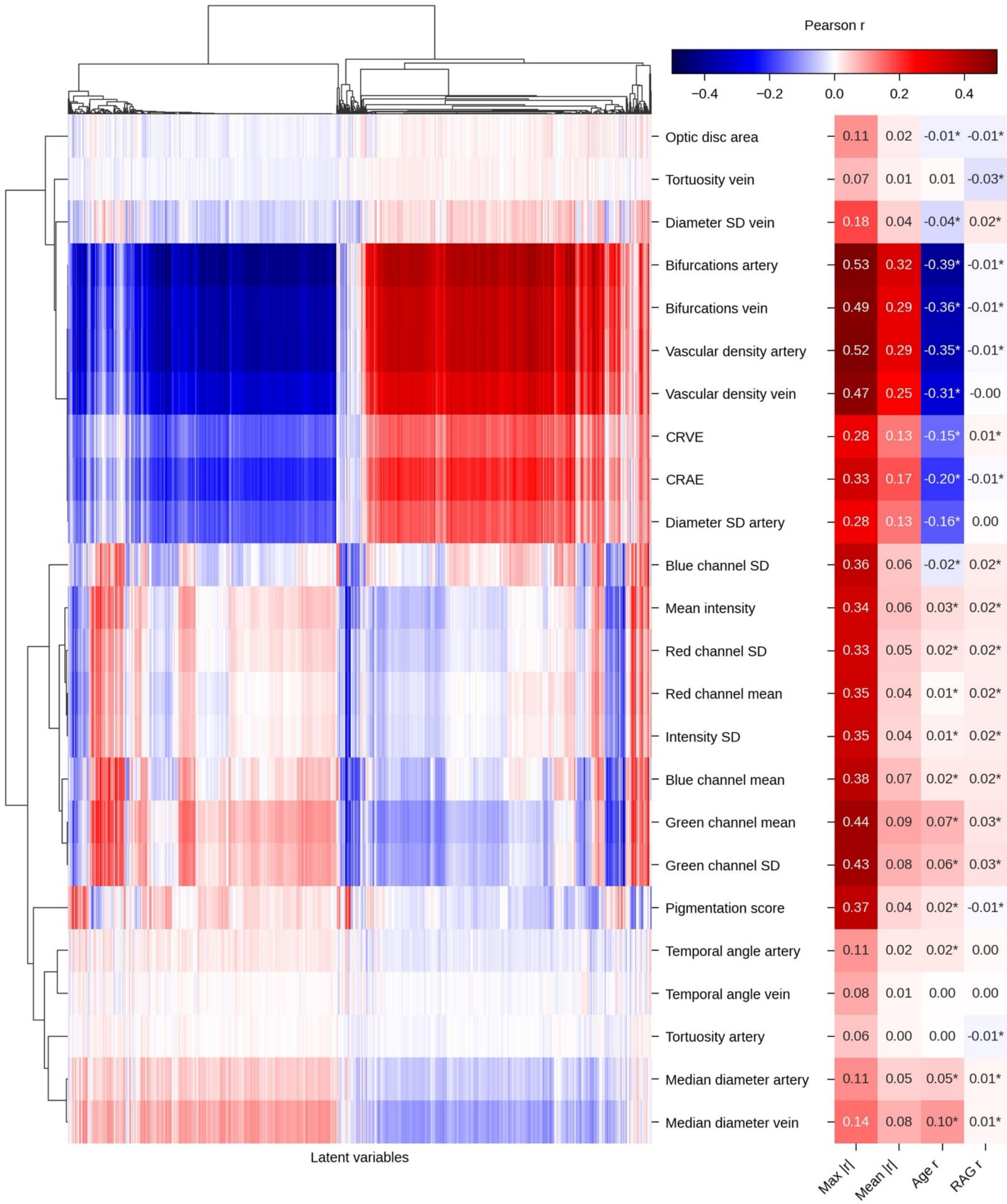

Supplementary Figure 16. **Fold 3 correlation map between the combined model's 1024 latent variables and interpretable image features (n=24,022 images).** For every image feature, the maximum and mean absolute correlation with latent variables, the correlation with chronological age and retinal age gap are shown on the right side of the figure. CRAE: central retinal arterial equivalent, CRVE: central retinal venous equivalent, RAG: retinal age gap, SD: standard deviation, *: p<0.05.

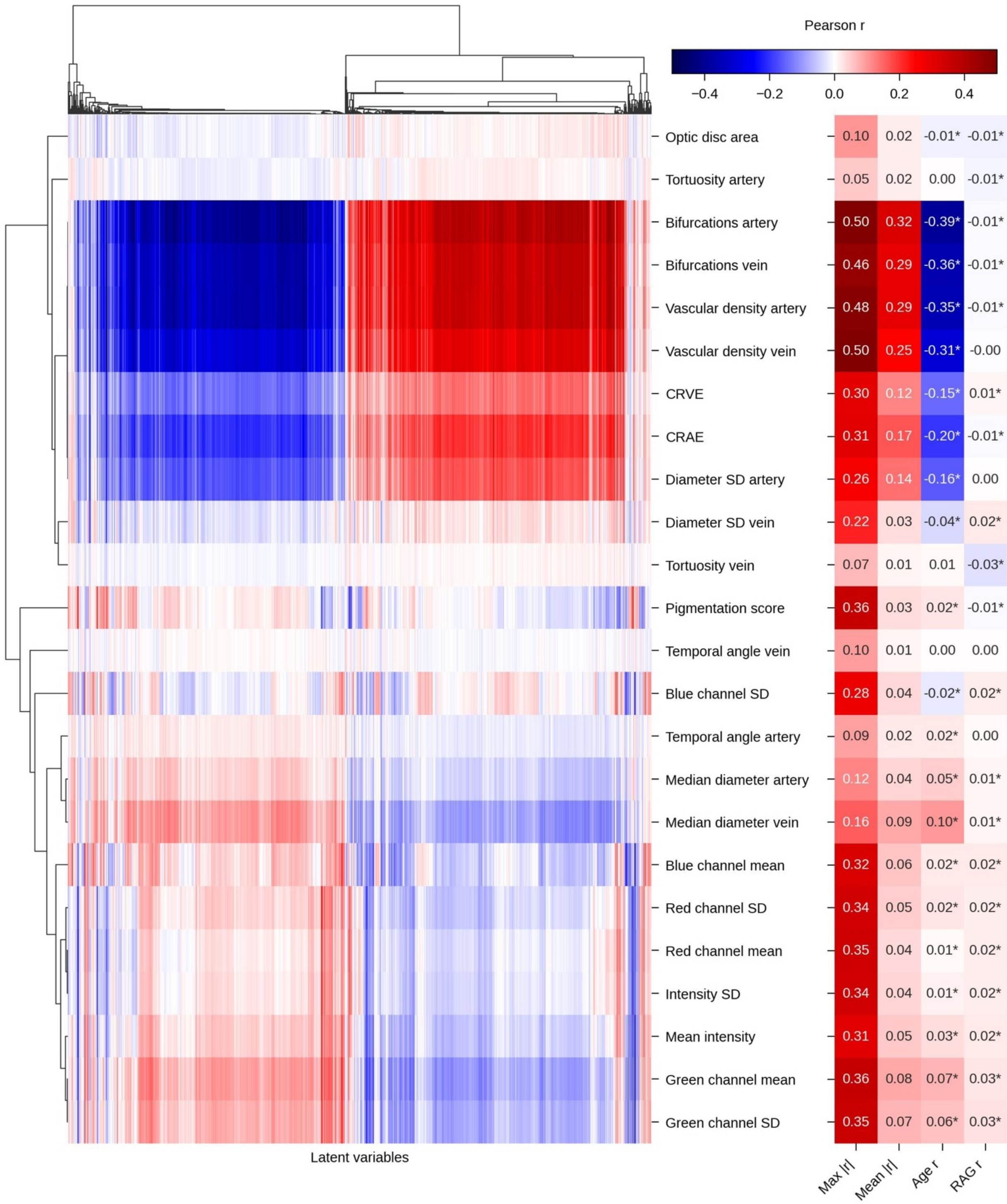

Supplementary Figure 17. **Fold 4 correlation map between the combined model's 1024 latent variables and interpretable image features (n=23,990 images).** For every image feature, the maximum and mean absolute correlation with latent variables, the correlation with chronological age and retinal age gap are shown on the right side of the figure. CRAE: central retinal arterial equivalent, CRVE: central retinal venous equivalent, RAG: retinal age gap, SD: standard deviation, *: p<0.05.

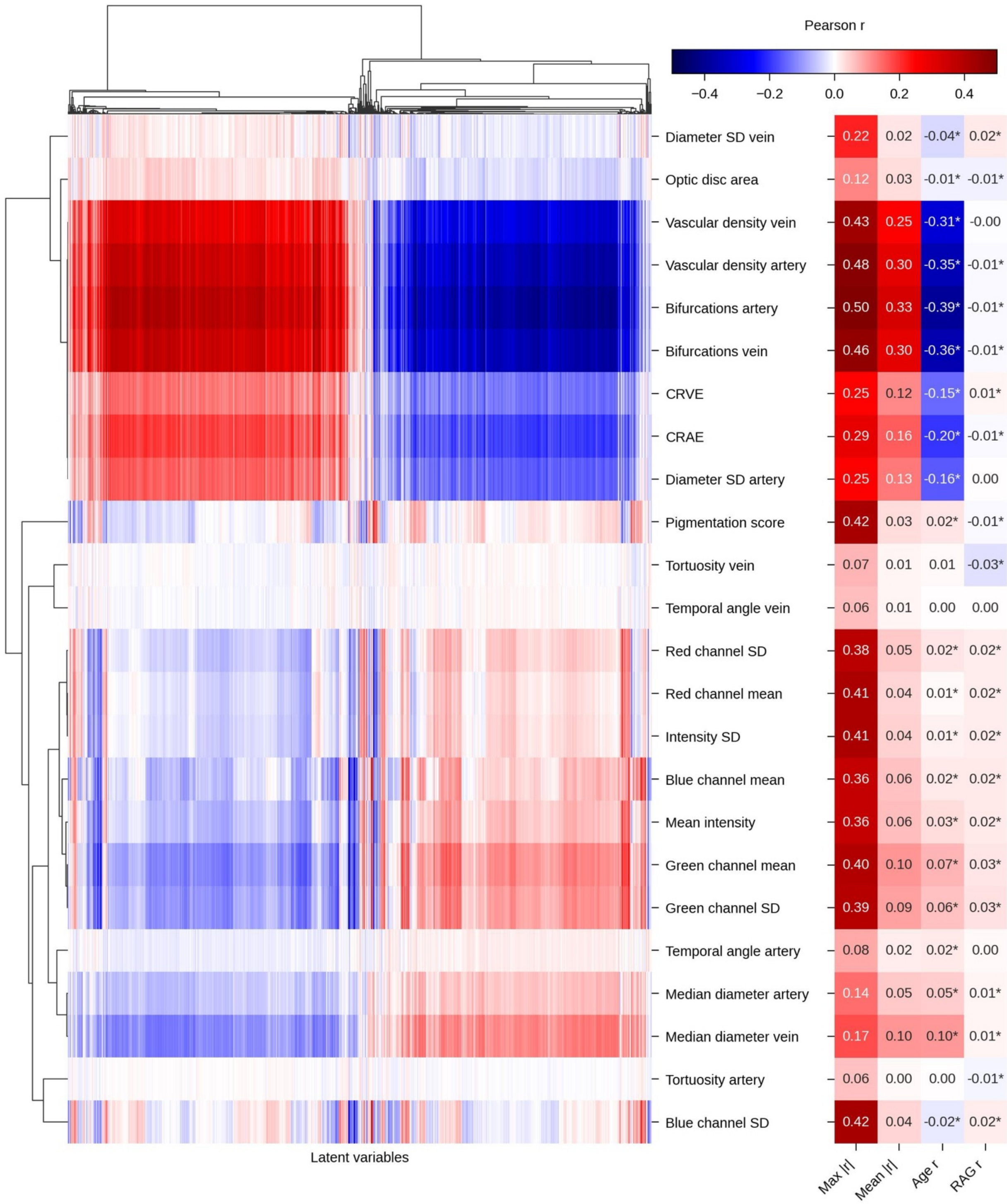

Supplementary Figure 18. **Fold 5 correlation map between the combined model's 1024 latent variables and interpretable image features (n=24,140 images).** For every image feature, the maximum and mean absolute correlation with latent variables, the correlation with chronological age and retinal age gap are shown on the right side of the figure. CRAE: central retinal arterial equivalent, CRVE: central retinal venous equivalent, RAG: retinal age gap, SD: standard deviation, *: p<0.05.

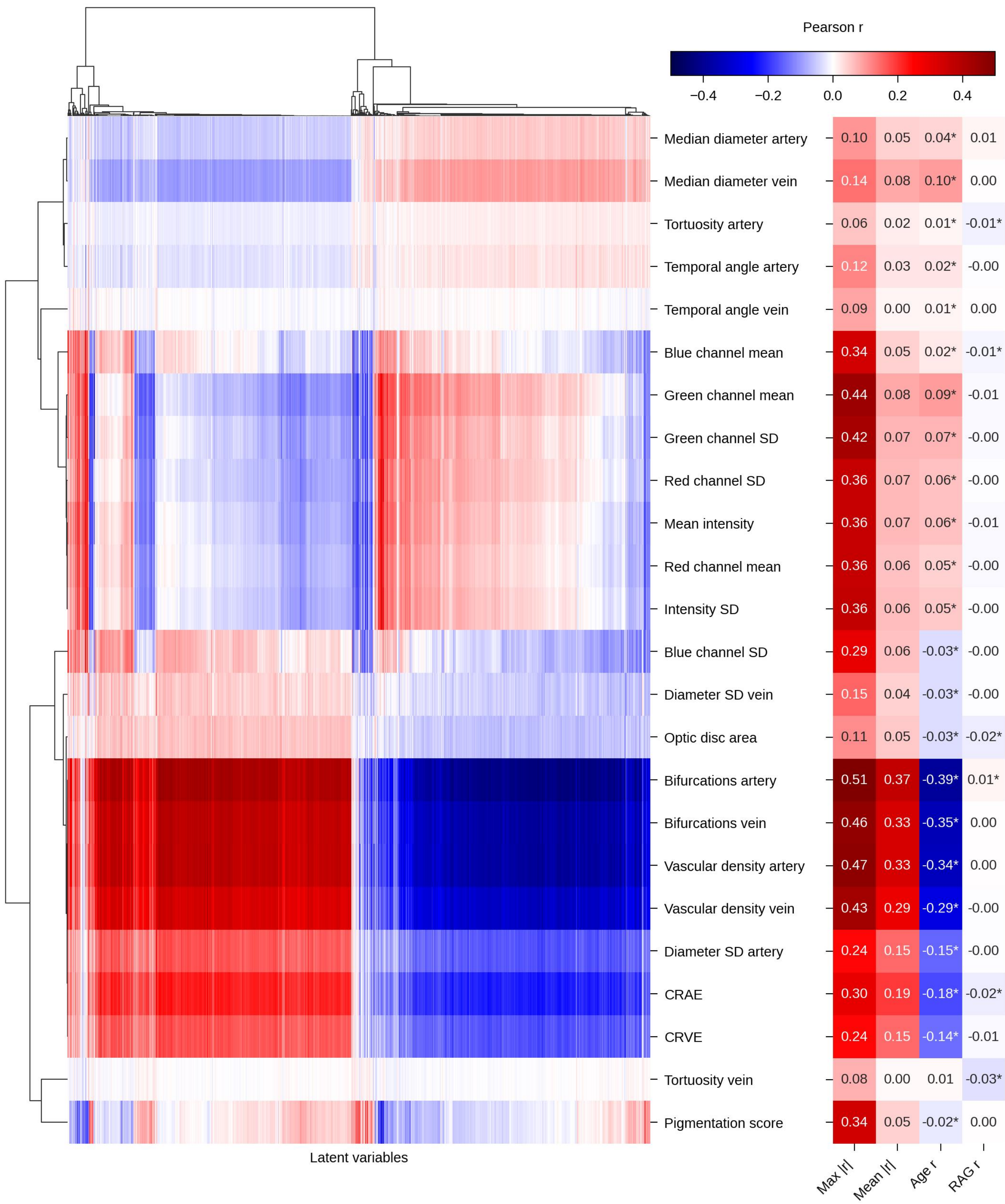

Supplementary Figure 19. Fold 1 correlation map between the female model's 1024 latent variables and interpretable image features (n=10,642 images). For every image feature, the maximum and mean absolute correlation with latent variables, the correlation with chronological age and retinal age gap are shown on the right side of the figure. CRAE: central retinal arterial equivalent, CRVE: central retinal venous equivalent, RAG: retinal age gap, SD: standard deviation, *: $p<0.05$.

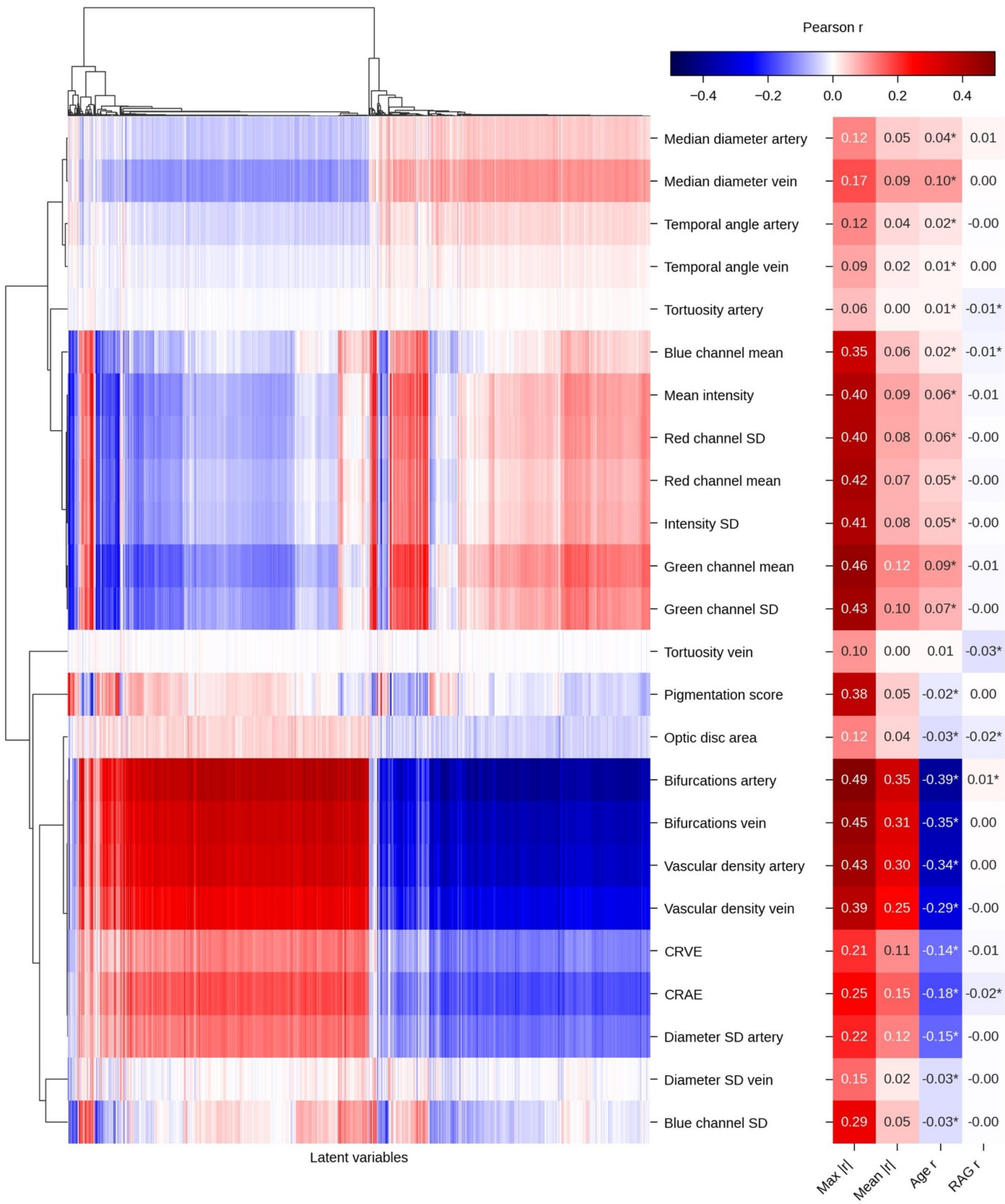

Supplementary Figure 20. **Fold 2 correlation map between the female model's 1024 latent variables and interpretable image features (n=10,786 images).** For every image feature, the maximum and mean absolute correlation with latent variables, the correlation with chronological age and retinal age gap are shown on the right side of the figure. CRAE: central retinal arterial equivalent, CRVE: central retinal venous equivalent, RAG: retinal age gap, SD: standard deviation, *: $p<0.05$.

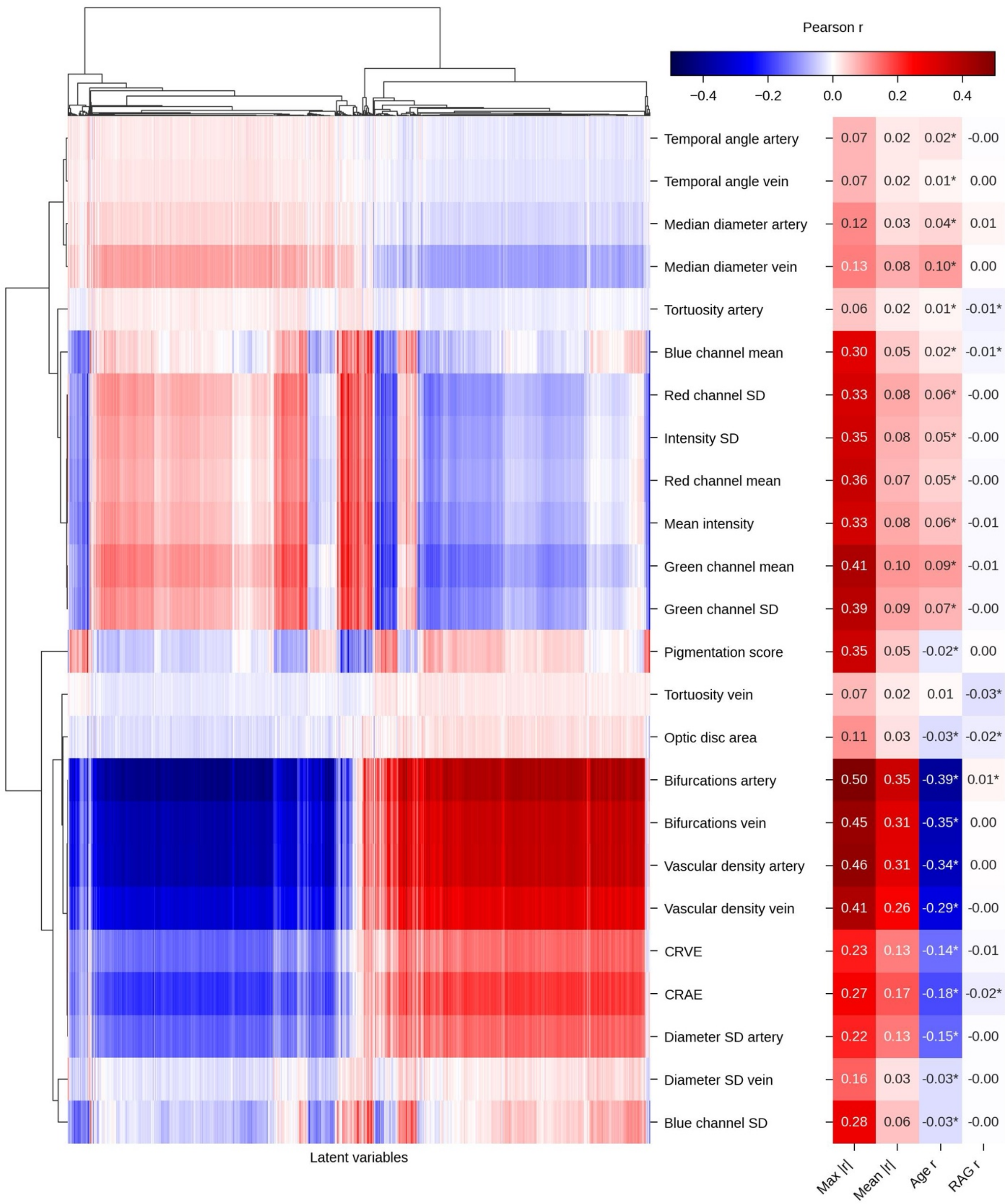

Supplementary Figure 21. **Fold 3 correlation map between the female model's 1024 latent variables and interpretable image features (n=10,781 images).** For every image feature, the maximum and mean absolute correlation with latent variables, the correlation with chronological age and retinal age gap are shown on the right side of the figure. CRAE: central retinal arterial equivalent, CRVE: central retinal venous equivalent, RAG: retinal age gap, SD: standard deviation, *: p<0.05.

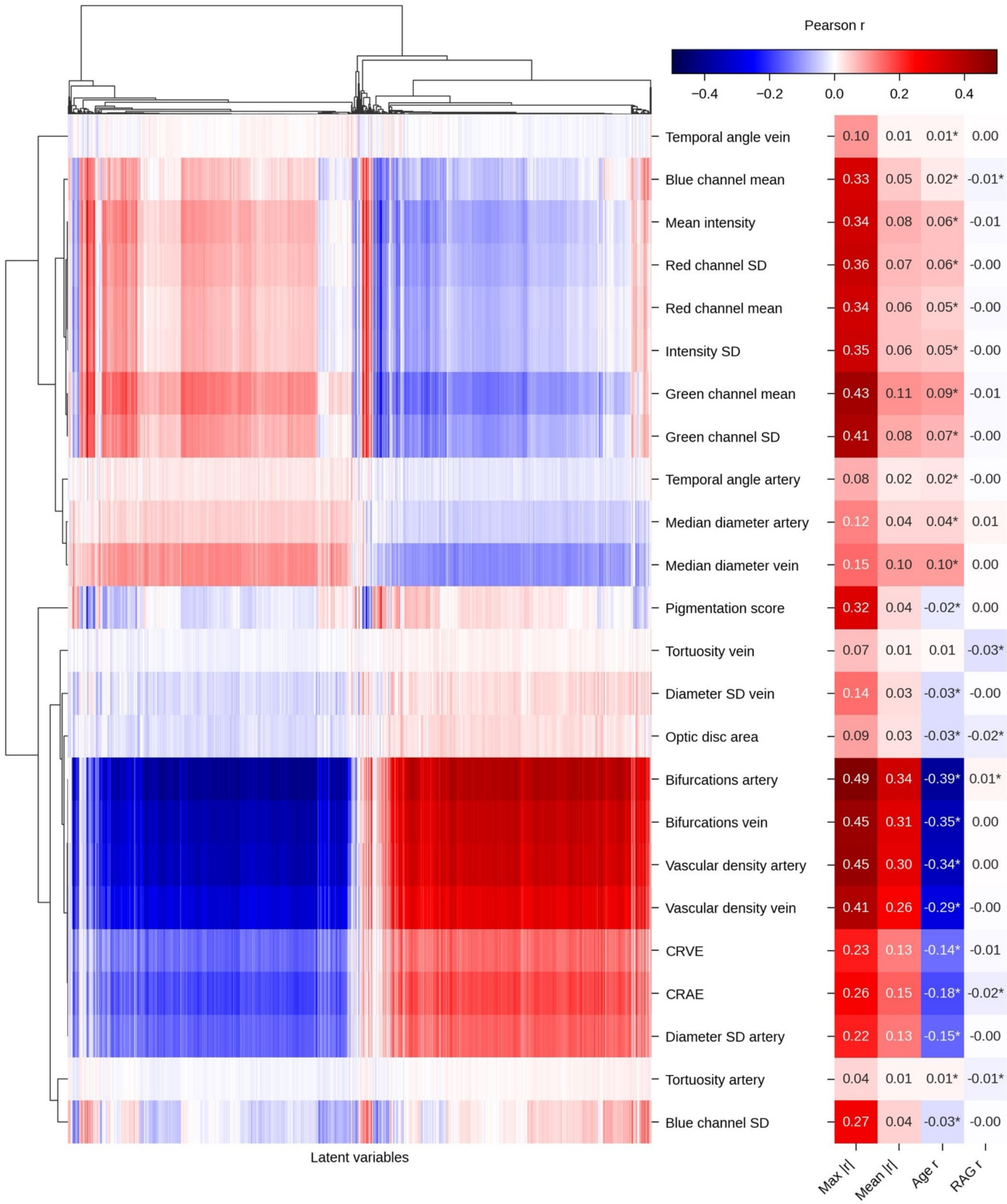

Supplementary Figure 22. **Fold 4 correlation map between the female model's 1024 latent variables and interpretable image features (n=10,587 images).** For every image feature, the maximum and mean absolute correlation with latent variables, the correlation with chronological age and retinal age gap are shown on the right side of the figure. CRAE: central retinal arterial equivalent, CRVE: central retinal venous equivalent, RAG: retinal age gap, SD: standard deviation, *: p<0.05.

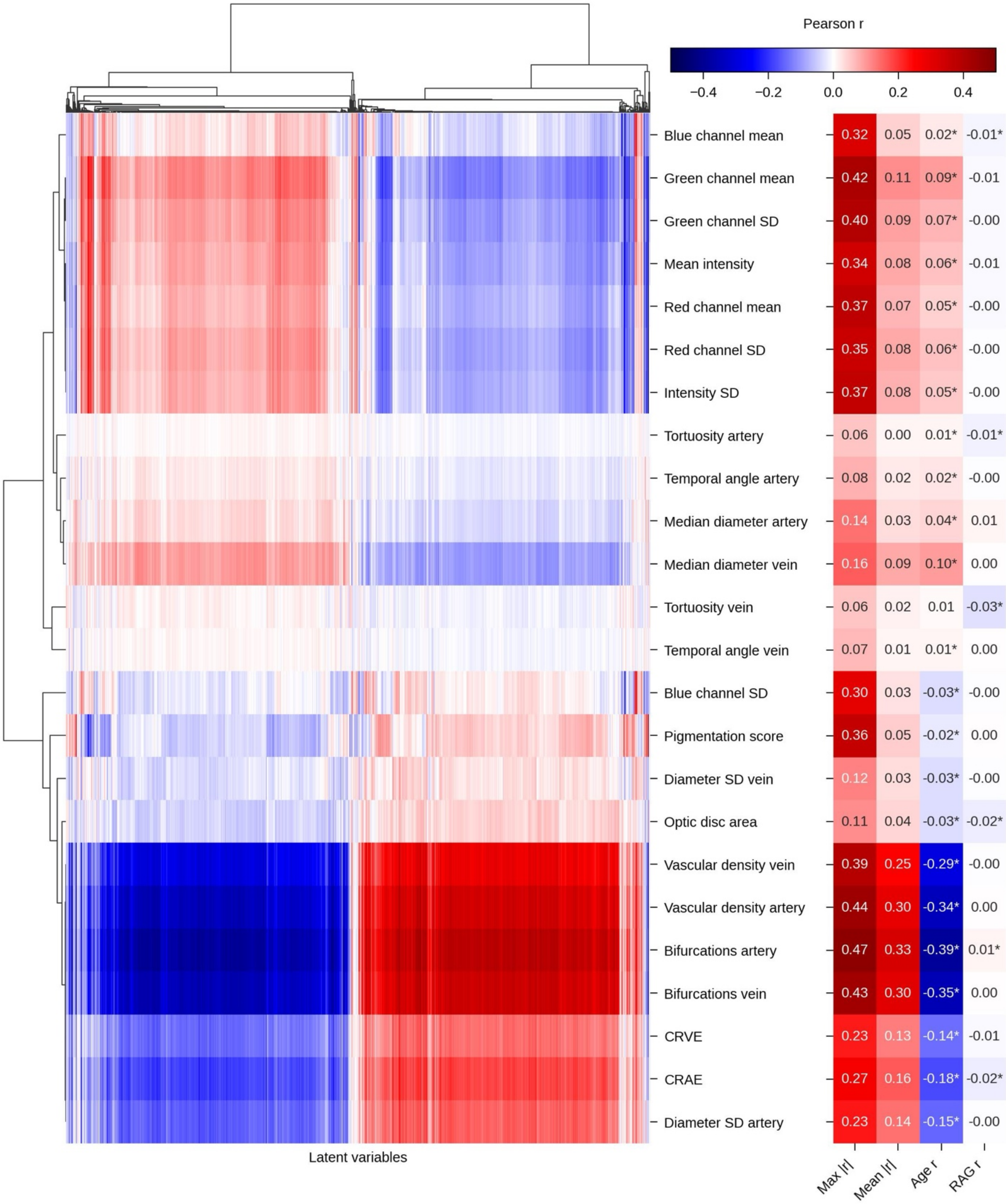

Supplementary Figure 23. **Fold 5 correlation map between the female model's 1024 latent variables and interpretable image features (n=10,772 images).** For every image feature, the maximum and mean absolute correlation with latent variables, the correlation with chronological age and retinal age gap are shown on the right side of the figure. CRAE: central retinal arterial equivalent, CRVE: central retinal venous equivalent, RAG: retinal age gap, SD: standard deviation, *: p<0.05.

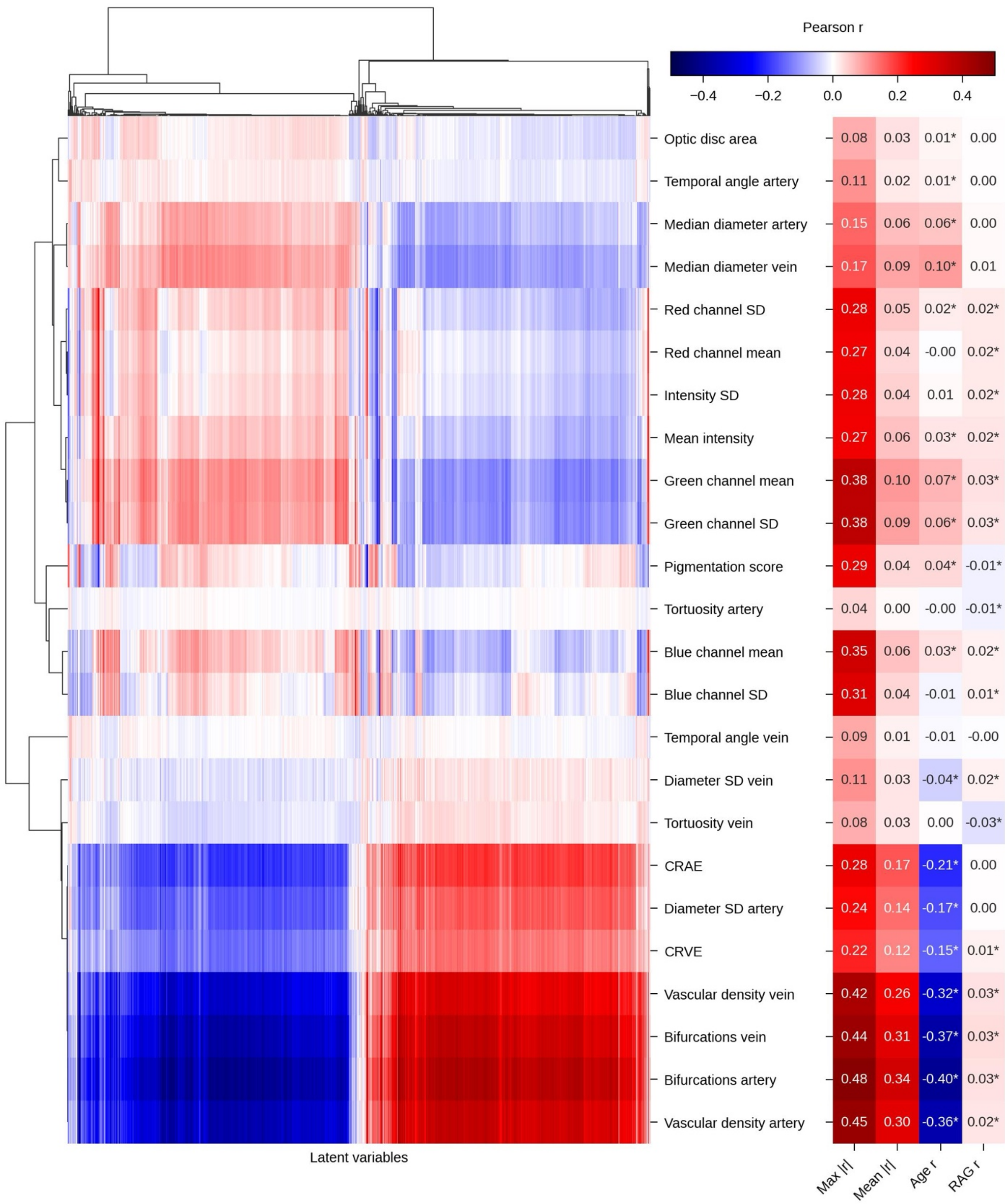

Supplementary Figure 24. **Fold 1 correlation map between the male model's 1024 latent variables and interpretable image features (n=10,791 images).** For every image feature, the maximum and mean absolute correlation with latent variables, the correlation with chronological age and retinal age gap are shown on the right side of the figure. CRAE: central retinal arterial equivalent, CRVE: central retinal venous equivalent, RAG: retinal age gap, SD: standard deviation, *: p<0.05.

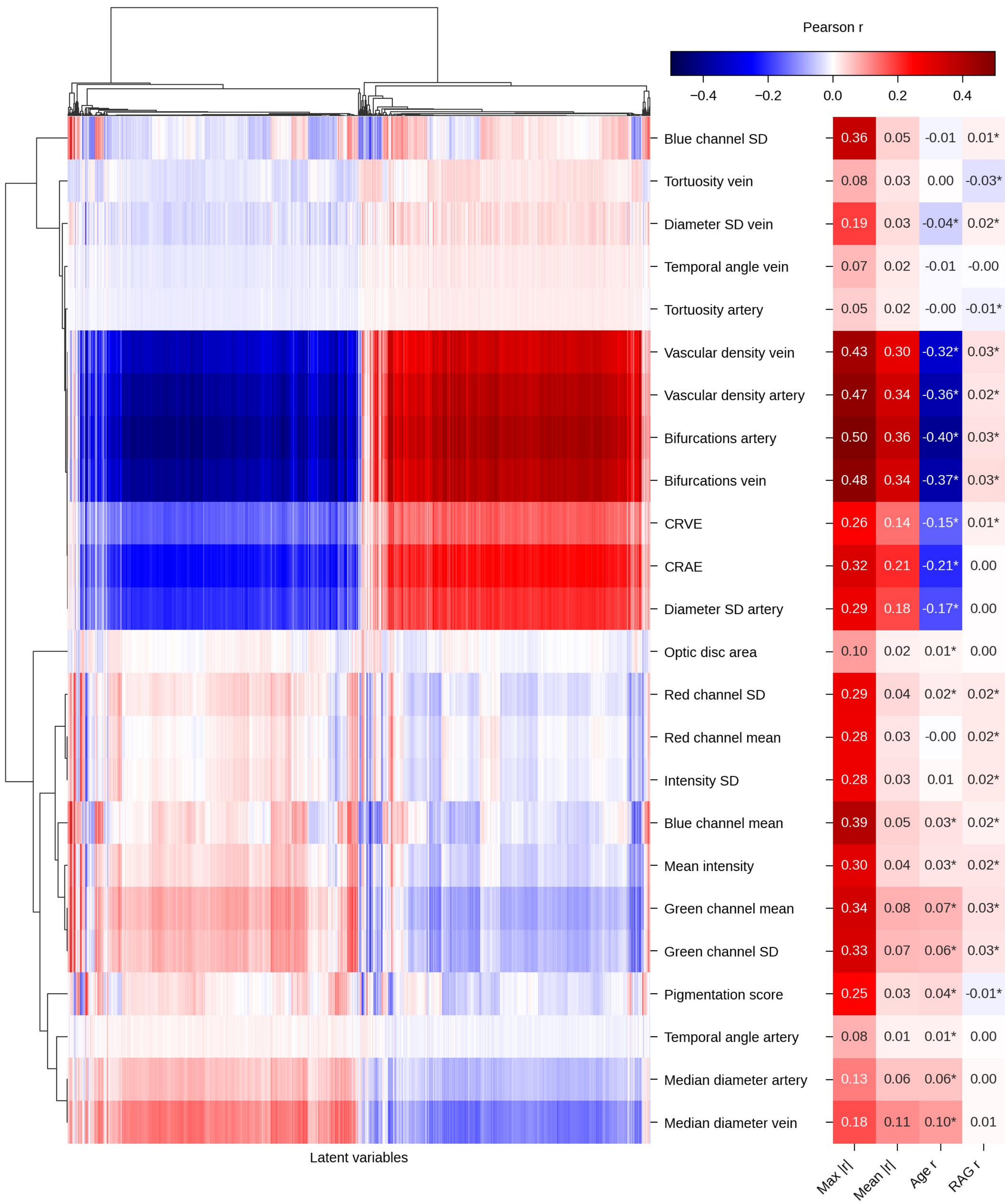

Supplementary Figure 25. **Fold 2 correlation map between the male model's 1024 latent variables and interpretable image features (n=10,749 images).** For every image feature, the maximum and mean absolute correlation with latent variables, the correlation with chronological age and retinal age gap are shown on the right side of the figure. CRAE: central retinal arterial equivalent, CRVE: central retinal venous equivalent, RAG: retinal age gap, SD: standard deviation, *: p<0.05.

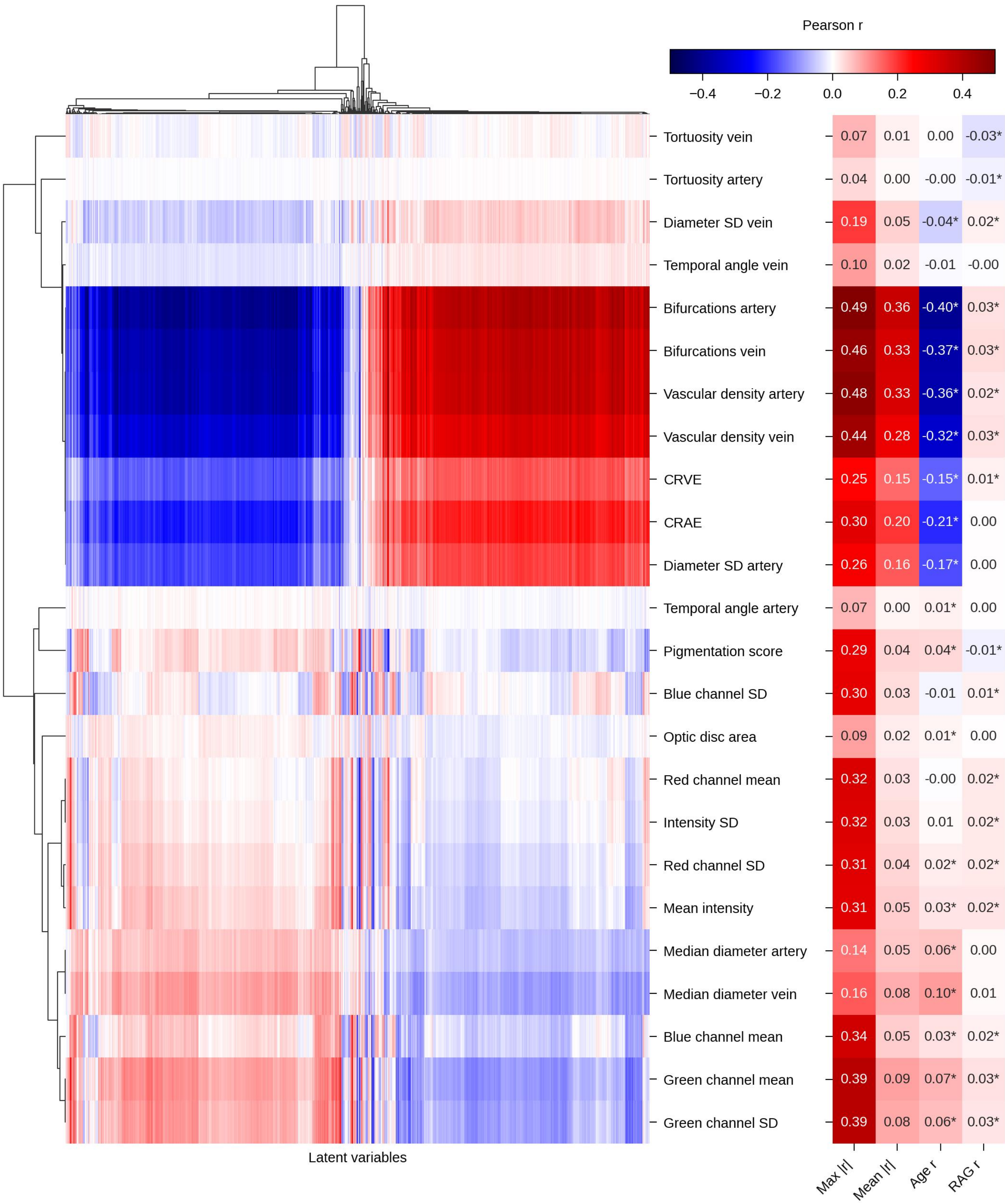

Feature	Max |r|	Mean |r|	Age r	RAG r
Tortuosity vein	0.07	0.01	0.00	-0.03*
Tortuosity artery	0.04	0.00	-0.00	-0.01*
Diameter SD vein	0.19	0.05	-0.04*	0.02*
Temporal angle vein	0.10	0.02	-0.01	-0.00
Bifurcations artery	0.49	0.36	-0.40*	0.03*
Bifurcations vein	0.46	0.33	-0.37*	0.03*
Vascular density artery	0.48	0.33	-0.36*	0.02*
Vascular density vein	0.44	0.28	-0.32*	0.03*
CRVE	0.25	0.15	-0.15*	0.01*
CRAE	0.30	0.20	-0.21*	0.00
Diameter SD artery	0.26	0.16	-0.17*	0.00
Temporal angle artery	0.07	0.00	0.01*	0.00
Pigmentation score	0.29	0.04	0.04*	-0.01*
Blue channel SD	0.30	0.03	-0.01	0.01*
Optic disc area	0.09	0.02	0.01*	0.00
Red channel mean	0.32	0.03	-0.00	0.02*
Intensity SD	0.32	0.03	0.01	0.02*
Red channel SD	0.31	0.04	0.02*	0.02*
Mean intensity	0.31	0.05	0.03*	0.02*
Median diameter artery	0.14	0.05	0.06*	0.00
Median diameter vein	0.16	0.08	0.10*	0.01
Blue channel mean	0.34	0.05	0.03*	0.02*
Green channel mean	0.39	0.09	0.07*	0.03*
Green channel SD	0.39	0.08	0.06*	0.03*

Supplementary Figure 26. **Fold 3 correlation map between the male model's 1024 latent variables and interpretable image features (n=10,638 images).** For every image feature, the maximum and mean absolute correlation with latent variables, the correlation with chronological age and retinal age gap are shown on the right side of the figure. CRAE: central retinal arterial equivalent, CRVE: central retinal venous equivalent, RAG: retinal age gap, SD: standard deviation, *: $p<0.05$.

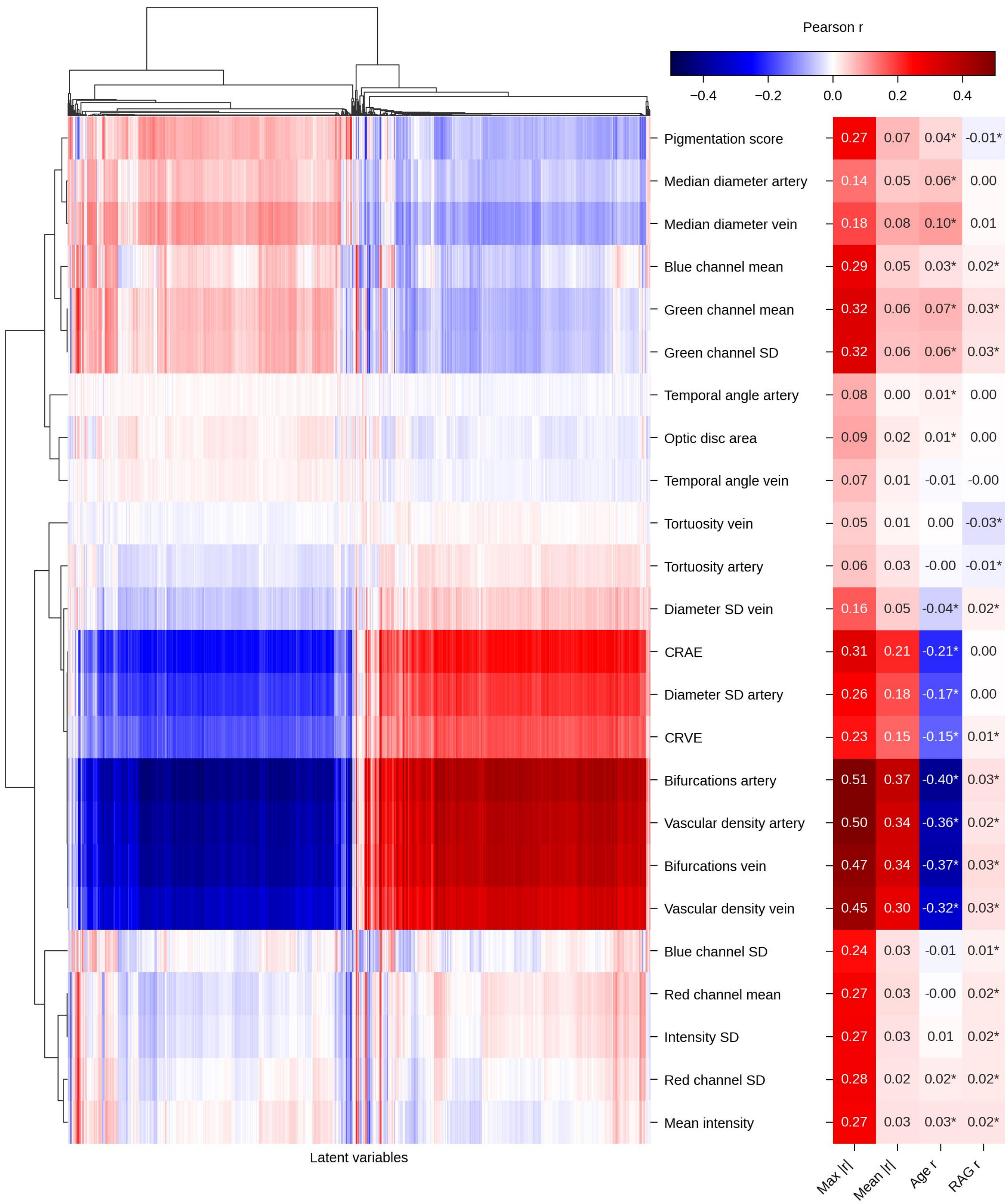

Feature	Max |r|	Mean |r|	Age r	RAG r
Pigmentation score	0.27	0.07	0.04*	-0.01*
Median diameter artery	0.14	0.05	0.06*	0.00
Median diameter vein	0.18	0.08	0.10*	0.01
Blue channel mean	0.29	0.05	0.03*	0.02*
Green channel mean	0.32	0.06	0.07*	0.03*
Green channel SD	0.32	0.06	0.06*	0.03*
Temporal angle artery	0.08	0.00	0.01*	0.00
Optic disc area	0.09	0.02	0.01*	0.00
Temporal angle vein	0.07	0.01	-0.01	-0.00
Tortuosity vein	0.05	0.01	0.00	-0.03*
Tortuosity artery	0.06	0.03	-0.00	-0.01*
Diameter SD vein	0.16	0.05	-0.04*	0.02*
CRAE	0.31	0.21	-0.21*	0.00
Diameter SD artery	0.26	0.18	-0.17*	0.00
CRVE	0.23	0.15	-0.15*	0.01*
Bifurcations artery	0.51	0.37	-0.40*	0.03*
Vascular density artery	0.50	0.34	-0.36*	0.02*
Bifurcations vein	0.47	0.34	-0.37*	0.03*
Vascular density vein	0.45	0.30	-0.32*	0.03*
Blue channel SD	0.24	0.03	-0.01	0.01*
Red channel mean	0.27	0.03	-0.00	0.02*
Intensity SD	0.27	0.03	0.01	0.02*
Red channel SD	0.28	0.02	0.02*	0.02*
Mean intensity	0.27	0.03	0.03*	0.02*

Supplementary Figure 27. **Fold 4 correlation map between the male model's 1024 latent variables and interpretable image features (n=10,859 images).** For every image feature, the maximum and mean absolute correlation with latent variables, the correlation with chronological age and retinal age gap are shown on the right side of the figure. CRAE: central retinal arterial equivalent, CRVE: central retinal venous equivalent, RAG: retinal age gap, SD: standard deviation, *: p<0.05.

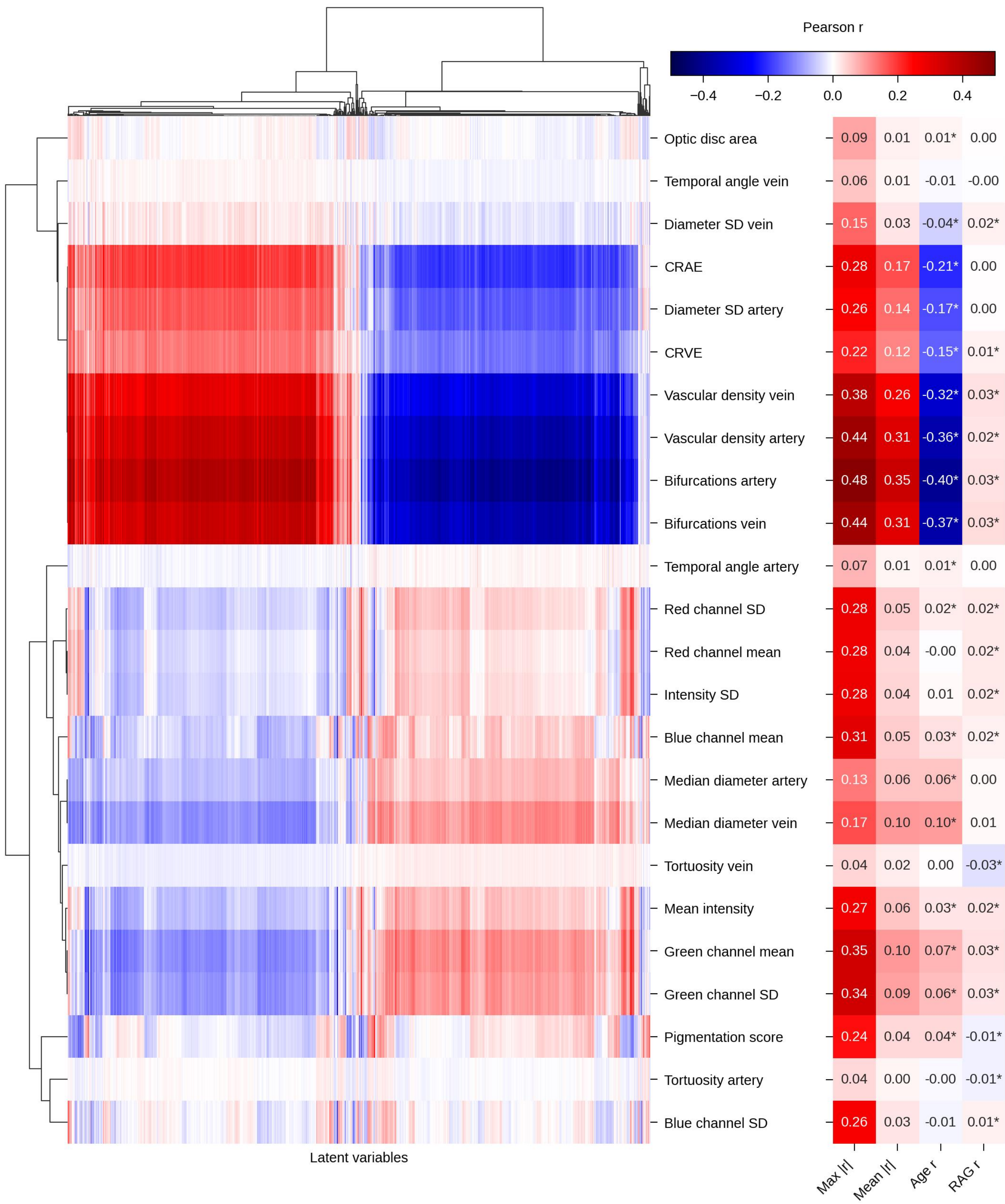

Feature	Max |r|	Mean |r|	Age r	RAG r
Optic disc area	0.09	0.01	0.01*	0.00
Temporal angle vein	0.06	0.01	-0.01	-0.00
Diameter SD vein	0.15	0.03	-0.04*	0.02*
CRAE	0.28	0.17	-0.21*	0.00
Diameter SD artery	0.26	0.14	-0.17*	0.00
CRVE	0.22	0.12	-0.15*	0.01*
Vascular density vein	0.38	0.26	-0.32*	0.03*
Vascular density artery	0.44	0.31	-0.36*	0.02*
Bifurcations artery	0.48	0.35	-0.40*	0.03*
Bifurcations vein	0.44	0.31	-0.37*	0.03*
Temporal angle artery	0.07	0.01	0.01*	0.00
Red channel SD	0.28	0.05	0.02*	0.02*
Red channel mean	0.28	0.04	-0.00	0.02*
Intensity SD	0.28	0.04	0.01	0.02*
Blue channel mean	0.31	0.05	0.03*	0.02*
Median diameter artery	0.13	0.06	0.06*	0.00
Median diameter vein	0.17	0.10	0.10*	0.01
Tortuosity vein	0.04	0.02	0.00	-0.03*
Mean intensity	0.27	0.06	0.03*	0.02*
Green channel mean	0.35	0.10	0.07*	0.03*
Green channel SD	0.34	0.09	0.06*	0.03*
Pigmentation score	0.24	0.04	0.04*	-0.01*
Tortuosity artery	0.04	0.00	-0.00	-0.01*
Blue channel SD	0.26	0.03	-0.01	0.01*

Supplementary Figure 28. **Fold 5 correlation map between the male model's 1024 latent variables and interpretable image features (n=10,751 images).** For every image feature, the maximum and mean absolute correlation with latent variables, the correlation with chronological age and retinal age gap are shown on the right side of the figure. CRAE: central retinal arterial equivalent, CRVE: central retinal venous equivalent, RAG: retinal age gap, SD: standard deviation, *: p<0.05.
