# Supplementary material for: Deep learning aging marker from retinal images unveils sex-specific clinical and genetic signatures
Source: Nat Commun. 2026 Aug 26;17:10185. doi: 10.1038/s41467-026-77102-1 (PMC13612558; doi:10.1038/s41467-026-77102-1)
Supplement: Supplementary file 2 [file 41467_2026_77102_MOESM2_ESM.pdf]

Description of Additional Supplementary Files

Title: Supplementary Data 1

Description: Performance across the five folds of the combined, female-specific, and male-specific models. MAE: mean absolute error, R2: coefficient of determination, RAG: retinal age gap, SD: standard deviation.

Title: Supplementary Data 2

Description: Association between retinal age gap (RAG) and health measurements. Beta estimates were obtained using ordinary least squares models adjusted for age, age-squared, sex, height, height-squared, self-reported ethnic background, 10 genetic principal components, educational attainment, household income, and Townsend Deprivation Index. Chi-square estimates were obtained from likelihood ratio tests comparing nested models with and without RAG. Sex strata effects were compared using a two-sided z-test. P-values are uncorrected. BMI: body mass index, CRP: C-reactive protein, DBP: diastolic blood pressure, HDL: high density lipoprotein, IGF-1: insulin-like growth factor 1, LDL: low density lipoprotein, NLR: neutrophil-to-lymphocyte ratio, SBP: systolic blood pressure, SE: standard error, WHR: waist-to-hip ratio.

Title: Supplementary Data 3

Description: Association between retinal age gap (RAG) and binary health outcomes. Odds ratio (OR) estimates were obtained using logistic regression models adjusted for age, age-squared, sex, height, height-squared, self-reported ethnic background, 10 genetic principal components, educational attainment, household income, and Townsend Deprivation Index. Chi-square estimates were obtained from likelihood ratio tests comparing models with and without RAG. Sex strata effects were compared using a two-sided z-test. P-values are uncorrected. BP: blood pressure, HRT: hormone replacement therapy, SE: standard error.

Title: Supplementary Data 4

Description: Prospective associations between retinal age gap (RAG) and major health outcomes. Hazard ratios (HR) were obtained using Cox proportional hazards regression models adjusted for previous events of the same category (except for death, dementia, and COPD), age, sex, height, self-reported ethnic background, educational attainment, household income, Townsend Deprivation Index, systolic blood pressure, high-density lipoprotein and total cholesterol, smoking status, body mass index, diabetes, and blood pressure medication. Chi-square estimates were obtained from likelihood ratio tests comparing nested models with and without RAG. Changes in model discrimination were evaluated by comparing Harrell's C-index between the nested models using bootstrap resampling (200 iterations). Sex strata effects were compared using a two-sided z-test. P-values are uncorrected. CI: confidence interval, COPD: chronic obstructive pulmonary disease, CVD: cardiovascular disease, IHD: ischemic heart disease, MI: myocardial infarction, NIC: non-ischemic cardiomyopathy.

Title: Supplementary Data 5

Description: Cox model relative contribution of predictors. To quantify the relative importance of each predictor, we conducted a leave-one-out analysis in which each variable was removed from the multivariable model one at a time and the resulting reduction in model fit was calculated from the change in log-likelihood. The corresponding chi-square statistic (χ^2 change) was computed for each variable and expressed as a percentage of the total chi-square across predictors (% χ^2 change), providing an estimate of each variable's relative contribution to the model. P-values are uncorrected. BMI: body mass index, BP: blood pressure, HDL: high-density lipoprotein, RAG: retinal age gap, SBP: systolic blood pressure.

Title: Supplementary Data 6

Description: Genome-wide significant ($p<5x10^{-8}$) independent loci for retinal age gap in the combined sample (n=68,137). Beta estimates were obtained through linear regession adjusted

for age, sex, age-squared, age-by-sex, age-squared-by-sex, height, height-squared, instance, assessment centre, genotype measurement batch, spherical power, spherical power-squared, cylindrical power, cylindrical power-squared, and the first 20 genetic PCs.

Title: Supplementary Data 7

Description: Genome-wide significant ($p<5x10^{-8}$) independent loci for retinal age gap in the female sample (n=30,698). Beta estimates were obtained through linear regession adjusted for age, age-squared, height, height-squared, instance, assessment centre, genotype measurement batch, spherical power, spherical power-squared, cylindrical power, cylindrical power-squared, and the first 20 genetic PCs.

Title: Supplementary Data 8

Description: Genome-wide significant ($p<5x10^{-8}$) independent loci for retinal age gap in the male sample (n=30,958). Beta estimates were obtained through linear regession adjusted for age, age-squared, height, height-squared, instance, assessment centre, genotype measurement batch, spherical power, spherical power-squared, cylindrical power, cylindrical power-squared, and the first 20 genetic PCs.

Title: Supplementary Data 9

Description: Bonferroni-significant ($p<2.61x10^{-6}$) genes associated with retinal age gap in the combined sample.

Title: Supplementary Data 10

Description: Bonferroni-significant ($p<2.51x10^{-6}$) genes associated with retinal age gap in the female sample.

Title: Supplementary Data 11

Description: Bonferroni-significant ($p<2.51x10^{-6}$) genes associated with retinal age gap in the male sample.

Title: Supplementary Data 12

Description: Replication of retinal age gap genome-wide association study in the Rotterdam Study indepedent cohort. Beta estimates were obtained through linear regession adjusted for age, sex, age-squared, age-by-sex, age-squared-by-sex, height, height-squared, spherical power, spherical power-squared, cylindrical power, cylindrical power-squared, and the first 10 genetic PCs. SNPs that were significant in the UKB (see Supplementary Table 6) are sorted by p-value in the replication analysis. P-values are uncorrected; significance is indicated after applying false discovery rate (FDR) correction with an alpha threshold of 0.05.

Title: Supplementary Data 13

Description: Replication of retinal age gap gene analysis in the Rotterdam Study indepedent cohort. Genes that were significant in the UKB (see Supplementary Table 9) are sorted by p-value in the replication analysis. P-values are uncorrected; significance is indicated after applying false discovery rate (FDR) correction with an alpha threshold of 0.05.
