# Supplementary material for: Deep learning aging marker from retinal images unveils sex-specific clinical and genetic signatures
Source: Nat Commun. 2026 Aug 26;17:10185. doi: 10.1038/s41467-026-77102-1 (PMC13612558; doi:10.1038/s41467-026-77102-1)
Supplement: Supplementary file 3 [file 41467_2026_77102_MOESM4_ESM.pdf]

nature portfolio

Corresponding author(s): Olga Trofimova and Sven Bergmann

Last updated by author(s): Jul 30, 2026

Reporting Summary

Nature Portfolio wishes to improve the reproducibility of the work that we publish. This form provides structure for consistency and transparency in reporting. For further information on Nature Portfolio policies, see our Editorial Policies and the Editorial Policy Checklist.

Statistics

For all statistical analyses, confirm that the following items are present in the figure legend, table legend, main text, or Methods section.

n/a	Confirmed	
☐	☒	The exact sample size (n) for each experimental group/condition, given as a discrete number and unit of measurement
☐	☒	A statement on whether measurements were taken from distinct samples or whether the same sample was measured repeatedly
☐	☒	The statistical test(s) used AND whether they are one- or two-sided<br>*Only common tests should be described solely by name; describe more complex techniques in the Methods section.*
☐	☒	A description of all covariates tested
☐	☒	A description of any assumptions or corrections, such as tests of normality and adjustment for multiple comparisons
☐	☒	A full description of the statistical parameters including central tendency (e.g. means) or other basic estimates (e.g. regression coefficient) AND variation (e.g. standard deviation) or associated estimates of uncertainty (e.g. confidence intervals)
☐	☒	For null hypothesis testing, the test statistic (e.g. F, t, r) with confidence intervals, effect sizes, degrees of freedom and P value noted<br>*Give P values as exact values whenever suitable.*
☒	☐	For Bayesian analysis, information on the choice of priors and Markov chain Monte Carlo settings
☐	☒	For hierarchical and complex designs, identification of the appropriate level for tests and full reporting of outcomes
☐	☒	Estimates of effect sizes (e.g. Cohen's d, Pearson's r), indicating how they were calculated

Our web collection on statistics for biologists contains articles on many of the points above.

Software and code

Policy information about availability of computer code

Data collection	n/a
Data analysis	RETFound code is publicly available at https://github.com/rmaphoh/RETFound. The code used to generate the presented results is available at https://github.com/ot710/retinal-age.

For manuscripts utilizing custom algorithms or software that are central to the research but not yet described in published literature, software must be made available to editors and reviewers. We strongly encourage code deposition in a community repository (e.g. GitHub). See the Nature Portfolio guidelines for submitting code & software for further information.

Data

Policy information about availability of data

All manuscripts must include a data availability statement. This statement should provide the following information, where applicable:

- Accession codes, unique identifiers, or web links for publicly available datasets
- A description of any restrictions on data availability
- For clinical datasets or third party data, please ensure that the statement adheres to our policy

The GWAS summary statistics generated in this study are available in the GWAS Catalog (https://www.ebi.ac.uk/gwas/). UK Biobank data are available upon successful application (https://www.ukbiobank.ac.uk/enable-your-research/apply-for-access). Rotterdam Study data can be obtained upon request. Requests should be directed towards the management team of the Rotterdam Study (datamanagement.ergo@erasmusmc.nl), which has a protocol for approving data requests. Because of restrictions based on privacy regulations and informed consent of the participants, data cannot be made freely available in a public repository.

Source data are provided with this paper.

Research involving human participants, their data, or biological material

Policy information about studies with human participants or human data. See also policy information about sex, gender (identity/presentation), and sexual orientation and race, ethnicity and racism.

Reporting on sex and gender	Sex-specific analyses were performed. The terms "sex", "female", and "male" are consistently used in the text and figures.
Reporting on race, ethnicity, or other socially relevant groupings	Ethnic background was self-reported and used as a covariate. We also used the first genetic principal components (PCs) as measures of ethnic background. We controlled separately for socioeconomic status (education, income, Townsend deprivation index) and for ethnic background.
Population characteristics	Participants were between 39 and 79 years old, from the general population of the UK and Rotterdam.
Recruitment	Participants were recruited from the general population and were shown to be of higher socioeconomic status and overall health status than the general population they were drawn from.
Ethics oversight	UK Biobank: National Information Governance Board for Health and Social Care and the NHS North West Multicenter Research Ethics Committee<br>Rotterdam Study: Medical Ethics Committee of the Erasmus MC (registration number MEC 02.1015) and by the Dutch Ministry of Health, Welfare and Sport (Population Screening Act WBO, license number 1071272-159521-PG).

Note that full information on the approval of the study protocol must also be provided in the manuscript.

Field-specific reporting

Please select the one below that is the best fit for your research. If you are not sure, read the appropriate sections before making your selection.

[x] Life sciences [ ] Behavioural & social sciences [ ] Ecological, evolutionary & environmental sciences

For a reference copy of the document with all sections, see nature.com/documents/nr-reporting-summary-flat.pdf

Life sciences study design

All studies must disclose on these points even when the disclosure is negative.

Sample size	Sample size was based on availability of retinal color fundus imaging data in respective cohort studies.
Data exclusions	We excluded 25% of images with worst quality. This threshold was pre-defined based on previous studies on data quality control.
Replication	We validated the model developed using UK Biobank data in an independent dataset from the Rotterdam Study. We also replicated genetic associations found in the UK Biobank. Genetic associations partially replicated (24% of SNPs and 35% of genes significant in the replication cohort). The non-significant signals aligned in effect size with the original analysis.
Randomization	Participants were not allocated to groups. Both train and test sets included participants from the whole cohort, without selection criteria. Sex-specific analyses were done on age-matched sub-cohorts of females and males, to avoid potential bias stemming from differences in age distributions between sexes.
Blinding	Blinding was not relevant to this study, since data was collected as part of large population-based cohort studies.

Reporting for specific materials, systems and methods

We require information from authors about some types of materials, experimental systems and methods used in many studies. Here, indicate whether each material, system or method listed is relevant to your study. If you are not sure if a list item applies to your research, read the appropriate section before selecting a response.

Materials & experimental systems

n/a	Involved in the study	
x		Antibodies
x		Eukaryotic cell lines
x		Palaeontology and archaeology
x		Animals and other organisms
	x	Clinical data
x		Dual use research of concern
x		Plants

Methods

n/a	Involved in the study	
x		ChIP-seq
x		Flow cytometry
x		MRI-based neuroimaging

Clinical data

Policy information about clinical studies

All manuscripts should comply with the ICMJE guidelines for publication of clinical research and a completed CONSORT checklist must be included with all submissions.

Clinical trial registration	n/a
Study protocol	n/a
Data collection	Medical diagnoses were collected from participants' medical records, in accordance with the signed informed consent.
Outcomes	n/a

Plants

Seed stocks	n/a
Novel plant genotypes	n/a
Authentication	n/a
