# Supplementary material for: Deep learning aging marker from retinal images unveils sex-specific clinical and genetic signatures
Source: Nat Commun. 2026 Aug 26;17:10185. doi: 10.1038/s41467-026-77102-1 (PMC13612558; doi:10.1038/s41467-026-77102-1)
Supplement: Supplementary file 4 [file 41467_2026_77102_MOESM5_ESM.pdf]

nature portfolio

Peer Review File

Deep learning aging marker from retinal images unveils sex-specific clinical and genetic signatures

Corresponding Author: Dr Olga Trofimova

Parts of this Peer Review File have been redacted as indicated to remove third-party material.

This file contains all reviewer reports in order by version, followed by all author rebuttals in order by version.

Version 0:

Reviewer comments:

Reviewer #1

(Remarks to the Author)
This study presents an AI-based approach to quantify retinal ageing using color fundus images from the UK Biobank. By fine-tuning the RETFound foundation model to predict chronological age, the authors define a “retinal age gap” (RAG)—the difference between predicted and actual age—and show that this metric correlates with systemic traits such as cardiometabolic health, inflammation, cognition, and mortality. Through sex-stratified analyses, they report broadly similar model performance between males and females but distinct biological patterns, with metabolic associations predominating in males and vascular features in females.

Overall, this is a timely and interesting contribution intersecting artificial intelligence, imaging, and ageing research. However, given the emphasis on sex differences, two points should be considered:
1- Genetic related sex differences: It is surprising that no genome-wide significant associations mapped to the X chromosome. The authors should clarify whether in their GWAS analysis any sub-threshold associations were observed, or if their results could also be related to the reference panel they used. More generally, known X-linked retinal genes (e.g., RS1, RPGR, CHM, CACNA1F) are associated with distinct retinal imaging phenotypes visible on OCT or fundus images, it would be useful to know whether variants in or near these loci showed any association with the retinal age gap phenotype. A recent study mapped retinal traits to the X chromosome (e.g., Jackson et al., Nat. Commun., 2025), the paper should be cited and their results discussed.

2- Hormonal related sex differences: Given the wide age range of participants (40–80 years) and the strong physiological and hormonal changes associated during this period in woman, it would be informative to explore whether the retinal age gap differs if women are analysis with age stratification (for example below and above 55).This would help to understand if vascular changes after menopause might mimic accelerated ageing signals in the retina, creating an apparent sex bias unrelated to genetics, and which would appear when women are analysis as one group.

Expanding the section biological interpretation of the sex-specific findings would strengthen the manuscript and provide a clearer context for the observed sex effects.

Reviewer #2

(Remarks to the Author)
This manuscript presents a large-scale analysis of retinal age prediction using a fine-tuned foundation model (RETFound) on UK Biobank and Rotterdam Study datasets. The study is comprehensive, covering model benchmarking, sex-specific analyses, and downstream clinical associations. The scale of the dataset and the integration of multimodal downstream analyses make the work impactful.

There are several major conceptual concerns regarding the definition, validity, and clinical usefulness of the proposed retinal age gap (RAG) that must be addressed. In particular, the manuscript should clarify (1) what biological construct RAG is actually measuring, (2) whether it is appropriate to train exclusively on nominally “normal” images, and (3) whether RAG

adds predictive value beyond chronological age.

Training exclusively on “normal” retinas may introduce bias and lead to unrealistic ground-truth assumptions. As outlined in the Methods, the model is trained to predict chronological age using UK Biobank fundus images that passed QC filtering. This process preferentially excludes diseased or poor-quality images. But individuals with early or subclinical systemic or ocular disease (e.g., early vascular dysfunction, preclinical AMD, diabetes) may still meet QC criteria. As a result, their retinal phenotype may already deviate from a healthy baseline. The model, therefore, implicitly treats chronological age as a clean ground truth, despite the likelihood that biological and pathological changes may precede clinical diagnosis. This mislabeling could bias the learned age signal.

The paper needs to compare the proposed method with chronological age and standard risk factors, showing the clinical utility of RAG. The manuscript shows that RAG is statistically associated with mortality and multiple incident diseases (Fig. 3). However, it remains uncertain whether RAG provides incremental prognostic value beyond chronological age and conventional clinical factors. Several of the authors’ own findings suggest that RAG may not offer additional predictive utility. First, Chronological age itself is a strong predictor of mortality. Second, many vascular features (e.g., vessel density, branching metrics) correlate with both RAG and chronological age in similar ways (Fig. 6), suggesting that RAG may largely reflect age-related vasculopathy rather than a distinct biological construct. Third, although the Cox models adjust for major risk factors, the manuscript does not present whether adding RAG improves model performance (e.g., change in C-index), how RAG compares directly with chronological age, or whether it enhances risk classification relative to established models. Without these key comparisons, it is difficult to assess the clinical relevance of RAG or determine whether it meaningfully improves risk prediction rather than recapitulating variance already explained by chronological age.

Please clarify whether Cole’s correction was applied uniformly across all downstream analyses (Fig. S1) and whether any sex-specific residual bias persists (e.g., Fig. S2b).

The manuscript reports that male retinas appear “younger,” but the proposed explanations (e.g., differences in height or ocular dimensions) are not strongly supported. Additional empirical evidence or supplementary analyses would be needed to justify sex differences in retinal age prediction.

Although saliency and attention maps highlight the macula, optic disc, and vessels, such methods do not necessarily reveal the underlying biological mechanisms.

The authors should discuss how differences in phenotype definitions, imaging protocols, population characteristics, and genetic replication variability across datasets may contribute to discrepancies in results. Additionally, please clarify whether these cohort-level differences could affect the stability or generalizability of the retinal age model, and how future work should account for such dataset-related inconsistencies.

Version 1:

Reviewer comments:

Reviewer #1

(Remarks to the Author)
The authors have answered my questions.

Reviewer #2

(Remarks to the Author)
Thanks for having a chance to go through the revised version and the rebuttal. Overall, I still have reservations regarding approaches that train age-prediction models on broadly “normal” images and subsequently interpret the residual (RAG) as a biologically meaningful signal. In line with Reviewer #2, I outline several concerns below.

1. Unclear biological interpretation of RAG

RAG is defined as the residual from chronological age, which is a mathematical construct rather than a biologically validated measure. Although RAG demonstrates associations with multiple outcomes, these relationships do not elucidate the underlying biological processes it reflects (e.g., vascular aging versus heterogeneous or nonspecific effects). Moreover, its weak correlation with interpretable retinal features raises the possibility that RAG primarily captures residual variation rather than a well-defined biological signal.

2. Concerns regarding the supervision signal and the training objective

The authors clarify that the dataset includes participants with diagnosed systemic and ocular diseases, which is helpful. However, this raises a more fundamental concern regarding the training objective. The model is trained to predict chronological age using a mean absolute error (MAE) loss. Under this setup, individuals with disease-related retinal changes are still assigned their chronological age as the ground truth. As a result, the model is encouraged to fit these cases as accurately as possible, which would tend to drive their predicted age and thus their RAG toward zero. This creates a conceptual tension: if disease-related deviations are already absorbed into the training target, it is unclear how RAG can later be interpreted as capturing biologically meaningful deviations from normal aging. In other words, the current framework may implicitly treat disease-associated variation as part of the "normal" aging signal during training, rather than learning a clean aging trajectory. This raises concerns about whether RAG reflects true biological aging differences or artifacts of the training setup.

3. Limited incremental clinical utility

While RAG is statistically associated with multiple outcomes, its practical predictive value appears modest. Likelihood ratio tests are consistently significant, but this is expected in large datasets and does not necessarily indicate meaningful improvement in prediction. Gains in discrimination (e.g., C-index) are small and often not statistically significant, as acknowledged by the authors. Furthermore, leave-one-out analyses suggest that chronological age remains the dominant contributor to model performance, with RAG providing only incremental information.

Taken together, these findings suggest that RAG captures some residual signal; however, its added value beyond established predictors, particularly chronological age, appears limited. It therefore remains unclear whether RAG meaningfully enhances clinical risk stratification or largely recapitulates information already contained in existing variables.

Reviewer #3

(Remarks to the Author)
The authors have made a careful and useful revision in response to the conceptual issues raised by Reviewer #2. Reframing the retinal age gap (RAG) as a deviation from a reference population's retinal ageing trajectory, rather than as a direct or absolute measure of biological age, is an important and appropriate correction. I agree that the residual-age framework is not conceptually invalid. Chronological age is an imperfect but practical supervisory signal, provided that the resulting residual is interpreted strictly as an age-orthogonal retinal imaging phenotype.

The additional analyses and clarifications substantially strengthen the manuscript. These include the clarification of age-bias correction, likelihood-ratio tests, C-index comparisons, leave-one-out contribution analyses, and stratification of female participants by menopausal status and age. These additions address several of the key concerns raised by Reviewer #2, particularly whether RAG simply recapitulates chronological age and whether the sex-specific findings have a plausible biological interpretation.

However, the conceptual framing still remains too strong in several places and should be further qualified.

1. Clinical utility and effect sizes

The data support RAG as a promising candidate retinal imaging biomarker associated with ageing-related clinical traits. They do not establish RAG as a clinically useful risk prediction tool or as ready for personalized risk assessment. The improvements in model discrimination, including C-index changes, appear limited for several outcomes, and the effect sizes are generally modest. The manuscript should therefore soften statements that imply established clinical utility.

2. Biological specificity and the OCA2 signal

The biological specificity of the RAG signal warrants a more critical discussion. The prominent GWAS signal involving OCA2 is particularly noteworthy, given its role in ocular pigmentation. Together with the latent-variable correlations with RGB and intensity-related features, this raises a plausible concern that part of the model-derived retinal age signal may reflect fundus pigmentation, color distribution, image brightness, or acquisition-related characteristics, rather than ageing biology alone. This does not invalidate the findings, but it is directly relevant to the construct validity and generalizability of RAG. The Limitations section should clearly state that genetic loci associated with RAG cannot be assumed to reflect ageing-related pathways alone.

3. Distinguishing the age-prediction model from the residual RAG phenotype

The manuscript should more clearly distinguish the age-prediction model from the residual RAG phenotype. The saliency and attention analyses suggest that the age-prediction model attends to the macula, optic disc, and vascular regions, among other features. In contrast, the updated feature-correlation analyses suggest that the residual RAG phenotype is only weakly

explained by the predefined vascular and color features examined. These findings are not contradictory, because they refer to different levels of analysis. However, the text should avoid implying that RAG is independent of vascular ageing in general. A more cautious interpretation would be that RAG is not strongly explained by the explicit vascular and color features examined here, while more complex, nonlinear, or spatially localized vascular contributions may remain.

4. Nuance in the sex-specific interpretation

The sex-specific narrative also needs refinement. Stating simply that male retinas appear younger may obscure the pattern shown in the revised analyses, where the observed difference appears to be driven at least partly by higher RAG in older or postmenopausal females, with males lying between younger and older female strata. The authors should frame this as a life-stage-dependent pattern rather than a simple binary male-versus-female difference. If the authors wish to maintain a strong sex-difference interpretation, stratifying males by the same age threshold or formally testing sex-by-age-group interactions would be helpful.

5. Methodological transparency relevant to interpretation

Several methodological points should be clarified because they affect how the residual phenotype should be interpreted. The manuscript should explicitly state which RAG measure was used for each downstream analysis, for example whether it was based on a single eye, both-eye average, or all available images at the first imaging instance. It should also clarify whether combined-sample and sex-stratified analyses used RAG derived from the combined model or from sex-specific models. Finally, the description of covariate adjustment, including height and height squared, should be made consistent across cross-sectional and survival analyses.

In summary, this is a solid exploratory study of a retinal imaging biomarker. The authors have substantially addressed Reviewer #2’s conceptual concerns, but the manuscript should more clearly frame RAG as a candidate age-orthogonal retinal imaging phenotype, not as a direct measure of biological ageing or an established clinically useful risk prediction tool. With this more cautious framing, the conceptual basis of the study would be sound and appropriately scoped.

Version 2:

Reviewer comments:

Reviewer #2

(Remarks to the Author)
Thank you to the authors for addressing the concerns raised during the review process. I appreciate the effort that went into revising the manuscript and clarifying several points.

That said, I still have reservations about the proposed approach and its overall soundness. While the revisions have improved the paper, they have not fully resolved my concerns. Given that the reviews will be public, I am not comfortable stating that my concerns have been satisfactorily addressed when I still have substantive reservations.

At this stage, I do not believe that further rounds of review would be productive, as I do not expect my assessment to change substantially. I will therefore defer to the editor regarding the final decision, taking into account the other reviewers’ assessments and the journal’s standards.

Reviewer #3

(Remarks to the Author)
I have reviewed the revised manuscript and the authors' responses. In my view, the authors have substantially addressed the conceptual concerns from the previous round. The reframing of RAG as an age-orthogonal retinal imaging-derived phenotype, rather than as a direct or absolute measure of biological age, is a particularly important correction. I also appreciate that the manuscript now explicitly acknowledges that disease-related retinal features may be incorporated into the learned age representation, that OCA2 and pigmentation- or brightness-related image features may contribute to part of the RAG signal, and that genetic loci associated with RAG should not be interpreted exclusively as ageing-related biological pathways.

The distinction between the age-prediction model and the residual RAG phenotype is also clearer now, with the interpretation of the model's saliency/attention patterns separated from that of the residual phenotype. I found the refinement of the sex-specific interpretation persuasive: framing the findings as a life-stage-dependent pattern tied to menopausal status and age, rather than a simple male-versus-female difference, fits the data better. The clarifications on eye-level versus subject-level predictions, age-bias correction, combined versus sex-specific RAG measures, and the differing covariate adjustment between cross-sectional and survival analyses are all helpful as well.

The major conceptual issues are therefore largely resolved, and the remaining points are minor—addressable through textual and technical revision. I recommend minor revision.

1. Please further soften the wording around biological ageing and clinical utility in the Introduction and conclusion. The

Abstract and much of the Discussion now read appropriately, but the Introduction still contains phrases such as "promising marker of biological aging" and "risk stratification in both clinical and population-level health settings," and the stated aim still refers to "assess their clinical utility." Given the modest effect sizes and the limited improvements in discrimination, more cautious wording would be preferable—for example, "candidate retinal imaging-derived aging-associated phenotype" and "potential relevance for future risk stratification research."

2. I would suggest softening the use of "predicts" in the survival-analysis section. The prospective associations are important, but the results do not establish RAG as a clinically useful prediction tool. The heading "Retinal age gap predicts mortality and incident disease" might be changed to "Retinal age gap is prospectively associated with mortality and incident disease," which would better align with the cautious framing adopted elsewhere.

3. Please check the notation and algebraic specification of the age-bias correction formula. In Methods, p.15, lines 536–537, predicted age is said to be regressed on chronological age to estimate the slope and intercept, but both quantities appear to be denoted by the same symbol. The subsequent statement that predicted age was adjusted by "subtracting and dividing by" is consequently ambiguous. Please state the fitted linear model and the correction formula explicitly.

4. Please temper the final statement on precision medicine. The sex-stratified and menopause-stratified analyses are valuable, but the current data do not establish direct clinical implementation. "Advancing their application in precision medicine" could be softened to something like "informing future biomarker development" or "guiding future studies of sex-specific ageing biomarkers."

These remaining points are minor, concerning wording and technical clarity rather than substance. From my perspective, no additional major analyses are needed.

Open Access This Peer Review File is licensed under a Creative Commons Attribution 4.0 International License, which permits use, sharing, adaptation, distribution and reproduction in any medium or format, as long as you give appropriate credit to the original author(s) and the source, provide a link to the Creative Commons license, and indicate if changes were made.
In cases where reviewers are anonymous, credit should be given to 'Anonymous Referee' and the source.
The images or other third party material in this Peer Review File are included in the article's Creative Commons license, unless indicated otherwise in a credit line to the material. If material is not included in the article's Creative Commons license and your intended use is not permitted by statutory regulation or exceeds the permitted use, you will need to obtain permission directly from the copyright holder.
To view a copy of this license, visit https://creativecommons.org/licenses/by/4.0/

Reviewer #1 (Remarks to the Author):

This study presents an AI-based approach to quantify retinal ageing using color fundus images from the UK Biobank. By fine-tuning the RETFound foundation model to predict chronological age, the authors define a "retinal age gap" (RAG)—the difference between predicted and actual age—and show that this metric correlates with systemic traits such as cardiometabolic health, inflammation, cognition, and mortality. Through sex-stratified analyses, they report broadly similar model performance between males and females but distinct biological patterns, with metabolic associations predominating in males and vascular features in females.

> We thank the Reviewer for their careful reading of our manuscript and for their thoughtful and constructive comments. We appreciate their positive assessment of the timeliness and interdisciplinary nature of our work, and we are grateful for the opportunity to further clarify and expand on the sex-specific findings.

Overall, this is a timely and interesting contribution intersecting artificial intelligence, imaging, and ageing research. However, given the emphasis on sex differences, two points should be considered:
1- Genetic related sex differences: It is surprising that no genome-wide significant associations mapped to the X chromosome. The authors should clarify whether in their GWAS analysis any sub-threshold associations were observed, or if their results could also be related to the reference panel they used. More generally, known X-linked retinal genes (e.g., RS1, RPGR, CHM, CACNA1F) are associated with distinct retinal imaging phenotypes visible on OCT or fundus images, it would be useful to know whether variants in or near these loci showed any association with the retinal age gap phenotype. A recent study mapped retinal traits to the X chromosome (e.g., Jackson et al., Nat. Commun., 2025), the paper should be cited and their results discussed.

> **Response:**
> We agree that, given our emphasis on sex differences, a careful examination of X chromosome associations is highly relevant. In our genome-wide analyses, we did not observe any genome-wide significant associations on the X chromosome in either the combined or sex-stratified GWAS. In the combined-sex analysis, there was a single sub-threshold association (rs1494435, $p = 2.76 \times 10^{-7}$), mapping to the lncRNA RP11-298A8.2; however, this variant and locus have not previously been linked to any phenotype, and no additional supporting signal was observed in the region.
>
> We specifically examined whether variants in or near known X-linked retinal disease genes highlighted by the Reviewer (e.g., *RS1*, *RPGR*, *CHM*, *CACNA1F*) showed any association with retinal age gap (RAG), even at sub-threshold levels. We did not detect evidence of association at these loci. These genes are primarily implicated in rare, often early-onset retinopathies with distinct structural phenotypes. In contrast, our model was trained in a population-based cohort aged 40–80 years and is therefore more likely to capture common, late-onset and aging-related changes in the retina. It is plausible that structural abnormalities driven by the mentioned X chromosome genes are either underrepresented in the UK Biobank

cohort or not major contributors to inter-individual variation in retinal aging within this age range.

We also thank the Reviewer for pointing out the recent study by Jackson et al. (Nat. Commun., 2025), which reported four X chromosome loci associated with OCT-derived retinal thickness traits. We have now cited and discussed this work in the revised manuscript. Notably, we did not observe even sub-threshold associations at the *SHROOM2* or *EFNB1* loci identified in that study. This may reflect important modality-specific differences: genetic signals for features derived from OCT and color fundus images have been shown to differ substantially, as these imaging techniques capture distinct anatomical and physiological properties of the retina. Indeed, retinal thickness measures may reflect structural variation not readily visible in color fundus photographs.

Finally, regarding technical considerations, we used UK10K-based reference panels for the autosomes, as recommended for UK Biobank, and constructed analogous panels from UK Biobank data for the X chromosome. Therefore, we consider it unlikely that the absence of significant X chromosome findings is attributable to imputation or reference panel limitations.

We have incorporated a brief discussion of these points in the revised manuscript.

Revised manuscript text (Discussion): Notably, despite prior reports of X-linked genes underlying retinal diseases and OCT-derived retinal thickness traits (De Silva et al., 2021; Jackson et al., 2025), we did not observe genome-wide significant associations on the X chromosome for RAG. This suggests that RAG primarily reflects common, age-related features visible on color fundus imaging, rather than retinal thickness variation or structural changes characteristic of rare, early-onset retinopathies.

2- Hormonal related sex differences: Given the wide age range of participants (40–80 years) and the strong physiological and hormonal changes associated during this period in woman, it would be informative to explore whether the retinal age gap differs if women are analysis with age stratification (for example below and above 55).This would help to understand if vascular changes after menopause might mimic accelerated ageing signals in the retina, creating an apparent sex bias unrelated to genetics, and which would appear when women are analysis as one group.

Response:
We thank the Reviewer for this insightful suggestion. We agree that the age range of participants, together with the substantial physiological and hormonal changes occurring around menopause, could influence retinal aging signals and potentially contribute to the observed sex differences. Following the Reviewer's recommendation, we conducted additional analyses in females stratified by menopausal status and, separately, by age (<55 vs ≥55 years).

These analyses revealed that RAG differed across female life stages: younger/premenopausal females had lower RAG on average, whereas older/postmenopausal females had higher RAG, with males positioned between these two groups. Moreover,

associations between RAG and cardiometabolic biomarkers were generally stronger in postmenopausal/older females and more closely resembled those observed in males, whereas several traits, including ocular diseases, cancer and hypertension, showed stronger associations in younger/premenopausal females. These findings suggest that the apparent sex differences in retinal age may partly reflect life-stage–dependent biological processes, with females showing relative protection before menopause followed by a convergence toward male-like cardiometabolic risk profiles afterward.

Overall, we found this analysis highly informative and believe it substantially clarifies the biological interpretation of the sex-specific findings. We have therefore incorporated these additional analyses into the Abstract, Results, Discussion, and Methods sections of the revised manuscript and provided detailed results in the Supplementary Figures.

Revised manuscript text (Abstract): Retinal fundus images offer a non-invasive window into systemic aging. Here, we fine-tuned a foundation model (RETFound) to predict chronological age from color fundus images in 71,343 participants from the UK Biobank, achieving a mean absolute error of 2.85 years. The resulting retinal age gap, i.e. the difference between predicted and chronological age, was associated with cardiometabolic traits, inflammation, cognitive performance, all-cause mortality, dementia, cancer, and incident cardiovascular disease. Genome-wide analyses identified genes related to longevity, metabolism, neurodegeneration, and age-related eye diseases. Sex-stratified models revealed consistent performance but divergent biological signatures: males had younger-appearing retinas and stronger links to metabolic syndrome, while in females, both model attention and genetic associations pointed to a greater involvement of retinal vasculature. Additional analyses indicated that retinal aging patterns in females vary across the menopausal transition, with older/postmenopausal females showing retinal age profiles and clinical associations that more closely resemble those observed in males. Our study positions retinal aging as a biologically meaningful and sex-sensitive biomarker that can support more personalized approaches to risk assessment and aging-related healthcare.

Revised manuscript text (Results): Stratified analyses in females revealed that RAG was higher in postmenopausal than premenopausal females ($\Delta = 0.60$, $t = 10.7$, $p = 1e\text{-}26$, $d = 0.153$), with male RAG positioned in between these groups (see **Fig. S3a**). Similar patterns were observed when stratifying females by age (<55 vs ≥55 years; **Fig. S3b**), with older females exhibiting higher RAG than younger females despite the orthogonalization of RAG to chronological age.

In the RS, we observed a similar pattern: males had lower mean RAG than females, indicating younger-appearing retinas ($\Delta = 0.37$, $t = 2.9$, $p = 0.004$, Cohen's $d = 0.085$). When height was regressed out of the RAG, the sex difference was again reduced but not removed ($\Delta = 0.30$, $t = 2.4$, $p = 0.015$, Cohen's $d = 0.071$). Cross-application of sex-specific models yielded consistent results, with male retinas evaluated using the female-trained model showing a mean RAG of -0.81, and female retinas evaluated using the male-trained model showing a mean RAG of 0.67. When stratifying females by age (<55 vs ≥55 years), RAG was higher in

older females than in younger females (Δ = 0.40, t = 2.29, p = 0.02, d = 0.09) or males (Δ = 0.51, t = 3.59, p < 0.001, d = 0.12), while there was no significant difference between younger females and males (Δ = 0.11, t = 0.63, p = 0.53, d = 0.02). These findings replicate the UKB results and further support the observation that retinal age predictions tended to estimate postmenopausal/older female retinas as appearing older than retinas of males and premenopausal/younger females.

(...)

To further explore potential hormonal influences, we repeated association analyses in females stratified by menopausal status and, separately, by age (<55 vs ≥55 years) (**Fig. S4–S5** and **Tables S2–S3**). Overall, associations between higher RAG and adverse cardiometabolic and inflammatory biomarkers were stronger in postmenopausal/older females than in premenopausal/younger females, rendering the former more similar to males in effect size patterns. Notable exceptions included sleep duration, which was associated with lower RAG only in premenopausal/younger females, and cataract, macular degeneration, cancer, and hypertension, for which associations were stronger in premenopausal/younger females. Interestingly, triglycerides, LDL, and total cholesterol were associated with lower RAG specifically in postmenopausal/older females, a pattern not observed in premenopausal/younger females or in males.

(...)

When stratifying females by menopausal status or age group (**Fig. S6**), most incident disease associations were driven by postmenopausal/older females, whereas associations in premenopausal/younger females were generally weaker and often not statistically significant, possibly reflecting both biological differences and lower event counts in the younger stratum. An exception was all-cause mortality, which showed significant effect in both younger and older females.

Revised manuscript text (Discussion):

We trained sex-specific models and found differences in RAG and its associations with clinical outcomes and genetic variants between females and males, with additional analyses suggesting that retinal aging patterns in females vary across the menopausal transition.

(...)

Our stratified analyses suggest that hormonal and life-stage factors may also contribute. Specifically, RAG was lower in younger or premenopausal females but higher in older or postmenopausal females, placing males between these two groups. This pattern parallels epidemiological observations in which females experience lower cardiometabolic risk prior to menopause, followed by a marked increase afterward, including a rise in CVD incidence (Fasero & Coronado, 2025). Such transitions may influence retinal vascular and metabolic features that the model captures as aging-related signals.

Associations between RAG and cardiometabolic risk factors were stronger in males, consistent with the higher prevalence of metabolic syndrome and related conditions in men during midlife (Noubiap et al., 2025). However, when females were stratified by menopausal status or age, the pattern of associations in older or postmenopausal females became more similar to that observed in males, suggesting a convergence in risk profiles after the menopausal transition. In contrast, several traits, including short sleep duration, cancer,

hypertension, cataract, and macular degeneration, showed stronger associations with RAG in younger or premenopausal females. One possible explanation is that elevated RAG at younger ages may reflect early manifestation of conditions that typically emerge later in life, making them particularly informative markers of accelerated biological aging in this group. By comparison, in older or postmenopausal females, RAG may more strongly capture cumulative cardiometabolic and inflammatory processes that become more pronounced after the loss of estrogen's protective effects.

Revised manuscript text (Methods):
We then defined the retinal age gap (RAG) as the difference between the corrected predicted age and the participant's chronological age. To assess sex differences, we compared mean RAG values between females and males using independent-sample t-tests and quantified the effect size with Cohen's *d*. To explore potential hormonal influences, we also compared RAG across female subgroups defined by menopausal status (UKB only, not available for RS) and by age (<55 vs ≥55 years) using the same approach.
(...)
We conducted sex-stratified analyses to explore differential associations, and reported effect sizes as standardized beta coefficients or odds ratios. In females, we further repeated analyses stratified by menopausal status and by age group (<55 vs ≥55 years) to examine potential differences across life stages.
(...)
We conducted analyses in the full sample, stratified by sex, and additionally stratified females by menopausal status and age group (<55 vs ≥55 years) to examine potential sex- and life-stage–specific associations.

Added Supplementary Figures:

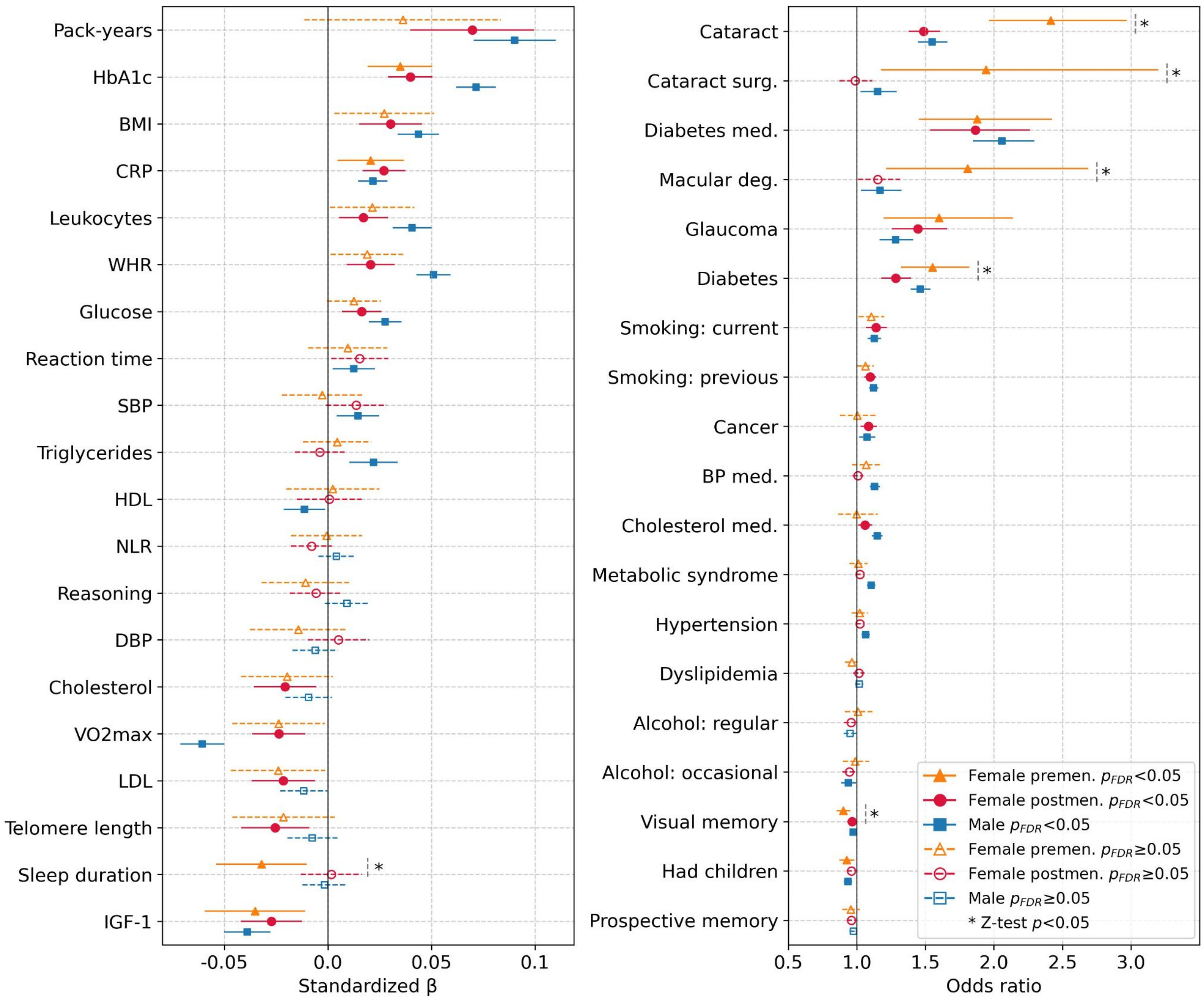

Figure S4. Models linking retinal age gap to risk factors and diseases in females stratified by menopause status and males (for visual comparison). (a) Ordinary least square linear regression and **(b)** logistic regression models were adjusted for socio-demographic factors. Outcomes are ordered by average effect size between the two female strata. Error bars represent the 95% confidence interval. Effects from premenopausal and postmenopausal females were compared using a *z*-test. Sample sizes and detailed results are shown in **Tables S2** and **S3**. BMI: body mass index, BP: blood pressure, CRP: C-reactive protein, DBP: diastolic blood pressure, deg.: degeneration, FDR: false discovery rate, HDL: high-density lipoprotein, IGF-1: insulin-like growth factor 1, LDL: low-density lipoprotein, med.: medication, NLR: neutrophil-to-lymphocyte ratio, SBP: systolic blood pressure, surg.: surgery, WHR: waist-to-hip ratio.

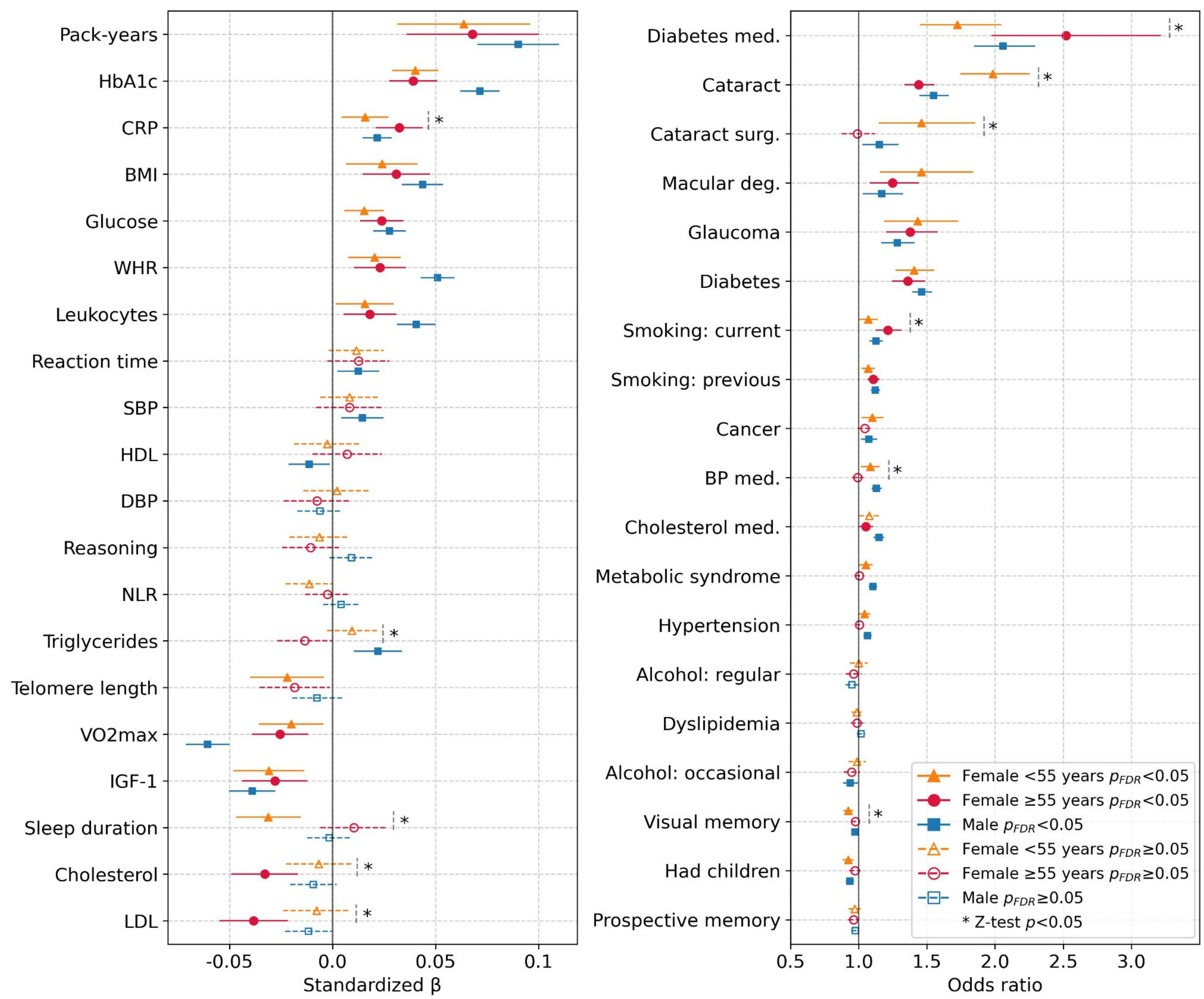

Figure S5. Models linking retinal age gap to risk factors and diseases in females stratified by age (<55 vs ≥55 years) and males (for visual comparison). (a) Ordinary least square linear regression and **(b)** logistic regression models were adjusted for socio-demographic factors. Outcomes are ordered by average effect size between the two female strata. Error bars represent the 95% confidence interval. Effects from younger and older females were compared using a *z*-test. Sample sizes and detailed results are shown in **Tables S2** and **S3**. BMI: body mass index, BP: blood pressure, CRP: C-reactive protein, DBP: diastolic blood pressure, deg.: degeneration, FDR: false discovery rate, HDL: high-density lipoprotein, IGF-1: insulin-like growth factor 1, LDL: low-density lipoprotein, med.: medication, NLR: neutrophil-to-lymphocyte ratio, SBP: systolic blood pressure, surg.: surgery, WHR: waist-to-hip ratio.

a

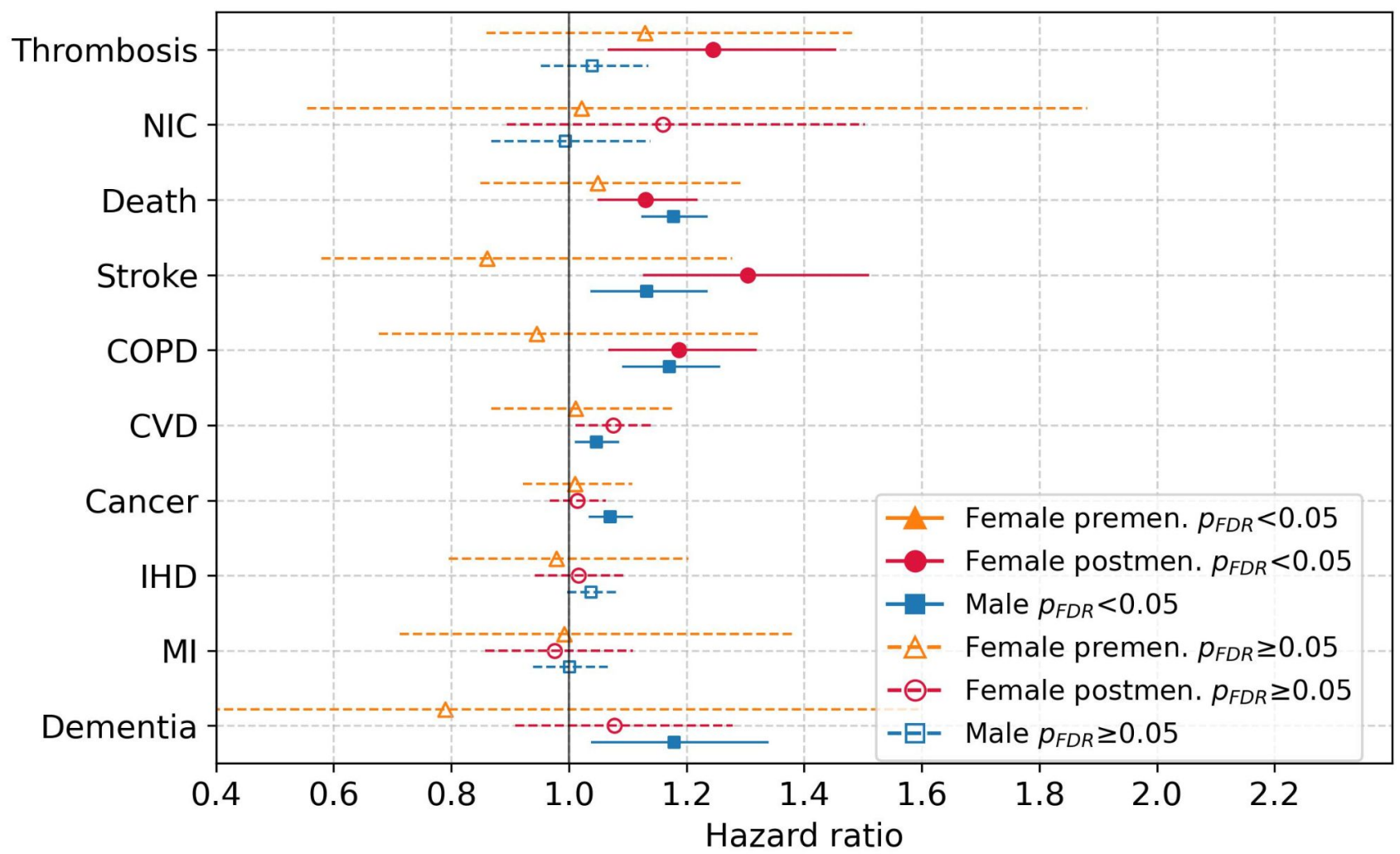

b

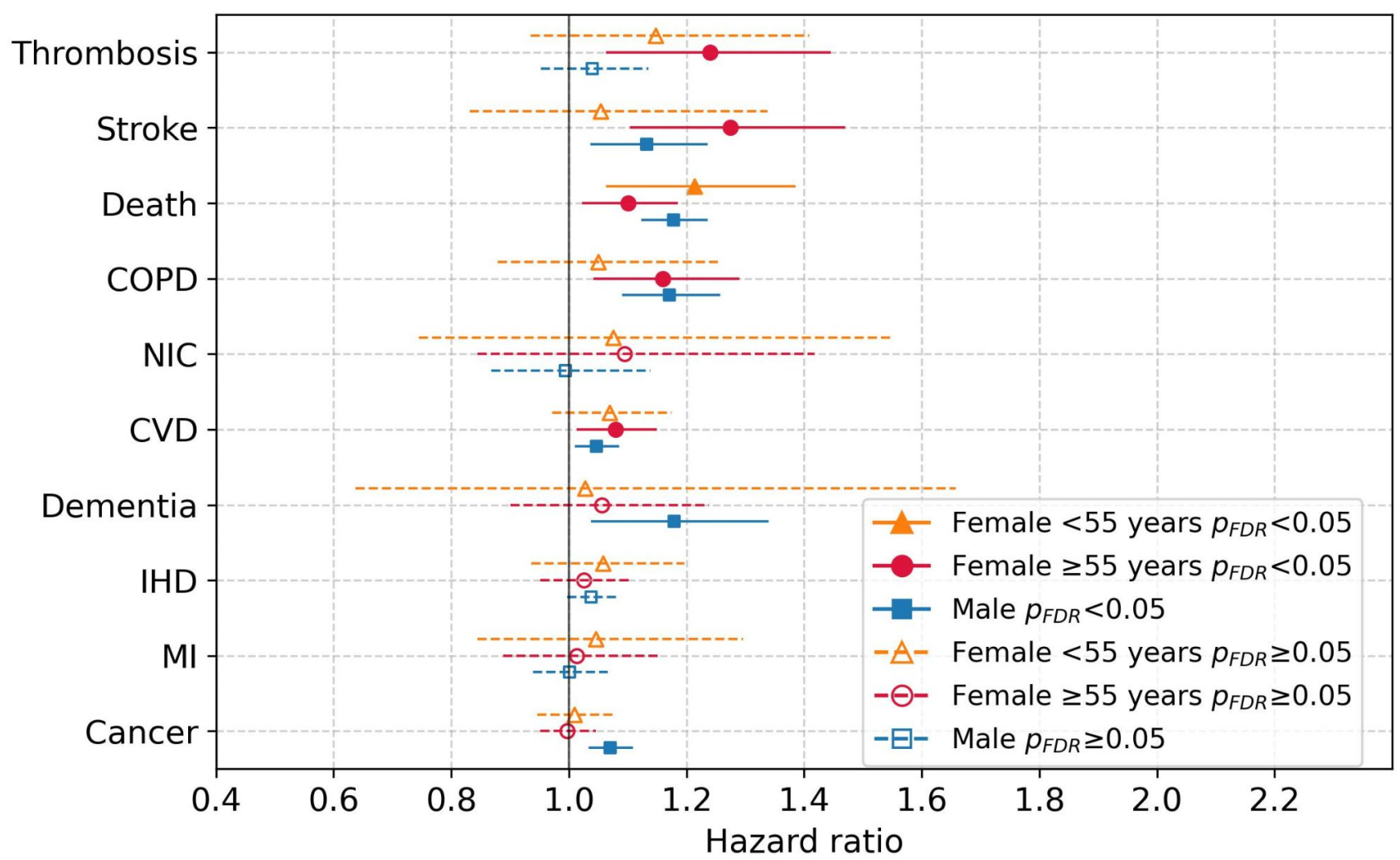

Figure S6. Retinal age gap associations with mortality and disease outcomes in female strata. Hazard ratios (HR) from Cox proportional hazards regression analyses linking retinal age gap to mortality and diseases are shown for females stratified by **(a)** menopausal status and **(b)** age (<55 vs ≥55 years), and males (for visual comparison). Models were adjusted for socio-demographic factors, common cardiovascular risk factors, and previous events from the same category. Outcomes are ordered by average HR between the two female strata. Error bars represent the 95% confidence interval. Effects from the female strata were compared using a *z*-test. Sample sizes, number of events, and detailed results are shown in **Table S4**. COPD: chronic obstructive pulmonary disease, CVD: cardiovascular disease, FDR: false discovery rate, IHD: ischemic heart disease, MI: myocardial infarction, NIC: non-ischemic cardiomyopathy.

Expanding the section biological interpretation of the sex-specific findings would strengthen the manuscript and provide a clearer context for the observed sex effects.

Response:
We are grateful to the Reviewer for addressing this important gap in our study. Based on the new results presented in the previous response, we expanded the biological interpretation of the sex-specific findings in the Discussion, including additional analyses stratified by menopausal status and age in females. We believe that these additions provide a clearer framework for understanding the observed sex differences and improve the overall interpretation of the results. We hope the Reviewer finds that these revisions strengthen the manuscript.

Revised manuscript text (Discussion):
We trained sex-specific models and found differences in RAG and its associations with clinical outcomes and genetic variants between females and males, with additional analyses suggesting that retinal aging patterns in females vary across the menopausal transition. (...)
Our stratified analyses suggest that hormonal and life-stage factors may also contribute. Specifically, RAG was lower in younger or premenopausal females but higher in older or postmenopausal females, placing males between these two groups. This pattern parallels epidemiological observations in which females experience lower cardiometabolic risk prior to menopause, followed by a marked increase afterward, including a rise in CVD incidence (Fasero & Coronado, 2025). Such transitions may influence retinal vascular and metabolic features that the model captures as aging-related signals.

Associations between RAG and cardiometabolic risk factors were stronger in males, consistent with the higher prevalence of metabolic syndrome and related conditions in men during midlife (Noubiap et al., 2025). However, when females were stratified by menopausal status or age, the pattern of associations in older or postmenopausal females became more similar to that observed in males, suggesting a convergence in risk profiles after the menopausal transition. In contrast, several traits, including short sleep duration, cancer, hypertension, cataract, and macular degeneration, showed stronger associations with RAG in younger or premenopausal females. One possible explanation is that elevated RAG at younger ages may reflect early manifestation of conditions that typically emerge later in life, making them particularly informative markers of accelerated biological aging in this group. By comparison, in older or postmenopausal females, RAG may more strongly capture cumulative cardiometabolic and inflammatory processes that become more pronounced after the loss of estrogen's protective effects.

References:

De Silva, S. R., Arno, G., Robson, A. G., Fakin, A., Pontikos, N., Mohamed, M. D., Bird, A. C., Moore, A. T., Michaelides, M., Webster, A. R., & Mahroo, O. A. (2021). The X-linked retinopathies: Physiological insights, pathogenic mechanisms, phenotypic features and

novel therapies. *Progress in Retinal and Eye Research*, *82*(100898), 100898. https://doi.org/10.1016/j.preteyeres.2020.100898

Fasero, M., & Coronado, P. J. (2025). Cardiovascular disease risk in women with menopause. *Journal of Clinical Medicine*, *14*(11), 3663. https://doi.org/10.3390/jcm14113663

Jackson, V. E., Wu, Y., Bonelli, R., Owen, J. P., Scott, L. W., Farashi, S., Kihara, Y., Gantner, M. L., Egan, C., Williams, K. M., Ansell, B. R. E., Tufail, A., Lee, A. Y., & Bahlo, M. (2025). Multi-omic spatial effects on high-resolution AI-derived retinal thickness. *Nature Communications*, *16*(1), 1317. https://doi.org/10.1038/s41467-024-55635-7

Noubiap, J. J., Nansseu, J. R., Nyaga, U. F., Ndoadoumgue, A. L., Ngouo, A. T., Tounouga, D. N., Tianyi, F.-L., Foka, A. J., Lontchi-Yimagou, E., Nkeck, J. R., & Bigna, J. J. (2025). Worldwide trends in metabolic syndrome from 2000 to 2023: a systematic review and modelling analysis. *Nature Communications*, *17*(1), 573. https://doi.org/10.1038/s41467-025-67268-5

Reviewer #2 (Remarks to the Author):

This manuscript presents a large-scale analysis of retinal age prediction using a fine-tuned foundation model (RETFound) on UK Biobank and Rotterdam Study datasets. The study is comprehensive, covering model benchmarking, sex-specific analyses, and downstream clinical associations. The scale of the dataset and the integration of multimodal downstream analyses make the work impactful.

> We appreciate the Reviewer's positive assessment of the scope and scale of our study, as well as their recognition of the value of integrating large imaging datasets with clinical and genetic analyses. The Reviewer's thoughtful comments helped us clarify several important methodological aspects and strengthen the presentation of the manuscript.

There are several major conceptual concerns regarding the definition, validity, and clinical usefulness of the proposed retinal age gap (RAG) that must be addressed. In particular, the manuscript should clarify (1) what biological construct RAG is actually measuring, (2) whether it is appropriate to train exclusively on nominally "normal" images, and (3) whether RAG adds predictive value beyond chronological age.

> **Response:**
> We thank the Reviewer for raising these important conceptual points regarding the interpretation and utility of the retinal age gap (RAG). In response, we have performed additional analyses and added clarifications to the manuscript.
>
> *(1) What biological construct RAG measures*

Conceptually, RAG measures individual deviation from the average aging trajectory of the reference population used to train the model (here the UK Biobank). Because the measure is derived from retinal images, it captures variation in biological processes that must be visibly reflected in those images, if not necessarily to the human eye, then to a computational model.

Consistent with this interpretation, our analyses show that RAG is associated with multiple domains of health, including ocular, cardiovascular, metabolic, inflammatory, and cognitive outcomes. Importantly, RAG is defined to be orthogonal to chronological age, meaning it isolates variation in retinal aging that is independent of age itself. In this sense, RAG is not intended to replace chronological age as a predictor, but rather to capture complementary information about inter-individual variation around the expected aging trajectory.

To clarify this conceptual definition, we have revised the Discussion.

Revised manuscript text (Discussion):
In this study, we fine-tuned RETFound to predict chronological age from retinal images, demonstrating robust generalization to an independent dataset with improved accuracy through averaging predictions from both eyes. The resulting retinal age gap (RAG)—the difference between predicted retinal age and chronological age—represents individual deviation from the average retinal aging trajectory of the reference population.
(...)
Overall, our study demonstrates that RAG offers a non-invasive, scalable marker of biological aging, sensitive to ocular, cardiovascular, metabolic, inflammatory, and cognitive dimensions of aging. Our findings highlight the potential of retinal imaging as an accessible tool for capturing systemic aging signals. Importantly, RAG is defined to be orthogonal to chronological age, thereby isolating independent variation in retinal aging. Our analyses further show that it contributes additional information beyond chronological age and conventional clinical risk factors.

(2) Training on nominally "normal" images

In our study, we applied a quality-control filter that excluded 25% of images. While this may appear substantial, many of these images were technically unusable (e.g., extremely dark or bright images, severe occlusion, or images where most of the retinal field was not visible). The remaining dataset still contained many images with visible pathologies, artifacts, and variable image quality; therefore, the retained images should not be interpreted as strictly "normal." Our intention was to remove only the most severe technical artifacts, while preserving clinically meaningful variation. We provide a more detailed explanation of this design choice in the response to the following Reviewer comment.

(3) Predictive value beyond chronological age

We agree that it is important to evaluate whether RAG adds information beyond chronological age and conventional risk factors. To address this point, we conducted additional analyses including likelihood ratio tests comparing nested models with and without RAG, comparisons

of model discrimination using the C-index, and leave-one-out analyses assessing the relative contribution of each covariate to model performance. These results are now reported in the manuscript and supplementary tables. Because these analyses are described in detail in response to the Reviewer's subsequent comment, we refer the Reviewer to that section for a full description.

Training exclusively on "normal" retinas may introduce bias and lead to unrealistic ground-truth assumptions. As outlined in the Methods, the model is trained to predict chronological age using UK Biobank fundus images that passed QC filtering. This process preferentially excludes diseased or poor-quality images. But individuals with early or subclinical systemic or ocular disease (e.g., early vascular dysfunction, preclinical AMD, diabetes) may still meet QC criteria. As a result, their retinal phenotype may already deviate from a healthy baseline. The model, therefore, implicitly treats chronological age as a clean ground truth, despite the likelihood that biological and pathological changes may precede clinical diagnosis. This mislabeling could bias the learned age signal.

Response:

The Reviewer raises an important point regarding the potential bias introduced by training the model on retinal images that passed quality control filtering. We would like to clarify that our study did not explicitly train the model on a "healthy" subset of individuals. Unlike some prior studies that exclude participants with diagnosed diseases during training, our approach aimed to train and evaluate the model on a general population sample.

The quality control procedure applied in this study was designed to remove poor-quality images, not individuals with disease. The filtering step primarily excluded images that were unusable for analysis (e.g., very dark, highly blurred, or otherwise severely degraded images). Importantly, participants with diagnosed systemic or ocular diseases, including diabetes and common eye diseases, remained represented in both the training and test datasets. The resulting dataset therefore still contains a substantial number of individuals with disease, as shown in Table S3 (see N cases).

We agree that image quality filtering can introduce a modest bias because certain diseases (e.g., cataract) may increase the likelihood of producing lower-quality images. This phenomenon has been previously quantified in our earlier work (Ortín Vela et al., 2024), where we demonstrated that increasingly stringent quality thresholds disproportionately exclude some individuals with disease (see the figure below). Based on that analysis, we selected a relatively lenient threshold that removes the lowest-quality 25% of images (red bars in the figure below), similar to the cutoff used in the reference study by Zekavat et al. (Zekavat et al., 2022). This represents a compromise between maintaining sufficient image quality for modeling and minimizing selection bias.

To clarify this point for readers, we have revised the Methods and Discussion sections of the manuscript to explicitly state that the QC procedure targeted image quality rather than disease status, and that individuals with diagnosed disease remained included in the analysis if their

images passed QC. The possible bias introduced by QC filtering is now acknowledged as a limitation in the Discussion.

[editorial note: third party material redacted]

Revised manuscript text (Methods):
To ensure image quality, we applied a quality control (QC) procedure based on the method described by Zekavat et al. (Zekavat et al., 2022), excluding the lowest-quality 25% of images. Briefly, a quality score was assigned to each image based on a deep learning model trained to identify poor-quality scans, and images below the 25th percentile of the score distribution were removed. Given the overall distribution of image quality in the UKB dataset, this threshold primarily excluded images that were severely degraded (e.g. very dark or highly blurred), while retaining images of moderate quality, including those from participants with ocular or systemic diseases.

Revised manuscript text (Discussion):
Furthermore, both cohorts represent relatively healthy, aging populations. The applied image quality filter may also introduce a modest bias toward excluding individuals with disease, as certain ocular conditions can increase the likelihood of producing lower-quality images (see (Ortín Vela et al., 2024) for a detailed quantification of this effect). Although individuals with diagnosed diseases remained represented in our study sample, the findings may not generalize to clinical populations with higher disease burden or atypical ocular presentations.

The paper needs to compare the proposed method with chronological age and standard risk factors, showing the clinical utility of RAG. The manuscript shows that RAG is statistically associated with mortality and multiple incident diseases (Fig. 3). However, it remains uncertain whether RAG provides incremental prognostic value beyond chronological age and conventional clinical factors. Several of the authors' own findings suggest that RAG may not offer additional predictive utility. First, Chronological age itself is a strong predictor of mortality. Second, many vascular features (e.g., vessel density, branching metrics) correlate with both RAG and chronological age in similar ways (Fig. 6), suggesting that RAG may largely reflect age-related vasculopathy rather than a distinct biological construct. Third, although the Cox models adjust for major risk factors, the manuscript does not present whether adding RAG improves model performance (e.g., change in C-index), how RAG compares directly with chronological age, or whether it enhances risk classification relative to established models. Without these key comparisons, it is difficult to assess the clinical relevance of RAG or determine whether it meaningfully improves risk prediction rather than recapitulating variance already explained by chronological age.

Response:
We agree that it is important to assess whether retinal age gap (RAG) provides prognostic information beyond chronological age and conventional clinical risk factors. To address this point, we performed several additional analyses quantifying the incremental predictive value of RAG.

First, we formally tested the contribution of RAG using likelihood ratio tests (LRT) comparing nested models with and without RAG. These analyses were conducted for all association models presented in Figures 2 and 3, including both risk factor associations and Cox proportional hazards models. In all cases where RAG was reported as significantly associated with outcomes in the original analysis, the LRT confirmed that inclusion of RAG significantly improved model fit. The corresponding χ^2 statistics and *p*-values have now been added to Tables S2-S4.

Second, we evaluated whether RAG improves predictive discrimination by comparing C-indices of models with and without RAG. We observed statistically significant improvements in discrimination for all-cause mortality in all samples, as well as for stroke and COPD in the combined and male samples. For other outcomes, the magnitude of these improvements was non-significant, even when RAG significantly improved model fit according to LRT, which is consistent with prior work showing that strong baseline predictors such as age and sex limit improvements in C-index for otherwise meaningful predictors (Zoccali & Tripepi, 2026).

Third, to further quantify the relative importance of RAG compared with other predictors (i.e., chronological age and standard risk factors), we performed a leave-one-out analysis of model contribution. Specifically, for each Cox model, we calculated the percent change in the model χ^2 statistic when each predictor was removed individually. This analysis estimates the loss of predictive information attributable to removing each variable. As expected, chronological age was the strongest contributor for most outcomes (with smoking ranking first for COPD). Importantly, RAG ranked among the top five contributors for five outcomes: mortality, COPD, stroke, dementia, and cancer (updated Figure 3). In contrast, RAG contributed less to prediction of CVD outcomes, where classical cardiovascular risk factors accounted for a larger share of the model's predictive power (Figures 3 and S7).

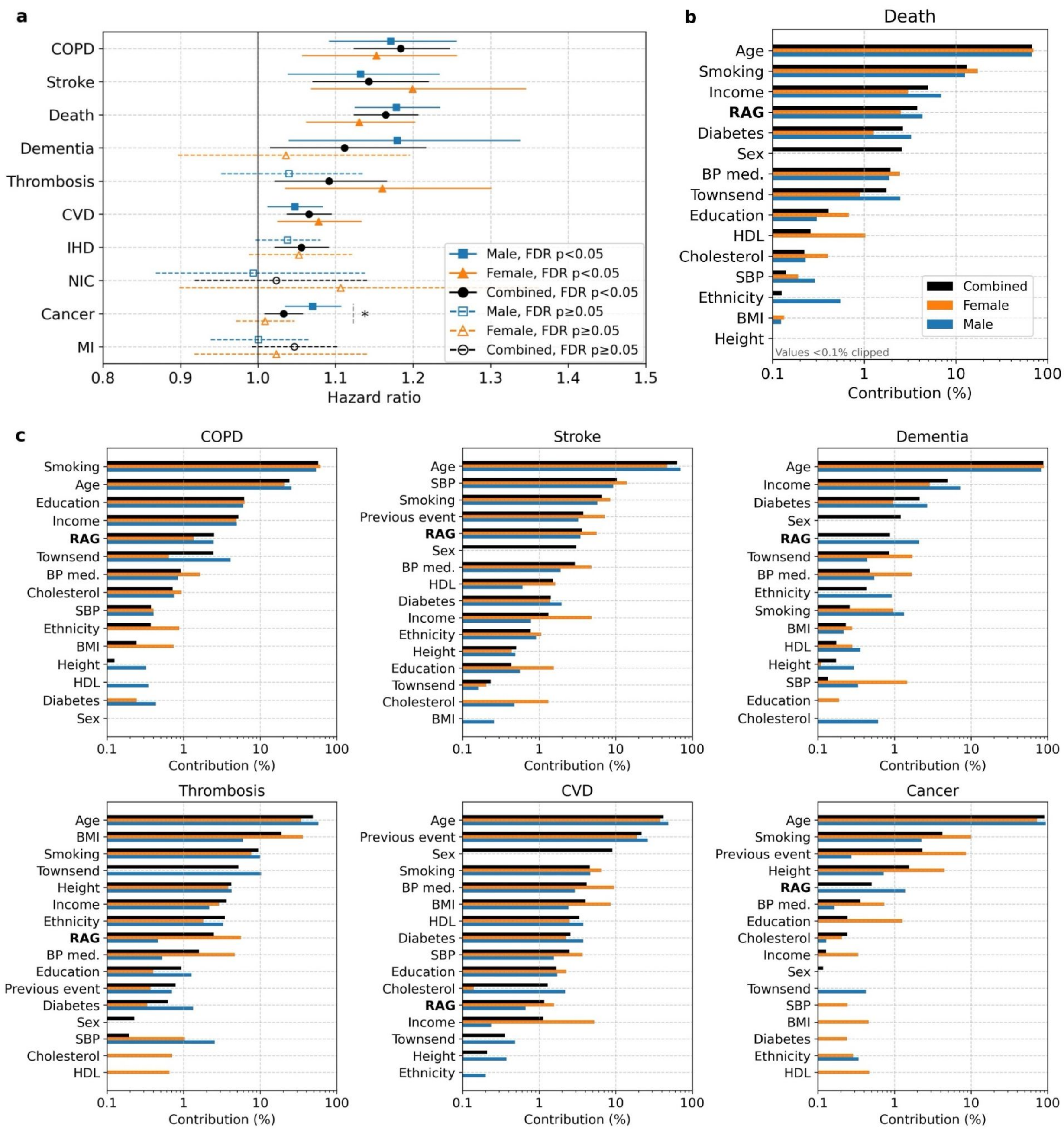

Figure 3. **Retinal age gap associations with mortality and disease outcomes.** **(a)** Hazard ratios (HR) from Cox proportional hazards regression analyses linking retinal age gap (RAG) to mortality and incident diseases in the combined, female, and male samples. Models were adjusted for socio-demographic factors, common cardiovascular risk factors, and previous events from the same category. Outcomes are ordered by average HR between the three sex categories. Error bars represent the 95% confidence interval. Female- and male-specific effects were compared using a *z*-test. Sample sizes, number of events, and detailed results are shown in **Table S4**. **(b)** Relative contribution of predictors to mortality risk in the multivariable Cox model, quantified as the percent change in model χ^2 when each variable is removed from the model. **(c)** Relative contribution of predictors to disease risk for selected outcomes using the same leave-one-out approach as in panel **(b)**. Additional cardiovascular outcomes (IHD, NIC, and MI) are shown in **Fig. S7**. Detailed results are presented in

Table S5. COPD: chronic obstructive pulmonary disease, CVD: cardiovascular disease, FDR: false discovery rate, IHD: ischemic heart disease, MI: myocardial infarction, NIC: non-ischemic cardiomyopathy.

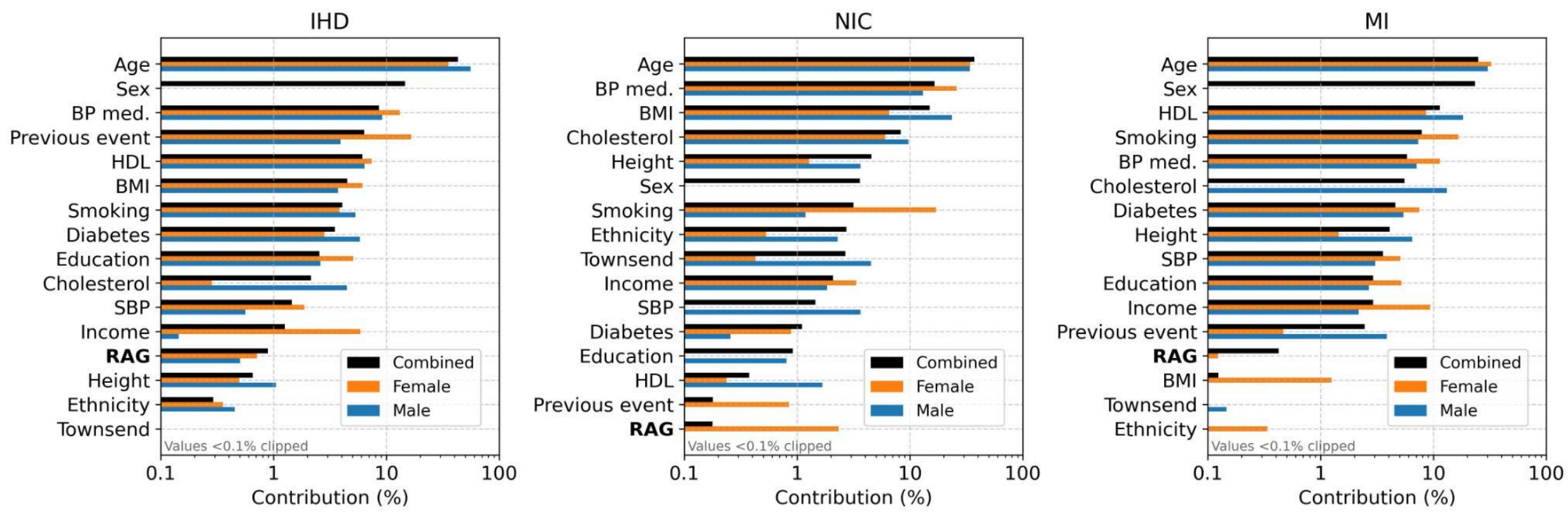

Figure S7. Relative contribution of predictors in Cox proportional hazards models for additional cardiovascular outcomes. Relative contribution of predictors to incident ischemic heart disease (IHD), myocardial infarction (MI), and non-ischemic cardiomyopathy (NIC) was quantified as the percent change in model χ^2 when each variable is removed from the multivariable Cox model. The analysis uses the same leave-one-out procedure as in **Fig. 3b–c**. Detailed results are presented in **Table S5**.

Regarding the Reviewer's comment on vascular image features (Figure 6), we would like to clarify that this analysis examined correlations between latent variables (LVs) from the retinal age estimation model and explicit retinal vascular features, not correlations with RAG itself. The observation that these LVs correlate with vascular metrics that also correlate with chronological age is expected and supports that the model captures biologically meaningful age-related retinal characteristics. To address the Reviewer's concern more directly, we have now computed correlations between these vascular features and RAG (added to Figure 6), which are substantially weaker than those observed with the model LVs or chronological age. Taken together, these findings suggest that:

1. The retinal age prediction model captures retinal features that track with chronological aging, as expected for a model trained to estimate age from retinal images.

2. RAG, the residual component orthogonal to age, shows substantially weaker correlations with explicit vascular features, indicating that it captures variation not explained by age or by commonly measured retinal vascular metrics.

Finally, regarding the suggestion that RAG may reflect age-related vasculopathy rather than a distinct biological construct, we note that this would not necessarily be undesirable. RAG is not intended to represent a completely separate biological process; rather, it is designed to integrate multiple aging-related signals present in retinal images into a single phenotype. If retinal vasculopathy represents an important manifestation of systemic aging, its contribution to RAG would be consistent with the intended interpretation of this measure.

Revised manuscript text (Methods):
To evaluate whether RAG contributed additional explanatory power beyond other covariates, we performed likelihood ratio tests comparing nested models with and without RAG.
(...)
To assess the incremental contribution of RAG, we performed likelihood ratio tests comparing nested Cox models with and without RAG. We corrected *p*-values for multiple testing (30 tests) using FDR (Benjamini & Hochberg, 1995) with an alpha threshold of 0.05. We also evaluated changes in model discrimination by comparing Harrell's C-index between the nested models using bootstrap resampling (200 iterations).

To quantify the relative importance of each predictor (including covariates), we conducted a leave-one-out analysis in which each variable was removed from the multivariable model one at a time and the resulting reduction in model fit was calculated from the change in log-likelihood. The corresponding chi-square statistic was computed for each variable and expressed as a percentage of the total chi-square across predictors, providing an estimate of each variable's relative contribution to the model.
(...)
Additionally, for the 24 image features, we computed correlations with the chronological age and RAG.

Revised manuscript text (Results):
Likelihood ratio tests comparing nested models with and without RAG confirmed that inclusion of RAG significantly improved model fit for all reported associations (**Table S2**).
(...)
Likelihood ratio tests confirmed that the inclusion of RAG significantly improved model fit for all reported associations (**Table S3**).
(...)
LRTs on nested models confirmed that inclusion of RAG significantly improved model fit for all reported survival associations (**Table S4**). Comparisons of model discrimination showed statistically significant improvements in C-index for all-cause mortality in all samples, as well as for stroke and COPD in the combined and male samples. For other outcomes, improvements in C-index were modest and not statistically significant despite significant improvements in model fit according to LRTs, consistent with findings that strong baseline predictors such as age and sex can limit changes in C-index for otherwise meaningful clinical predictors (Zoccali & Tripepi, 2026).

To further evaluate the importance of RAG relative to other predictors, we performed a leave-one-out analysis of the Cox models in which each covariate was removed individually and the resulting reduction in model fit was quantified. As expected, chronological age was the strongest contributor for most outcomes, while smoking was the dominant predictor for COPD. Notably, RAG ranked among the five largest contributors for five outcomes—mortality, COPD, stroke, dementia, and cancer (**Fig. 3b–c** and **Table S5**). In contrast, RAG contributed less to prediction of overall CVD, where classical cardiovascular risk factors accounted for a larger share of the model's predictive performance (**Fig. 3c**, **Fig. S7**, and **Table S5**).
(...)

By contrast, correlations between these features and RAG were weak (rightmost column of **Fig. 6**), indicating that the residual retinal age signal captured by RAG is largely independent of the explicit vascular and color features examined here.

Revised manuscript text (Discussion):
In fact, HRs were higher in females for stroke, thrombosis, and overall CVD, though not significantly so, likely due to limited statistical power (**Table S4**). Consistent with this pattern, the relative contribution of RAG to model prediction for these outcomes was also greater in females compared with males and the combined sample. While males have a higher overall risk of CVD [29], our findings suggest that this difference may be largely captured by traditional risk factors included in the models. In contrast, the additional variance captured by RAG may be more informative in females, potentially reflecting sex-specific biological aging processes not well represented by traditional risk metrics.
(...)
Overall, our study demonstrates that RAG offers a non-invasive, scalable marker of biological aging. Importantly, RAG is defined to be orthogonal to chronological age, thereby isolating independent variation in retinal aging. Our analyses further show that it contributes additional information beyond chronological age and conventional clinical risk factors.

Updated Figure 6:

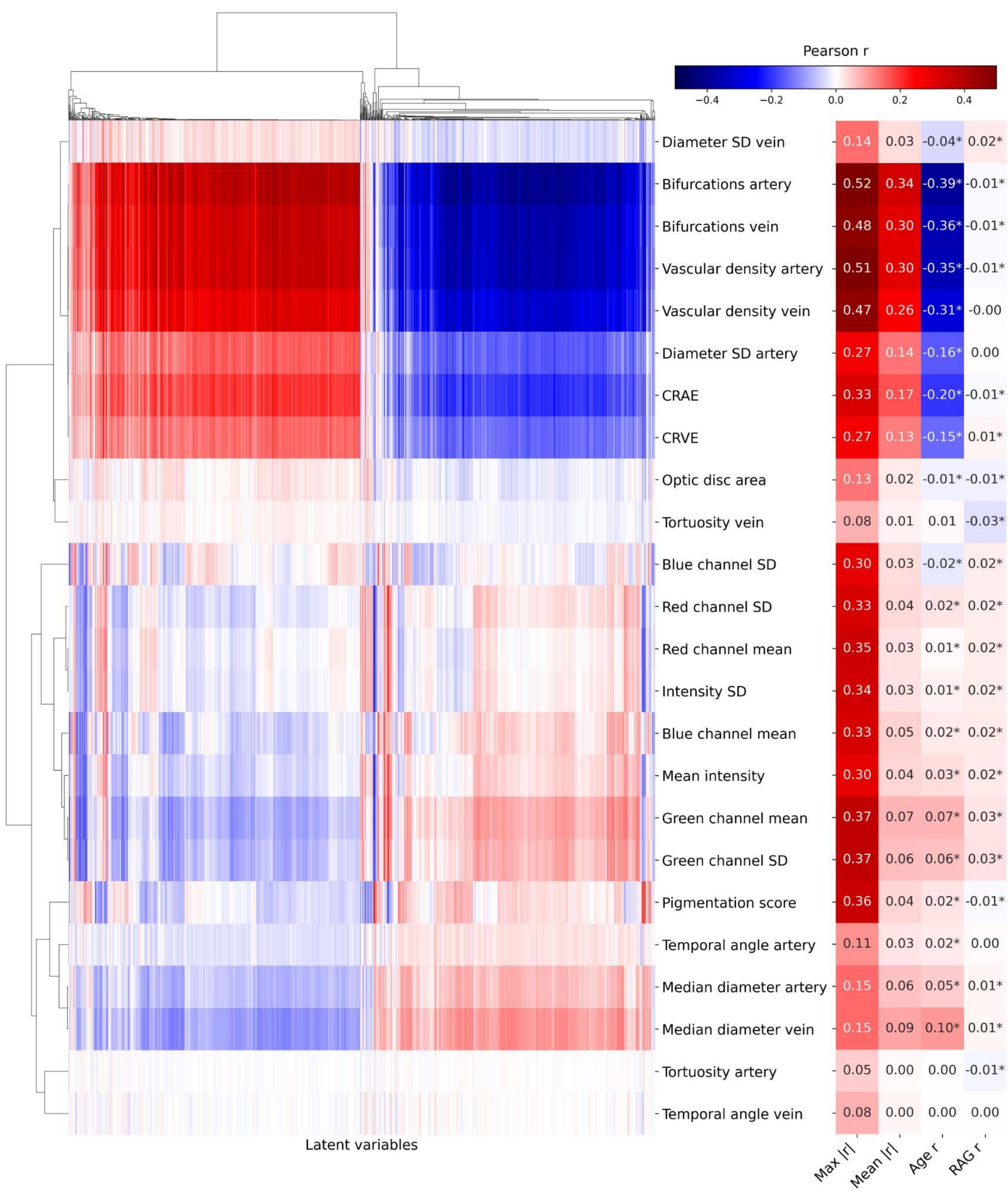

Figure 6. Correlation map between the model's 1024 latent variables and interpretable image features, in the combined sample for fold 1 (n=24,044 images). For every image feature, the maximum and mean absolute correlation with latent variables, the correlation with chronological age and retinal age gap are shown on the right side of the figure. CRAE: central retinal arterial equivalent, CRVE: central retinal venous equivalent, RAG: retinal age gap, SD: standard deviation, *: $p<0.05$.

Please clarify whether Cole's correction was applied uniformly across all downstream analyses (Fig. S1) and whether any sex-specific residual bias persists (e.g., Fig. S2b).

Response:
We thank the Reviewer for this clarification request.

(1) Application of Cole's correction

Yes, Cole's correction was applied prior to defining the RAG, and the corrected RAG was used consistently for all downstream analyses in the manuscript. To make this explicit, we have clarified this point in the **Methods** as follows:

We then defined the retinal age gap (RAG) as the difference between the corrected predicted age and the participant's chronological age. All subsequent analyses were performed using this corrected RAG measure.

(2) Sex-specific residual bias

We did observe a residual difference in RAG between females and males even after adjusting for height (Fig. S2b). We interpret this remaining difference as reflecting biological variation between sexes rather than a methodological artifact. To further investigate this pattern, we performed additional analyses stratified by age and menopausal status in females. These results provide further insight into the observed sex differences and are described in detail in our response to the next comment.

The manuscript reports that male retinas appear "younger," but the proposed explanations (e.g., differences in height or ocular dimensions) are not strongly supported. Additional empirical evidence or supplementary analyses would be needed to justify sex differences in retinal age prediction.

Response:
We are grateful to the Reviewer for raising this important point. We agree that the initially proposed explanations for the observed sex difference in RAG was partial and required further investigation. Given the cohort's age range and the strong hormonal and physiological changes occurring during menopause for females, we conducted additional analyses in females stratified by menopausal status and, separately, by age (<55 vs ≥55 years), to better understand sex-specific retinal aging patterns.

These analyses revealed that RAG differed across female life stages. Younger or premenopausal females had lower RAG on average, whereas older or postmenopausal females had higher RAG, with males positioned between these two groups. This pattern was also observed in the Rotterdam Study cohort, where older females exhibited higher RAG than younger females males, providing independent support for the life-stage–dependent pattern observed in UK Biobank.

In addition to differences in mean RAG, we observed distinct patterns of clinical associations across female life stages. Associations between RAG and cardiometabolic biomarkers were generally stronger in postmenopausal or older females and more closely resembled those observed in males. In contrast, several traits, including ocular diseases, sleep duration, cancer, and hypertension, showed stronger associations with RAG in younger or premenopausal females. Together, these findings suggest that the apparent sex differences in retinal age may partly reflect life-stage–dependent biological processes, with females showing relative protection before menopause followed by a convergence toward male-like cardiometabolic risk profiles afterwards.

We have incorporated the additional analyses into the Methods, Results, and Discussion sections of the revised manuscript and provided detailed results in the supplementary materials.

Revised manuscript text (Methods):
We then defined the retinal age gap (RAG) as the difference between the corrected predicted age and the participant's chronological age. To assess sex differences, we compared mean RAG values between females and males using independent-sample t-tests and quantified the effect size with Cohen's *d*. To explore potential hormonal influences, we also compared RAG across female subgroups defined by menopausal status (UKB only, not available for RS) and by age (<55 vs ≥55 years) using the same approach.
(...)
We conducted sex-stratified analyses to explore differential associations, and reported effect sizes as standardized beta coefficients or odds ratios. In females, we further repeated analyses stratified by menopausal status and by age group (<55 vs ≥55 years) to examine potential differences across life stages.
(...)
We conducted analyses in the full sample, stratified by sex, and additionally stratified females by menopausal status and age group (<55 vs ≥55 years) to examine potential sex- and life-stage–specific associations.

Revised manuscript text (Results):
Stratified analyses in females revealed that RAG was higher in postmenopausal than premenopausal females (Δ = 0.60, t = 10.7, p = 1e-26, d = 0.153), with male RAG positioned in between these groups (see **Fig. S3a**). Similar patterns were observed when stratifying females by age (<55 vs ≥55 years; **Fig. S3b**), with older females exhibiting higher RAG than younger females despite the orthogonalization of RAG to chronological age.

In the RS, we observed a similar pattern: males had lower mean RAG than females, indicating younger-appearing retinas (Δ = 0.37, t = 2.9, p = 0.004, Cohen's d = 0.085). When height was regressed out of the RAG, the sex difference was again reduced but not removed (Δ = 0.30, t = 2.4, p = 0.015, Cohen's d = 0.071). Cross-application of sex-specific models yielded consistent results, with male retinas evaluated using the female-trained model showing a mean RAG of -0.81, and female retinas evaluated using the male-trained model showing a mean RAG of 0.67. When stratifying females by age (<55 vs ≥55 years), RAG was higher in

older females than in younger females ($\Delta = 0.40$, $t = 2.29$, $p = 0.02$, $d = 0.09$) or males ($\Delta = 0.51$, $t = 3.59$, $p < 0.001$, $d = 0.12$), while there was no significant difference between younger females and males ($\Delta = 0.11$, $t = 0.63$, $p = 0.53$, $d = 0.02$). These findings replicate the UKB results and further support the observation that retinal age predictions tended to estimate postmenopausal/older female retinas as appearing older than retinas of males and premenopausal/younger females.
(...)
To further explore potential hormonal influences, we repeated association analyses in females stratified by menopausal status and, separately, by age (<55 vs ≥55 years) (**Fig. S4–S5** and **Tables S2–S3**). Overall, associations between higher RAG and adverse cardiometabolic and inflammatory biomarkers were stronger in postmenopausal/older females than in premenopausal/younger females, rendering the former more similar to males in effect size patterns. Notable exceptions included sleep duration, which was associated with lower RAG only in premenopausal/younger females, and cataract, macular degeneration, cancer, and hypertension, for which associations were stronger in premenopausal/younger females. Interestingly, triglycerides, LDL, and total cholesterol were associated with lower RAG specifically in postmenopausal/older females, a pattern not observed in premenopausal/younger females or in males.
(...)
When stratifying females by menopausal status or age group (**Fig. S6**), most incident disease associations were driven by postmenopausal/older females, whereas associations in premenopausal/younger females were generally weaker and often not statistically significant, possibly reflecting both biological differences and lower event counts in the younger stratum. An exception was all-cause mortality, which showed significant effect in both younger and older females.

Revised manuscript text (Discussion):
We trained sex-specific models and found differences in RAG and its associations with clinical outcomes and genetic variants between females and males, with additional analyses suggesting that retinal aging patterns in females vary across the menopausal transition.
(...)
Our stratified analyses suggest that hormonal and life-stage factors may also contribute. Specifically, RAG was lower in younger or premenopausal females but higher in older or postmenopausal females, placing males between these two groups. This pattern parallels epidemiological observations in which females experience lower cardiometabolic risk prior to menopause, followed by a marked increase afterward, including a rise in CVD incidence (Fasero & Coronado, 2025). Such transitions may influence retinal vascular and metabolic features that the model captures as aging-related signals.

Associations between RAG and cardiometabolic risk factors were stronger in males, consistent with the higher prevalence of metabolic syndrome and related conditions in men during midlife (Noubiap et al., 2025). However, when females were stratified by menopausal status or age, the pattern of associations in older or postmenopausal females became more similar to that observed in males, suggesting a convergence in risk profiles after the menopausal transition. In contrast, several traits, including short sleep duration, cancer,

hypertension, cataract, and macular degeneration, showed stronger associations with RAG in younger or premenopausal females. One possible explanation is that elevated RAG at younger ages may reflect early manifestation of conditions that typically emerge later in life, making them particularly informative markers of accelerated biological aging in this group. By comparison, in older or postmenopausal females, RAG may more strongly capture cumulative cardiometabolic and inflammatory processes that become more pronounced after the loss of estrogen's protective effects.

Supplementary Figure (clarifying sex difference in RAG):

a

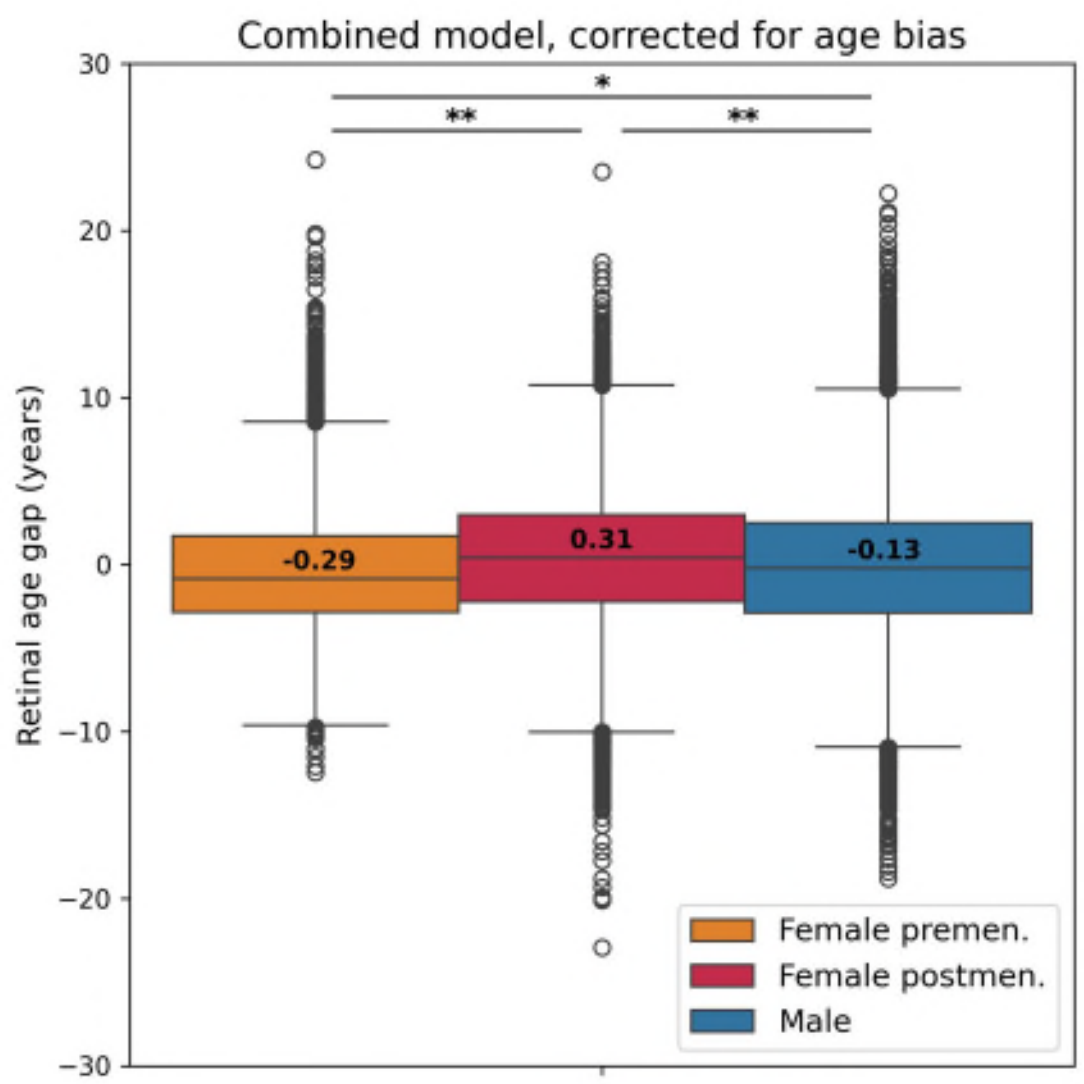

b

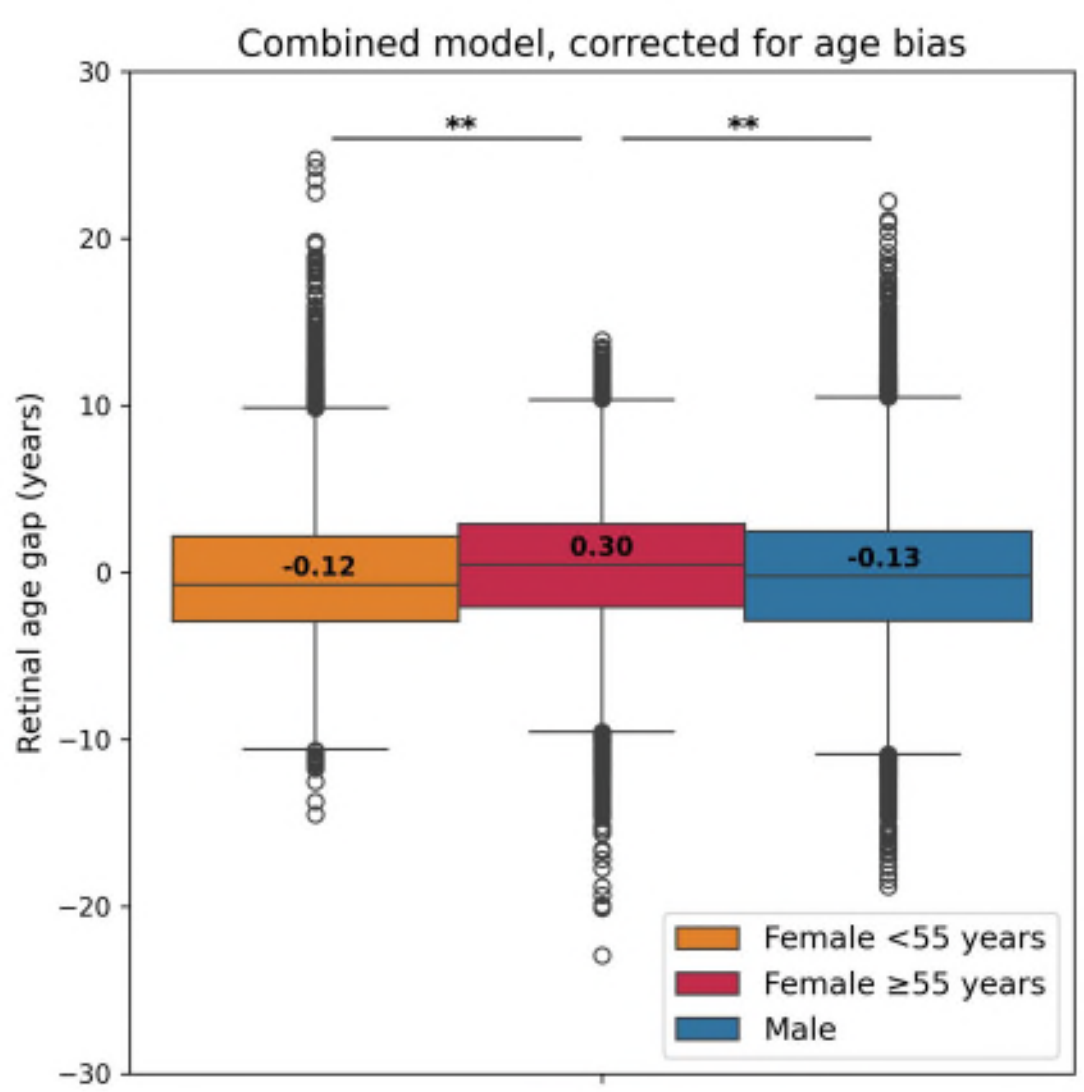

Figure S3. Comparison of retinal age gap between female strata and males. Females were stratified by **(a)** menopausal status (pre vs post) and **(b)** age (<55 vs ≥55 years). Retinal age gap was estimated from the combined-sample model, after correction for the regression-to-the-mean bias. *: $p<0.05$, **: $p<0.001$ from t-test comparing groups.

Although saliency and attention maps highlight the macula, optic disc, and vessels, such methods do not necessarily reveal the underlying biological mechanisms.

Response:
We agree with the Reviewer that saliency and attention maps alone cannot establish the biological mechanisms underlying model predictions. Their role in this study is therefore to provide interpretative context for the model's predictions rather than direct evidence of underlying biological mechanisms. In the revised manuscript, we emphasize that mechanistic interpretations rely not on these visualizations in isolation but on the *convergence of evidence* from multiple complementary analyses.

For example, the observation that the model's attention highlights major retinal vessels, particularly in females, is consistent with our genetic findings, which identified associations with genes involved in retinal vasculature in females but not males. The convergence of attention maps and genetic results therefore supports the hypothesis that aspects of vascular remodeling may contribute to retinal aging in females more than in males.

Similarly, the model's attention to the optic disc, combined with the association between RAG and cognitive performance, future dementia risk, and genes linked to Alzheimer's disease, all point to the possibility that structural variation in the optic nerve may reflect broader central nervous system aging processes. While these interpretations remain speculative given the observational and cross-sectional nature of the study, the convergence of genetic, clinical, and imaging evidence increases the plausibility of these hypotheses.

We have added this consideration to the limitations section of the Discussion, clarifying that our interpretations rely on converging evidence from multiple complementary analyses.

Revised manuscript text (Discussion):
Finally, although our interpretations are supported by converging evidence from genetic analyses, saliency and attention maps, correlations with interpretable image features, and clinical associations, the precise biological mechanisms underlying the age-related signals captured by the models remain incompletely understood and will require further investigation in longitudinal and mechanistic studies.

The authors should discuss how differences in phenotype definitions, imaging protocols, population characteristics, and genetic replication variability across datasets may contribute to discrepancies in results. Additionally, please clarify whether these cohort-level differences could affect the stability or generalizability of the retinal age model, and how future work should account for such dataset-related inconsistencies.

Response:
We thank the Reviewer for highlighting the importance of factors that may generate discrepancies across retinal age studies and affect model generalizability. To address this point, we have clarified in the manuscript which aspects were harmonized between the UK

Biobank (UKB) discovery analysis and the Rotterdam Study (RS) replication analysis, and which factors remain potential sources of heterogeneity.

First, regarding *phenotype definition*, differences across studies may arise from both the modeling framework and the definition of the age-gap phenotype. Model architecture and training strategy can influence performance and learned representations; for example, convolutional neural networks (CNNs) have traditionally been used in retinal age prediction, whereas our study fine-tunes RETFound, a Vision Transformer–based foundation model pretrained on retinal images. We discuss these architectural considerations and their impact on performance in the **Introduction** and **Discussion:**

> Traditionally, deep learning in biomedical imaging has relied on convolutional neural networks (CNNs), but recent advances have introduced Vision Transformers as viable alternatives. While there is no consensus on which architecture performs best overall, evidence suggests performance is task- and data-dependent. For instance, Vision Transformers often outperform CNNs in classification tasks when large datasets and pretraining are available, whereas CNN-based models like nnU-Net continue to excel in 3D medical image segmentation, particularly when rigorously validated and carefully configured (Isensee et al., 2024; Takahashi et al., 2024). RETFound, a foundation model for retinal images pretrained using a masked autoencoder with a large Vision Transformer backbone, has shown strong performance when fine-tuned for disease classification tasks, outperforming other contrastive learning-based approaches (Zhou et al., 2023).
>
> (...)
>
> With an MAE of 2.85 years and an R^2 of 0.80, RETFound fine-tuned to predict age on UKB data outperformed previous CNN-based models, which have reported MAE between 3.08 and 4.5 years and R^2 between 0.51 and 0.77 (Ahadi et al., 2023; Le Goallec et al., 2021; Poplin et al., 2018; Rim et al., 2020; Zhu et al., 2023). Although foundation models require considerable computational resources to pretrain, our results suggest they offer a measurable benefit in downstream regression tasks such as age prediction.

We clarified the use of regression-to-the-mean correction following Cole et al. to obtain an age gap measure that is independent of chronological age. This correction is commonly applied in brain age studies but less consistently in retinal age studies. We now describe this rationale more explicitly in the **Methods (revised manuscript):**

> Age-prediction models commonly exhibit regression-to-the-mean bias, whereby younger individuals tend to have their age overestimated and older individuals underestimated. This phenomenon induces a dependence between the resulting age gap and chronological age, which can introduce confounding in downstream analyses. To mitigate this bias, post hoc corrections are frequently applied in brain age studies but have been used less consistently in retinal age studies (de Lange & Cole, 2020). To correct for regression-to-the-mean bias in predicted age, we applied a post hoc adjustment following the procedure described by Cole et al. (Cole et al., 2018). Specifically, we fit a linear

regression of predicted age on chronological age to estimate the slope (α) and intercept (β), then adjusted predicted age by subtracting β and dividing by α.

Second, we acknowledge that differences in *imaging protocols*, notably the camera model, field-of-view, and image quality control, can affect model performance and downstream analyses. In the replication analysis, we therefore selected color fundus images acquired with imaging settings most comparable to those used in UKB and applied similar quality-control procedures and age-range filters. We have added clarifications to the **Methods** explaining this harmonization strategy:

> Because discrepancies in results may arise from differences in imaging protocols and participant demographic characteristics, we aimed to select the subset of RS images most comparable to those used in the UKB analysis. For the model replication, CFIs acquired with the Topcon 3D OCT-2000 FA plus camera with a macula-centered field-of-view were used, as this modality was deemed most comparable to the UKB imaging while offering broad availability within the RS. In total, 37,771 fundus images were available from 7480 participants (14,912 eyes across 8813 visits). After filtering by age (40 < age ≤ 70 years, a range encompassing 97% of the UKB sample) and image quality (excluding the lowest 25%), 10,354 images from 4757 participants were retained for analysis (mean age = 58.0 ± 7.2 years).

Third, *population characteristics* can influence model behavior and generalizability. Both UKB and RS are predominantly composed of relatively healthy individuals of White European ancestry and share a similar age range, which reduces heterogeneity within our analyses but may limit generalizability to other populations. These points are discussed as study limitations **(revised Discussion):**

> Although RETFound was pre-trained on data with some ethnic diversity, both the UKB and RS cohorts analyzed here were predominantly composed of individuals of White European ancestry. As such, the generalizability of our results to other populations remains to be evaluated. Broader representation in training datasets will be essential to ensuring equitable performance across ancestries. Furthermore, both cohorts represent relatively healthy, aging populations. The applied image quality filter may also introduce a modest bias toward excluding individuals with disease, as certain ocular conditions can increase the likelihood of producing lower-quality images (see (Ortín Vela et al., 2024) for a detailed quantification of this effect). Although individuals with diagnosed diseases remained represented in our study sample, the findings may not generalize to clinical populations with higher disease burden or atypical ocular presentations.

Finally, for *genetic analyses*, we note that the RS replication sample is smaller than UKB, which may limit statistical power. For this reason, replication focused on testing signals identified in the discovery analysis rather than performing a fully independent genome-wide discovery **(Methods):**

> To assess replication of genetic findings, we adopted a candidate approach, applying FDR correction with a threshold 0.05 to *p*-values from SNPs, respectively genes, that were genome-wide significant in the UKB.

In addition, due to cohort-specific data structures, different imputation reference panels and GWAS software were used (UK10K and Regenie in UKB; Haplotype Reference Consortium v1.1 and PLINK 2.0 in RS), and GWAS results in RS were combined across sub-cohorts through meta-analysis. While such methodological differences could in principle influence individual variant-level results, many associations were successfully replicated, particularly at the gene level, suggesting that these differences did not excessively affect results. We clarify these aspects in the **Methods**:

> For the replication analysis, because imputation and QC were conducted independently in the four RS sub-cohorts, we first ran GWAS within each cohort and subsequently combined the results in a meta-analysis. Genotypes were imputed using the Haplotype Reference Consortium reference panel version 1.1 [69]. For the individual GWAS, we applied linear regression models in PLINK 2.0 [70], adjusting for age, sex, age-squared, age-by-sex, age-squared-by-sex, height, height-squared, spherical power, spherical power-squared, cylindrical power, cylindrical power-squared, and the first 10 genetic PCs. We then meta-analyzed results across cohorts using an inverse-variance weighted fixed-effects approach implemented in METAL [71].

Overall, while some dataset differences cannot be fully controlled (e.g., imaging hardware or cohort composition), we minimized methodological discrepancies between discovery and replication analyses by harmonizing phenotype definitions, age ranges, imaging modalities, and analytical procedures wherever possible.

References:

Ahadi, S., Wilson, K. A., Babenko, B., McLean, C. Y., Bryant, D., Pritchard, O., Kumar, A., Carrera, E. M., Lamy, R., Stewart, J. M., Varadarajan, A., Berndl, M., Kapahi, P., & Bashir, A. (2023). Longitudinal fundus imaging and its genome-wide association analysis provide evidence for a human retinal aging clock. *eLife*, *12*. https://doi.org/10.7554/eLife.82364

Benjamini, Y., & Hochberg, Y. (1995). Controlling the False Discovery Rate: A Practical and Powerful Approach to Multiple Testing. *Journal of the Royal Statistical Society. Series B, Statistical Methodology*, *57*(1), 289–300. https://doi.org/10.1111/j.2517-6161.1995.tb02031.x

Cole, J. H., Ritchie, S. J., Bastin, M. E., Valdés Hernández, M. C., Muñoz Maniega, S., Royle, N., Corley, J., Pattie, A., Harris, S. E., Zhang, Q., Wray, N. R., Redmond, P., Marioni, R. E., Starr, J. M., Cox, S. R., Wardlaw, J. M., Sharp, D. J., & Deary, I. J. (2018). Brain age predicts mortality. *Molecular Psychiatry*, *23*(5), 1385–1392. https://doi.org/10.1038/mp.2017.62

de Lange, A.-M. G., & Cole, J. H. (2020). Commentary: Correction procedures in brain-age prediction. *NeuroImage. Clinical*, *26*(102229), 102229. https://doi.org/10.1016/j.nicl.2020.102229

Fasero, M., & Coronado, P. J. (2025). Cardiovascular disease risk in women with menopause. *Journal of Clinical Medicine*, *14*(11), 3663. https://doi.org/10.3390/jcm14113663

Isensee, F., Wald, T., Ulrich, C., Baumgartner, M., Roy, S., Maier-Hein, K., & Jäger, P. F. (2024). NnU-net revisited: A call for rigorous validation in 3D medical image segmentation. In *Lecture Notes in Computer Science* (pp. 488–498). Springer Nature Switzerland. https://doi.org/10.1007/978-3-031-72114-4_47

Le Goallec, A., Diai, S., Collin, S., Vincent, T., & Patel, C. J. (2021). Deep learning of fundus and optical coherence tomography images enables identification of diverse genetic and environmental factors associated with eye aging. In *medRxiv*. medRxiv. https://doi.org/10.1101/2021.06.24.21259471

Noubiap, J. J., Nansseu, J. R., Nyaga, U. F., Ndoadoumgue, A. L., Ngouo, A. T., Tounouga, D. N., Tianyi, F.-L., Foka, A. J., Lontchi-Yimagou, E., Nkeck, J. R., & Bigna, J. J. (2025). Worldwide trends in metabolic syndrome from 2000 to 2023: a systematic review and modelling analysis. *Nature Communications*, *17*(1), 573. https://doi.org/10.1038/s41467-025-67268-5

Ortín Vela, S., Beyeler, M. J., Trofimova, O., Iuliani, I., Vargas Quiros, J. D., de Vries, V. A., Meloni, I., Elwakil, A., Hoogewoud, F., Liefers, B., Presby, D., Ramdas, W. D., Tomasoni, M., Schlingemann, R., Klaver, C. C. W., & Bergmann, S. (2024). Phenotypic and genetic characteristics of retinal vascular parameters and their association with diseases. *Nature Communications*, *15*(1), 9593. https://doi.org/10.1038/s41467-024-52334-1

Poplin, R., Varadarajan, A. V., Blumer, K., Liu, Y., McConnell, M. V., Corrado, G. S., Peng, L., & Webster, D. R. (2018). Prediction of cardiovascular risk factors from retinal fundus photographs via deep learning. *Nature Biomedical Engineering*, *2*(3), 158–164. https://doi.org/10.1038/s41551-018-0195-0

Rim, T. H., Lee, G., Kim, Y., Tham, Y.-C., Lee, C. J., Baik, S. J., Kim, Y. A., Yu, M., Deshmukh, M., Lee, B. K., Park, S., Kim, H. C., Sabayanagam, C., Ting, D. S. W., Wang, Y. X., Jonas, J. B., Kim, S. S., Wong, T. Y., & Cheng, C.-Y. (2020). Prediction of systemic biomarkers from retinal photographs: development and validation of deep-learning algorithms. *The Lancet. Digital Health*, *2*(10), e526–e536. https://doi.org/10.1016/S2589-7500(20)30216-8

Takahashi, S., Sakaguchi, Y., Kouno, N., Takasawa, K., Ishizu, K., Akagi, Y., Aoyama, R., Teraya, N., Bolatkan, A., Shinkai, N., Machino, H., Kobayashi, K., Asada, K., Komatsu, M., Kaneko, S., Sugiyama, M., & Hamamoto, R. (2024). Comparison of vision transformers and convolutional neural networks in medical image analysis: A systematic review. *Journal of Medical Systems*, *48*(1), 84. https://doi.org/10.1007/s10916-024-02105-8

Zekavat, S. M., Raghu, V. K., Trinder, M., Ye, Y., Koyama, S., Honigberg, M. C., Yu, Z.,

Pampana, A., Urbut, S., Haidermota, S., O'Regan, D. P., Zhao, H., Ellinor, P. T., Segrè, A. V., Elze, T., Wiggs, J. L., Martone, J., Adelman, R. A., Zebardast, N., … Natarajan, P. (2022). Deep learning of the retina enables phenome- and genome-wide analyses of the microvasculature. *Circulation*, *145*(2), 134–150. https://doi.org/10.1161/CIRCULATIONAHA.121.057709

Zhou, Y., Chia, M. A., Wagner, S. K., Ayhan, M. S., Williamson, D. J., Struyven, R. R., Liu, T., Xu, M., Lozano, M. G., Woodward-Court, P., Kihara, Y., UK Biobank Eye & Vision Consortium, Altmann, A., Lee, A. Y., Topol, E. J., Denniston, A. K., Alexander, D. C., & Keane, P. A. (2023). A foundation model for generalizable disease detection from retinal images. *Nature*, *622*(7981), 156–163. https://doi.org/10.1038/s41586-023-06555-x

Zhu, Z., Shi, D., Guankai, P., Tan, Z., Shang, X., Hu, W., Liao, H., Zhang, X., Huang, Y., Yu, H., Meng, W., Wang, W., Ge, Z., Yang, X., & He, M. (2023). Retinal age gap as a predictive biomarker for mortality risk. *The British Journal of Ophthalmology*, *107*(4), 547–554. https://doi.org/10.1136/bjophthalmol-2021-319807

Zoccali, C., & Tripepi, G. (2026). The conservativeness of standard C statistics in the prediction of clinical events. *European Journal of Clinical Investigation*, *56*(1), e70150. https://doi.org/10.1111/eci.70150

We would like to sincerely thank all three Reviewers for their time, expertise, and thoughtful evaluation of our manuscript. We are particularly grateful for the opportunity to further revise our work and address the important concerns raised by Reviewer #2, together with the thorough and constructive feedback provided by Reviewer #3. We believe that the revisions have substantially strengthened the manuscript and improved the rigor and clarity of our interpretations.

The principal revisions are summarized as follows:

1. We refined the interpretation of RAG as a retinal imaging-derived aging-associated phenotype and clarified the distinction between the age-prediction model and the residual RAG phenotype.
2. We further qualified the biological interpretation of RAG, including potential contributions from non-aging-related image characteristics and a more cautious interpretation of genetic associations.
3. We revised the Abstract and Discussion to avoid overstating the immediate clinical utility of RAG and to better align conclusions with the observed effect sizes.
4. We refined the interpretation of sex-specific findings by emphasizing the life-stage-dependent patterns revealed by menopausal stratification.
5. We improved methodological transparency by clarifying RAG derivation, the use of combined versus sex-specific models, and covariate adjustment strategies across analyses.

A detailed point-by-point response to each comment is provided below. We hope that these revisions adequately address the remaining concerns.

Reviewer #1 (Remarks to the Author):

The authors have answered my questions.

Response:
We thank the Reviewer for the thoughtful comments and constructive feedback provided during the review process. We believe these suggestions helped improve the clarity and overall quality of the manuscript.

Reviewer #2 (Remarks to the Author):

Thanks for having a chance to go through the revised version and the rebuttal. Overall, I still have reservations regarding approaches that train age-prediction models on broadly “normal” images and subsequently interpret the residual (RAG) as a biologically meaningful signal. In line with Reviewer #2, I outline several concerns below.

Response:

We thank the Reviewer for carefully evaluating the revised manuscript and for raising these conceptual concerns regarding "age-gap" approaches. We agree that the interpretation of residual-based biomarkers warrants careful consideration and nuanced discussion.

First, we would like to better contextualize our work within a rapidly growing body of literature that uses analogous frameworks across multiple biological domains. In particular, "brain age gap" approaches have been extensively studied and associated with a wide range of neurological and systemic outcomes (see systematic reviews in [1–3]). Similar paradigms have also been applied to retinal imaging [4], other organ systems [5,6], and molecular or proteomic aging measures [7–9]. More recently, a *Nature* study examining sleep patterns in relation to multi-organ biological age gaps adopted a highly comparable methodological framework [10]. These examples illustrate that residual-based aging phenotypes are increasingly being investigated as potentially informative biomarkers of health and disease risk.

Importantly, however, we fully agree with the Reviewer that widespread adoption does not in itself establish biological validity. We are therefore grateful for these comments, which prompted us to refine how we describe and interpret the retinal age gap (RAG) phenotype throughout the manuscript. In the revised version, we have taken greater care to avoid overinterpreting RAG as a direct measure of a single biological process, and instead frame it more appropriately as an imaging-derived phenotype associated with aging-related health outcomes.

We hope these revisions clarify the conceptual positioning of our work and its relevance within the broader biological age literature.

1. Unclear biological interpretation of RAG

RAG is defined as the residual from chronological age, which is a mathematical construct rather than a biologically validated measure. Although RAG demonstrates associations with multiple outcomes, these relationships do not elucidate the underlying biological processes it reflects (e.g., vascular aging versus heterogeneous or nonspecific effects). Moreover, its weak correlation with interpretable retinal features raises the possibility that RAG primarily captures residual variation rather than a well-defined biological signal.

Response:

We agree with the Reviewer that RAG is, by definition, a residual-based construct and should not be interpreted as a direct or fully characterized measure of a specific biological aging process. We have therefore revised the manuscript to more carefully frame RAG as an imaging-derived aging-associated phenotype rather than a definitive mechanistic biomarker.

(Discussion):

"Overall, our study demonstrates that RAG represents a non-invasive retinal imaging-derived aging-associated phenotype linked to ocular, cardiovascular, metabolic, inflammatory, and cognitive dimensions of aging.

At the same time, we do not think that RAG should therefore be considered merely an arbitrary mathematical residual without biological relevance. Residual-based "age-gap" phenotypes across multiple modalities have repeatedly demonstrated robust cross-sectional and prognostic associations with disease, mortality, and other aging-related outcomes in independent cohorts [1–10]. Many investigators would consider such reproducible associations, particularly when they persist after adjustment for conventional risk factors, as evidence that these phenotypes capture biologically meaningful variation related to health and aging.

Importantly, our study goes beyond reporting outcome associations alone. As noted by the Reviewer, understanding the biological basis of these signals remains an important challenge. Although necessarily incomplete, our work attempts to address this question through a combination of analyses that, to our knowledge, has not previously been applied together in retinal age-gap studies. Specifically, we performed extensive phenotypic association analyses across metabolic, inflammatory, cardiovascular, cognitive, lifestyle, and ocular variables, including sex-stratified analyses, as well as a genome-wide association study of RAG. Together, these analyses provide important insights into the potential biological processes associated with the phenotype. Nevertheless, as acknowledged in the Discussion, *"the precise biological mechanisms underlying the age-related signals captured by the models remain incompletely understood and will require further investigation in longitudinal and mechanistic studies."*

Regarding the relatively weak correlations with predefined retinal features, we believe this finding does not necessarily argue against biological relevance. Most of the interpretable retinal variables available in our study were vascular measurements, whereas the deep learning model was trained directly on raw retinal images and may capture more complex, distributed, and higher-dimensional patterns that are not reducible to a limited set of handcrafted features. This is a well-recognized characteristic of deep learning-derived imaging biomarkers across ophthalmology and medical imaging more broadly. Interestingly, the weak correlations with vascular features may suggest that RAG captures information extending beyond classical retinal vascular aging markers. In addition, model attention maps consistently highlighted regions around the macula and optic disc, suggesting that these areas may contribute importantly to the learned signal. As the optic disc contains neural structures, it is possible that part of the information captured by the model relates to neuroretinal changes, which could potentially be relevant to associations observed with dementia [11,12]. However, these interpretations remain speculative and will require dedicated investigations. Ongoing work in our group is further investigating which retinal image features and regions are most informative for different aging-related phenotypes.

Finally, we note that if RAG primarily reflected random residual variation or noise, it would be difficult to reconcile this with its associations across systemic biomarkers and clinical outcomes, as well as its substantial SNP-based heritability (~28%) and multiple significant genetic associations identified in our GWAS analyses. Together, these findings support the interpretation that RAG captures structured and biologically relevant variation, even if the precise underlying mechanisms remain to be fully elucidated.

2. Concerns regarding the supervision signal and the training objective

The authors clarify that the dataset includes participants with diagnosed systemic and ocular diseases, which is helpful. However, this raises a more fundamental concern regarding the training objective. The model is trained to predict chronological age using a mean absolute error (MAE) loss. Under this setup, individuals with disease-related retinal changes are still assigned their chronological age as the ground truth. As a result, the model is encouraged to fit these cases as accurately as possible, which would tend to drive their predicted age and thus their RAG toward zero. This creates a conceptual tension: if disease-related deviations are already absorbed into the training target, it is unclear how RAG can later be interpreted as capturing biologically meaningful deviations from normal aging. In other words, the current framework may implicitly treat disease-associated variation as part of the "normal" aging signal during training, rather than learning a clean aging trajectory. This raises concerns about whether RAG reflects true biological aging differences or artifacts of the training setup.

Response:
We thank the Reviewer for this thoughtful comment and agree that it highlights an important conceptual aspect of age-prediction frameworks. Indeed, when models are trained to predict chronological age, disease-related image features present in the training data may partially contribute to the learned age representation. We agree that this creates an inherent tension in interpreting age-gap phenotypes and have revised the manuscript to discuss this limitation more explicitly.

(Discussion/Limitations):
"Conversely, because the model is trained to predict chronological age, disease-related retinal features present in the training data may be incorporated into the learned age representation, creating an inherent tension in the interpretation of the RAG phenotype, which likely reflects a mixture of normative aging processes and disease-related changes."

More specifically, we understand the Reviewer's concern that if disease-associated retinal changes are present in individuals of a given chronological age, the model may partially learn these features as part of the expected age-related appearance. In principle, this could attenuate the residual signal in diseased individuals by shifting predicted age closer to chronological age, rather than producing large positive RAG values.

One possible strategy to address this issue would have been to exclude participants with diagnosed diseases from the training dataset, as has been done in some age-gap studies

aiming to approximate a "healthy aging" trajectory [5,8–10,13,14]. However, while this approach reduces the available training sample size, potentially limiting model performance, we believe it does not fully resolve the underlying conceptual problem. In fact, many aging-related processes exist along a continuum, with pathological changes progressively accumulating long before clinical diagnosis. For example, lens opacity increases gradually with age, whereas cataract diagnosis reflects only the clinically overt end of this spectrum. Thus, excluding individuals with diagnosed cataract would still leave many individuals with subclinical or undiagnosed lens changes in the training data. Similar considerations likely apply to vascular, neurodegenerative, and metabolic retinal alterations.

For this reason, we chose not to filter the training data based on disease status. Instead, the model was trained on the full spectrum of retinal aging-related variation present in the population, ranging from apparently healthy to subclinical and clinically manifest changes. In this context, we view RAG not as deviation from an idealized "healthy aging" trajectory, but rather as deviation from the population-average relationship between retinal appearance and chronological age. We have further clarified this in the manuscript.

(Discussion):
"The resulting retinal age gap (RAG)—the difference between predicted retinal age and chronological age—represents individual deviation from the average relationship between retinal appearance and chronological age in the reference population."

We also note that diagnosed ocular diseases represented a relatively small proportion of the dataset (ranging from 1.2% for AMD to 5% for cataract; see Table S3), reducing the likelihood that these cases disproportionately shaped the learned representation. In addition, the use of MAE loss reduces sensitivity to outliers compared with MSE-based optimization.

More broadly, we believe this discussion reflects a fundamental challenge in aging research rather than a problem specific to our study or to age-prediction models alone: namely, how to define "healthy," "normal," and "pathological" aging in biological systems that change continuously over time. We do not claim that our framework resolves this issue, but rather that it represents one pragmatic and commonly used approach for studying aging-associated variation at the population level.

3. Limited incremental clinical utility

While RAG is statistically associated with multiple outcomes, its practical predictive value appears modest. Likelihood ratio tests are consistently significant, but this is expected in large datasets and does not necessarily indicate meaningful improvement in prediction. Gains in discrimination (e.g., C-index) are small and often not statistically significant, as acknowledged by the authors. Furthermore, leave-one-out analyses suggest that chronological age remains the dominant contributor to model performance, with RAG providing only incremental information.

Taken together, these findings suggest that RAG captures some residual signal; however, its added value beyond established predictors, particularly chronological age, appears limited. It therefore remains unclear whether RAG meaningfully enhances clinical risk stratification or largely recapitulates information already contained in existing variables.

Response:

While it is true that chronological age is by far the strongest predictor of overall mortality and most diseases, we do not share the assessment that RAG only provides incremental information and that modest changes in C-index necessarily imply limited biological or clinical relevance. Rather, as we mention in the manuscript, the C-index can be over-conservative when strong predictors such as chronological age and sex are already included in the baseline model [15]. Because the C-index is a rank-based metric, a new predictor may meaningfully improve risk estimation while producing only small changes in C-index if it does not substantially reorder individuals relative to an already strong baseline model. For this reason, it has been argued that exclusive reliance on C-statistics may underestimate the relevance of novel predictors in epidemiological studies [15,16].

Importantly, chronological age being the dominant predictor of mortality and major age-related diseases is not surprising, but rather expected. To our knowledge, no age-independent biomarker has been shown to approach the predictive contribution of chronological age itself for outcomes such as mortality or age-related diseases. In this context, we found it notable that RAG emerged among the top contributors in leave-one-out analyses, ranking fourth for all-cause mortality and fifth for dementia, stroke, COPD, and cancer. In these models, RAG ranked just after age and a small number of major established risk factors such as smoking and socioeconomic status, while exceeding the contribution of several conventional risk factors including diabetes, hypertension, cholesterol, and BMI (see Fig. 3b-c).

We believe an important conceptual distinction is that RAG, as an "age-gap" phenotype, is by definition orthogonal to chronological age. Consequently, any prognostic information carried by RAG reflects variance that is independent of chronological age itself. This differs from some biological aging markers, such as epigenetic clocks, which are highly correlated with chronological age and therefore derive a substantial proportion of their predictive performance from shared age-related variance. In contrast, the effects reported for RAG represent incremental information beyond age by construction.

For these reasons, we believe it is both expected and methodologically appropriate that the effect sizes associated with RAG are substantially smaller than those of chronological age. However, we agree that our findings do not support direct clinical utility of RAG for risk prediction, and we have therefore removed statements suggesting such applicability and revised our interpretation accordingly.

(Abstract):
"Our study positions the retinal age gap as a biologically relevant and sex-specific phenotype associated with multiple aging-related diseases and outcomes beyond conventional risk factors, including chronological age."

(Discussion):
"Overall, our study demonstrates that RAG represents a non-invasive retinal imaging-derived aging-associated phenotype linked to ocular, cardiovascular, metabolic, inflammatory, and cognitive dimensions of aging. Our findings highlight the potential of retinal imaging as an accessible tool for capturing systemic aging signals. Importantly, RAG is constructed to be orthogonal to chronological age, thereby capturing variation in retinal appearance beyond that explained by age and providing complementary information to conventional clinical risk factors. "

References

1. Franke K, Gaser C. Ten years of BrainAGE as a neuroimaging biomarker of brain aging: What insights have we gained? Front Neurol. 2019;10: 789. doi:10.3389/fneur.2019.00789

2. Liu S, Xu L, Xu G, Wang Y, Zhang G, He L. A systematic review: Brain age gap as a promising early diagnostic biomarker for Alzheimer's disease. J Neurol Sci. 2025;475: 123563. doi:10.1016/j.jns.2025.123563

3. Cole JH. Multimodality neuroimaging brain-age in UK biobank: relationship to biomedical, lifestyle, and cognitive factors. Neurobiol Aging. 2020;92: 34–42. doi:10.1016/j.neurobiolaging.2020.03.014

4. Grimbly MJ, Koopowitz S-M, Chen R, Sun Z, Foster PJ, He M, et al. Estimating biological age from retinal imaging: a scoping review. BMJ Open Ophthalmol. 2024;9: e001794. doi:10.1136/bmjophth-2024-001794

5. MULTI Consortium, Cao H, Song Z, Duggan MR, Erus G, Srinivasan D, et al. MRI-based multi-organ clocks for healthy aging and disease assessment. Nat Med. 2026;32: 82–92. doi:10.1038/s41591-025-03999-8

6. Le Goallec A, Collin S, Diai S, Prost J-B, Jabri M 'hamed, Vincent T, et al. Analyzing the multidimensionality of biological aging with the tools of deep learning across diverse image-based and physiological indicators yields robust age predictors. medRxiv. 2021. doi:10.1101/2021.04.25.21255767

7. Argentieri MA, Xiao S, Bennett D, Winchester L, Nevado-Holgado AJ, Ghose U, et al. Proteomic aging clock predicts mortality and risk of common age-related diseases in diverse populations. Nat Med. 2024;30: 2450–2460. doi:10.1038/s41591-024-03164-7

8. MULTI consortium, Anagnostakis F, Ko S, Saadatinia M, Wang J, Davatzikos C, et al. Multi-organ metabolome biological age implicates cardiometabolic conditions and mortality risk. Nat Commun. 2025;16: 4871. doi:10.1038/s41467-025-59964-z

9. Wen J. Refining the generation, interpretation and application of multi-organ, multi-omics biological aging clocks. Nat Aging. 2025;5: 1897–1913. doi:10.1038/s43587-025-00928-9

10. MULTI Consortium, O'Toole CK, Song Z, Anagnostakis F, Yang Z, Tian YE, et al. Sleep chart of biological ageing clocks in middle and late life. Nature. 2026. doi:10.1038/s41586-026-10524-5

11. Tsai CS, Ritch R, Schwartz B, Lee SS, Miller NR, Chi T, et al. Optic nerve head and nerve

fiber layer in Alzheimer's disease. Arch Ophthalmol. 1991;109: 199–204. doi:10.1001/archopht.1991.01080020045040

12. Gibbon S, Breen DP, MacGillivray TJ, UK Biobank Eye & Vision Consortium. Optic disc pallor in Parkinson's disease: A UK Biobank study. Mov Disord. 2025;40: 1123–1133. doi:10.1002/mds.30127
13. Kim YD, Noh KJ, Byun SJ, Lee S, Kim T, Sunwoo L, et al. Effects of hypertension, diabetes, and smoking on age and sex prediction from retinal fundus images. Sci Rep. 2020;10: 4623. doi:10.1038/s41598-020-61519-9
14. Zhu Z, Shi D, Guankai P, Tan Z, Shang X, Hu W, et al. Retinal age gap as a predictive biomarker for mortality risk. Br J Ophthalmol. 2023;107: 547–554. doi:10.1136/bjophthalmol-2021-319807
15. Zoccali C, Tripepi G. The conservativeness of standard C statistics in the prediction of clinical events. Eur J Clin Invest. 2026;56: e70150. doi:10.1111/eci.70150
16. Hartman N, Kim S, He K, Kalbfleisch JD. Pitfalls of the concordance index for survival outcomes. Stat Med. 2023;42: 2179–2190. doi:10.1002/sim.9717

Reviewer #3 (Remarks to the Author):

The authors have made a careful and useful revision in response to the conceptual issues raised by Reviewer #2. Reframing the retinal age gap (RAG) as a deviation from a reference population's retinal ageing trajectory, rather than as a direct or absolute measure of biological age, is an important and appropriate correction. I agree that the residual-age framework is not conceptually invalid. Chronological age is an imperfect but practical supervisory signal, provided that the resulting residual is interpreted strictly as an age-orthogonal retinal imaging phenotype.

The additional analyses and clarifications substantially strengthen the manuscript. These include the clarification of age-bias correction, likelihood-ratio tests, C-index comparisons, leave-one-out contribution analyses, and stratification of female participants by menopausal status and age. These additions address several of the key concerns raised by Reviewer #2, particularly whether RAG simply recapitulates chronological age and whether the sex-specific findings have a plausible biological interpretation.

Response:
We thank the Reviewer for this assessment and are happy that we were successful in addressing previous concerns adequately.

However, the conceptual framing still remains too strong in several places and should be further qualified.

1. Clinical utility and effect sizes

The data support RAG as a promising candidate retinal imaging biomarker associated with ageing-related clinical traits. They do not establish RAG as a clinically useful risk prediction tool or as ready for personalized risk assessment. The improvements in model discrimination, including C-index changes, appear limited for several outcomes, and the effect sizes are generally modest. The manuscript should therefore soften statements that imply established clinical utility.

Response:
We thank the Reviewer for these pertinent comments with which we fully agree. We have revised the manuscript to further qualify the interpretation of RAG as a retinal imaging-derived aging-associated phenotype and removed statements suggesting direct clinical utility for risk prediction.

(Abstract):
"Our study positions the retinal age gap as a biologically relevant and sex-specific phenotype associated with multiple aging-related diseases and outcomes beyond conventional risk factors, including chronological age."

(Discussion):
"Overall, our study demonstrates that RAG represents a non-invasive retinal imaging-derived aging-associated phenotype linked to ocular, cardiovascular, metabolic, inflammatory, and cognitive dimensions of aging. Our findings highlight the potential of retinal imaging as an accessible tool for capturing systemic aging signals. Importantly, RAG is constructed to be orthogonal to chronological age, thereby capturing variation in retinal appearance beyond that explained by age and providing complementary information to conventional clinical risk factors. "

2. Biological specificity and the OCA2 signal

The biological specificity of the RAG signal warrants a more critical discussion. The prominent GWAS signal involving OCA2 is particularly noteworthy, given its role in ocular pigmentation. Together with the latent-variable correlations with RGB and intensity-related features, this raises a plausible concern that part of the model-derived retinal age signal may reflect fundus pigmentation, color distribution, image brightness, or acquisition-related characteristics, rather than ageing biology alone. This does not invalidate the findings, but it is directly relevant to the construct validity and generalizability of RAG. The Limitations section should clearly state that genetic loci associated with RAG cannot be assumed to reflect ageing-related pathways alone.

Response:
We thank the Reviewer for this important point. We have revised the manuscript to more explicitly acknowledge that part of the retinal age signal may reflect non-aging-related image characteristics, including pigmentation- and intensity-related properties, and clarified that genetic loci associated with RAG should not be interpreted exclusively as reflecting aging-related biological pathways.

(Discussion):

"Like previous retinal age studies [17–19], we also identified OCA2, a gene involved in eye pigmentation, suggesting that image features that are unlikely to be age-relevant (e.g. fundus pigmentation, color distribution, or image brightness) may influence the model prediction."

(Discussion/Limitations):

"Part of the retinal age signal may reflect non-aging-related image characteristics, as suggested by associations with OCA2 and intensity-related image features, and therefore genetic loci associated with RAG should not be interpreted exclusively as reflecting aging-related biological pathways."

3. Distinguishing the age-prediction model from the residual RAG phenotype

The manuscript should more clearly distinguish the age-prediction model from the residual RAG phenotype. The saliency and attention analyses suggest that the age-prediction model attends to the macula, optic disc, and vascular regions, among other features. In contrast, the updated feature-correlation analyses suggest that the residual RAG phenotype is only weakly explained by the predefined vascular and color features examined. These findings are not contradictory, because they refer to different levels of analysis. However, the text should avoid implying that RAG is independent of vascular ageing in general. A more cautious interpretation would be that RAG is not strongly explained by the explicit vascular and color features examined here, while more complex, nonlinear, or spatially localized vascular contributions may remain.

Response:

We thank the Reviewer for this important clarification. We have revised the manuscript to more clearly distinguish findings related to the age-prediction model from those related to the residual RAG phenotype, which are now discussed in separate paragraphs. We also clarified that the weak correlations between RAG and the predefined vascular and color features examined here do not imply independence from vascular aging more broadly, as more complex or spatially localized vascular patterns may still contribute to the residual signal.

(Results):

"By contrast, correlations between these features and RAG were weak (rightmost column of Fig. 6), indicating that the residual retinal age signal captured by RAG is not directly explained by the explicit vascular and color features examined here. This does not exclude the possibility that more complex, nonlinear, or spatially localized vascular patterns contribute to RAG but are not adequately captured by the predefined features analyzed in this study."

4. Nuance in the sex-specific interpretation

The sex-specific narrative also needs refinement. Stating simply that male retinas appear younger may obscure the pattern shown in the revised analyses, where the observed difference appears to be driven at least partly by higher RAG in older or postmenopausal females, with males lying between younger and older female strata. The authors should frame this as a life-stage-dependent pattern rather than a simple binary male-versus-female difference. If the authors wish to maintain a strong sex-difference interpretation, stratifying males by the same age threshold or formally testing sex-by-age-group interactions would be helpful.

Response:
We agree that our initial formulation suggesting that male retinas appeared younger than female retinas was overly simplistic. This wording remained from earlier analyses performed prior to Reviewer #1's suggestion to stratify females by menopausal status. The additional stratified analyses substantially refined our interpretation of the sex-specific findings by showing that the observed pattern was driven largely by higher RAG in older or postmenopausal females. We therefore removed statements implying a simple binary sex difference and revised the manuscript to emphasize the life-stage-dependent nature of these patterns.

(Abstract):
"Sex-stratified models revealed consistent performance but divergent biological signatures: males had stronger links to metabolic syndrome, while in females, both model attention and genetics pointed to a greater involvement of retinal vasculature. Additional analyses indicated that retinal aging patterns in females vary across the menopausal transition, with postmenopausal females exhibiting higher retinal age gap values and clinical associations that more closely resemble those observed in males."

(Discussion):
"We initially observed a modest but consistent difference in predicted retinal age between sexes across cohorts, with male retinas estimated as appearing younger on average, even after adjusting for potential body size confounding. However, the stratified analyses suggested a more nuanced, life-stage-dependent pattern. Specifically, RAG was lower in younger or premenopausal females but higher in older or postmenopausal females, placing males between these two groups. This pattern parallels epidemiological observations in which females experience lower cardiometabolic risk prior to menopause, followed by a marked increase afterward, including a rise in CVD incidence [20]."

5. Methodological transparency relevant to interpretation

Several methodological points should be clarified because they affect how the residual phenotype should be interpreted. The manuscript should explicitly state which RAG measure was used for each downstream analysis, for example whether it was based on a single eye, both-eye average, or all available images at the first imaging instance. It should also clarify whether combined-sample and sex-stratified analyses used RAG derived from the combined model or from sex-

specific models. Finally, the description of covariate adjustment, including height and height squared, should be made consistent across cross-sectional and survival analyses.

Response:
We are grateful to the Reviewer for identifying missing methodological details in our Methods section. We have now clarified these aspects in the revised manuscript.

1) RAG measure used in downstream analyses

We first predicted chronological age for each retinal image passing quality control (out-of-sample image-level prediction). We then averaged predictions across both eyes for each participant at a given imaging visit when both were available, and otherwise we used the single available eye prediction (subject-level prediction). We subsequently applied regression-to-the-mean bias correction to these eye-averaged subject-level predictions. Finally, we defined the retinal age gap (RAG) as the difference between this bias-corrected predicted age and chronological age, and used this corrected subject-level RAG measure in all downstream analyses.

(Methods):
"For each participant at a given imaging visit, we averaged predicted ages across both eyes when available; otherwise we used the prediction from the single available eye." (...) "To correct for regression-to-the-mean bias in predicted age, we applied a post hoc adjustment to the eye-averaged predicted ages following the procedure described by Cole et al. [21]." (...) "We then defined the retinal age gap (RAG) as the difference between the corrected predicted age and the participant's chronological age. All subsequent analyses were performed using this corrected RAG measure."

2) Combined vs sex-specific models

We have now clarified throughout the manuscript that combined-sample analyses were performed using RAG derived from the combined model, whereas sex-stratified analyses and sex-specific GWAS used RAG derived from the corresponding sex-specific models.

(Methods, "Retinal age gap"):
"To assess sex differences, we compared mean RAG values derived from the combined-sample model between females and males using independent-sample t-tests and quantified the effect size with Cohen's d."

(Methods, "Association with health and lifestyle factors"):
"Additionally, using RAG derived from the sex-specific models, we conducted sex-stratified analyses to explore differential associations, adjusting for the same covariates as in the combined-sample analyses except sex."

(Methods, "Survival analysis"):
"We conducted analyses in the full sample using RAG derived from the combined model, and sex-stratified analyses using RAG derived from the corresponding sex-specific models."

(Methods, "Genome-wide association study"):
"For sex-specific GWAS analyses, we used RAG derived from the corresponding sex-specific models and applied the same covariates excluding sex. "

3) Covariate adjustment in cross-sectional and survival analyses

We thank the Reviewer for highlighting this difference in covariate specification. Cross-sectional analyses included squared terms for age and height (as well as genetic principal components alongside self-reported ethnicity), to allow more flexible adjustment for potential nonlinear and ancestry-related effects. In the survival analyses, we used a more parsimonious adjustment set because these models additionally included several established clinical risk factors and previous disease history variables, while some outcomes had relatively limited event counts, particularly in sex-stratified analyses. To ensure a sufficient number of events per variable, we therefore included only the corresponding linear covariates in the Cox models (i.e. dropping age-squared, height-squared, and the 10 first genetic PCs), particularly given the high correlation between the squared and linear terms. We clarified this in the Methods section.

(Methods, "Survival analysis"):
"We adjusted models for age, sex (combined analyses only), height, ethnicity, education, income, Townsend, SBP, HDL, total cholesterol, smoking status, BMI, diabetes, BP medication, and previous events from the same category (for CVDs and cancer only). Thus, compared with the cross-sectional analyses, the Cox models additionally included established clinical risk factors and prior disease history to assess the predictive value of RAG beyond conventional risk factors. Because several outcomes, particularly in sex-stratified analyses, had relatively limited numbers of incident events, we used a more parsimonious covariate specification in the Cox models to limit model complexity and potential overfitting [22], and therefore did not include squared terms for age and height or genetic PCs."

In summary, this is a solid exploratory study of a retinal imaging biomarker. The authors have substantially addressed Reviewer #2's conceptual concerns, but the manuscript should more clearly frame RAG as a candidate age-orthogonal retinal imaging phenotype, not as a direct measure of biological ageing or an established clinically useful risk prediction tool. With this more cautious framing, the conceptual basis of the study would be sound and appropriately scoped.

Response:
We thank the Reviewer for this thoughtful assessment and constructive feedback. In response, we have further refined the conceptual framing of RAG, qualified its biological interpretation, and tempered statements regarding its clinical utility. We hope that these revisions adequately address the Reviewer's concerns.

References

17. Ahadi S, Wilson KA, Babenko B, McLean CY, Bryant D, Pritchard O, et al.

Longitudinal fundus imaging and its genome-wide association analysis provide evidence for a human retinal aging clock. Elife. 2023;12. doi:10.7554/eLife.82364

18. Le Goallec A, Diai S, Collin S, Vincent T, Patel CJ. Deep learning of fundus and optical coherence tomography images enables identification of diverse genetic and environmental factors associated with eye aging. medRxiv. medRxiv; 2021. doi:10.1101/2021.06.24.21259471

19. Huang Y, Syed MG, Chen R, Li C, Shang X, Wang W, et al. Genomic determinants of biological age estimated by deep learning applied to retinal images. GeroScience. 2025;47: 2613–2629. doi:10.1007/s11357-024-01481-w

20. Fasero M, Coronado PJ. Cardiovascular disease risk in women with menopause. J Clin Med. 2025;14: 3663. doi:10.3390/jcm14113663

21. Cole JH, Ritchie SJ, Bastin ME, Valdés Hernández MC, Muñoz Maniega S, Royle N, et al. Brain age predicts mortality. Mol Psychiatry. 2018;23: 1385–1392. doi:10.1038/mp.2017.62

22. Concato J, Peduzzi P, Holford TR, Feinstein AR. Importance of events per independent variable in proportional hazards analysis. I. Background, goals, and general strategy. J Clin Epidemiol. 1995;48: 1495–1501. doi:10.1016/0895-4356(95)00510-2

We would like to sincerely thank all three Reviewers for their time, expertise, and thoughtful evaluation of our manuscript throughout the review process. We are particularly grateful for the constructive feedback, which has greatly improved the rigor, clarity, and interpretation of our work.

We have addressed all remaining points raised by Reviewer #3, as detailed below.

1. Please further soften the wording around biological ageing and clinical utility in the Introduction and conclusion. The Abstract and much of the Discussion now read appropriately, but the Introduction still contains phrases such as "promising marker of biological aging" and "risk stratification in both clinical and population-level health settings," and the stated aim still refers to "assess their clinical utility." Given the modest effect sizes and the limited improvements in discrimination, more cautious wording would be preferable—for example, "candidate retinal imaging-derived aging-associated phenotype" and "potential relevance for future risk stratification research."

Response: We thank the Reviewer for identifying these remaining statements, which had escaped our attention. We have revised the Introduction to further soften the wording regarding biological ageing and clinical utility, following the Reviewer's suggestions.

(Introduction):
Importantly, the difference between predicted age and true age, known as retinal age gap (RAG), has emerged as a promising phenotype for studying aging-related variation beyond chronological age.
(...)
Unlike many established aging biomarkers that require more invasive procedures or costly laboratory assays, retinal age can be estimated cheaply and non-invasively from fundus photographs. This makes retinal imaging a highly accessible and scalable modality for investigating aging-related variation and its potential relevance for future risk stratification research.
(...)
Through these analyses, we aim to better characterize the retinal age gap phenotype, evaluate its associations with aging-related traits and outcomes, and investigate how these associations differ between females and males.

2. I would suggest softening the use of "predicts" in the survival-analysis section. The prospective associations are important, but the results do not establish RAG as a clinically useful prediction tool. The heading "Retinal age gap predicts mortality and incident disease" might be changed to "Retinal age gap is prospectively associated with mortality and incident disease," which would better align with the cautious framing adopted elsewhere.

Response: We have revised the section heading according to the Reviewer's suggestion to better reflect the observational nature of the analyses.

(Results header):
Retinal age gap is prospectively associated with mortality and incident disease with sex-specific patterns

3. Please check the notation and algebraic specification of the age-bias correction formula. In Methods, p.15, lines 536–537, predicted age is said to be regressed on chronological age to estimate the slope and intercept, but both quantities appear to be denoted by the same symbol. The subsequent statement that predicted age was adjusted by "subtracting and dividing by" is consequently ambiguous. Please state the fitted linear model and the correction formula explicitly.

Response: We thank the Reviewer for pointing out this ambiguity. We have revised the Methods to explicitly state the fitted linear model used to estimate the age-bias correction parameters, the corresponding correction formula, and the final retinal age gap (RAG) formula, thereby clarifying the notation.

(Methods):
Specifically, we fit a linear regression:

$$\hat{Y} = \alpha Y + \beta + \varepsilon \quad (1)$$

where $\hat{Y}$ is predicted age and Y chronological age, then corrected the predicted age as follows:

$$\hat{Y}_{corr} = \frac{\hat{Y} - \beta}{\alpha} \quad (2)$$

We then defined the retinal age gap (RAG) as:

$$RAG = \hat{Y}_{corr} - Y \quad (3)$$

All subsequent analyses were performed using this corrected RAG measure.

4. Please temper the final statement on precision medicine. The sex-stratified and menopause-stratified analyses are valuable, but the current data do not establish direct clinical implementation. "Advancing their application in precision medicine" could be softened to something like "informing future biomarker development" or "guiding future studies of sex-specific ageing biomarkers."

Response: We thank the Reviewer for this suggestion. We have revised the final sentence of the Discussion to avoid implying direct clinical implementation and to more appropriately frame the implications of our findings for future research.

(Discussion):

Incorporating sex-specific perspectives will therefore be essential for refining aging biomarkers and guiding future studies of sex-specific aging processes.
